# Bilateral gene therapy in children with autosomal recessive deafness 9: single-arm trial results

**DOI:** 10.1038/s41591-024-03023-5

**Published:** 2024-06-05

**Authors:** Hui Wang, Yuxin Chen, Jun Lv, Xiaoting Cheng, Qi Cao, Daqi Wang, Longlong Zhang, Biyun Zhu, Min Shen, Chunxin Xu, Mengzhao Xun, Zijing Wang, Honghai Tang, Shaowei Hu, Chong Cui, Luoying Jiang, Yanbo Yin, Luo Guo, Yi Zhou, Lei Han, Ziwen Gao, Jiajia Zhang, Sha Yu, Kaiyu Gao, Jinghan Wang, Bing Chen, Wuqing Wang, Zheng-Yi Chen, Huawei Li, Yilai Shu

**Affiliations:** 1 ENT Institute and Otorhinolaryngology Department of Eye & ENT Hospital, Fudan University, Shanghai, China; 2 NHC Key Laboratory of Hearing Medicine, Fudan University, Shanghai, China; 3 Institutes of Biomedical Sciences, Fudan University, Shanghai, China; 4 State Key Laboratory of Medical Neurobiology and MOE Frontiers Center for Brain Science, Fudan University, Shanghai, China; 5 Department of Otorhinolaryngology, the Second Affiliated Hospital, Hengyang Medical School, University of South China, Hengyang, Hunan, China; 6 Shanghai Rehabilitation Institute for the Exceptional Children, Shanghai, China; 7 Shanghai Refreshgene Therapeutics Co. Ltd., Shanghai, China; 8 Department of Otolaryngology—Head and Neck Surgery, Graduate Program in Speech and Hearing Bioscience and Technology and Program in Neuroscience, Harvard Medical School, Boston, MA USA; 9 Eaton-Peabody Laboratory, Massachusetts Eye and Ear, Boston, MA USA

**Keywords:** Neurological disorders, Gene therapy

## Abstract

Gene therapy is a promising approach for hereditary deafness. We recently showed that unilateral AAV1-hOTOF gene therapy with dual adeno-associated virus (AAV) serotype 1 carrying human *OTOF* transgene is safe and associated with functional improvements in patients with autosomal recessive deafness 9 (DFNB9). The protocol was subsequently amended and approved to allow bilateral gene therapy administration. Here we report an interim analysis of the single-arm trial investigating the safety and efficacy of binaural therapy in five pediatric patients with DFNB9. The primary endpoint was dose-limiting toxicity at 6 weeks, and the secondary endpoint included safety (adverse events) and efficacy (auditory function and speech perception). No dose-limiting toxicity or serious adverse event occurred. A total of 36 adverse events occurred. The most common adverse events were increased lymphocyte counts (6 out of 36) and increased cholesterol levels (6 out of 36). All patients had bilateral hearing restoration. The average auditory brainstem response threshold in the right (left) ear was >95 dB (>95 dB) in all patients at baseline, and the average auditory brainstem response threshold in the right (left) ear was restored to 58 dB (58 dB) in patient 1, 75 dB (85 dB) in patient 2, 55 dB (50 dB) in patient 3 at 26 weeks, and 75 dB (78 dB) in patient 4 and 63 dB (63 dB) in patient 5 at 13 weeks. The speech perception and the capability of sound source localization were restored in all five patients. These results provide preliminary insights on the safety and efficacy of binaural AAV gene therapy for hereditary deafness. The trial is ongoing with longer follow-up to confirm the safety and efficacy findings. Chinese Clinical Trial Registry registration: ChiCTR2200063181.

## Abstract (web summary)

An interim analysis of a single-arm trial in 5 children with hereditary deafness shows that binaural AAV gene therapy is safe and leads to hearing improvement up to 13–26 weeks of follow-up.

## Main

According to the World Health Organization, over 5% of the global population, or 430 million people, suffer from disabling hearing loss, including 34 million children^1^. There are about 26 million people with congenital hearing loss, of which 60% is attributed to genetic factors^2,3^. The deficient or dysfunctional otoferlin protein results from pathogenic mutations in the *OTOF* gene and leads to autosomal recessive deafness 9 (DFNB9)^4^. DFNB9 is characterized by congenital or prelingual, severe-to-complete bilateral hearing loss and accounts for 2–8% of hereditary deafness^5–9^.

Adeno-associated virus (AAV) serotype 1 carrying human *OTOF* transgene (AAV1-hOTOF) coding the human functional otoferlin protein driven by a hair cell-specific promoter has been shown to be effective and safe in *Otof*^−*/*−^ mice and nonhuman primates^10^. An ongoing trial from our group has shown the safety and efficacy of unilateral gene therapy in children with DFNB9 (ref. ^11^). However, compared with unilateral hearing, restoration of hearing bilaterally will probably bring greater benefits to patients including better speech perception in the noise environment and the ability to localize the sound source^12,13^. Hence, it is imperative to restore the hearing in both ears of patients with bilateral deafness to maximize the benefits of hearing recovery.

A major challenge of AAV-mediated gene therapy is preexisting anti-AAV neutralizing antibodies after the initial AAV infection, which may prevent subsequent AAV vectors from infecting target tissues and cells, cause immunotoxicology and restrict repeat administration of the AAV vector owing to immune clearance, thus greatly reducing the treatment efficacy^14–19^. The bilateral injection of AAV vector in a one-time surgery could ameliorate the potential risks associated with anti-AAV neutralizing antibodies. We have conducted *OTOF* gene therapy in DFNB9 patients with hearing recovery by unilateral ear injection^11^. We present here the results to show safety and efficacy with the additional benefit of sound source localization through bilateral administration of AAV1-hOTOF gene therapy in patients with DFNB9.

## Results

### Patients

**Fig. 1:**
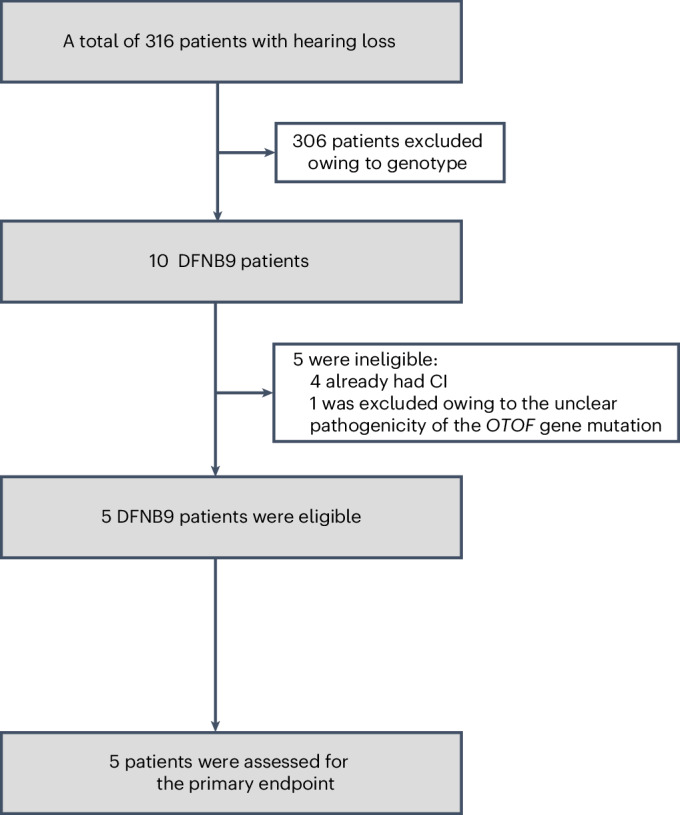
Patient enrollment. Five patients were enrolled to receive binaural gene therapy and were evaluated for the primary endpoint. CI, cochlear implant. Source data

**Extended Data Fig. 1:**
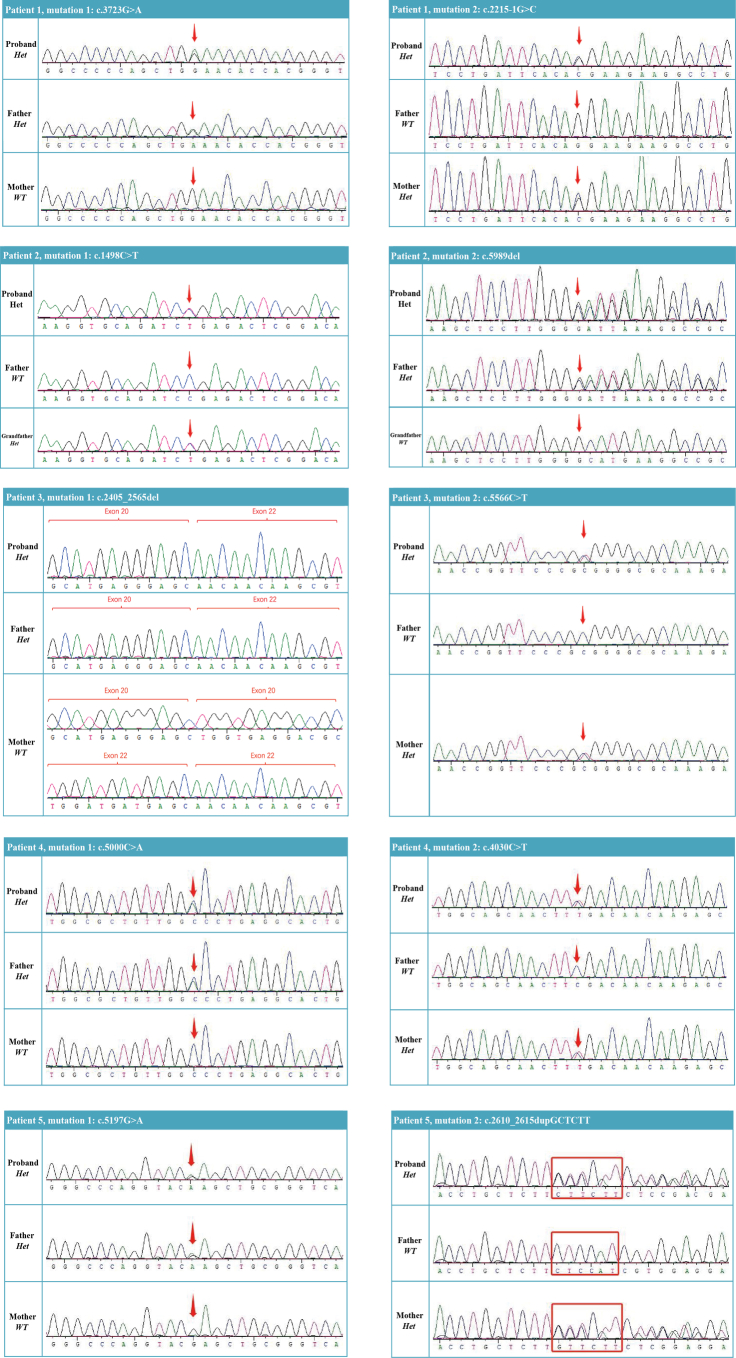
Sanger sequencing results in patients and family members. Mutation 1 indicates the mutation in *OTOF* allele 1. Mutation 2 indicates the mutation in *OTOF* allele 2. Proband indicates the patient. *Het*, heterozygous; *WT*, wildtype. The sanger sequencing results in parents of patients 1, 3, 4, and 5 are provided. The sanger sequencing result in father of patient 2 is provided. The sanger sequencing result in grandfather of patient 2 is provided, because the mother of patient 2 passed away.

We screened 316 participants for eligibility (Fig. 1). Five pediatric patients (two girls and three boys) with bilateral congenital hearing loss caused by biallelic *OTOF* mutations were enrolled from 14 July 2023 to 15 November 2023 (Fig. 1 and Table 1). Details of Sanger sequencing results and *OTOF* variant interpretation in patients are provided in Extended Data Fig. 1 and Extended Data Table 1. The average auditory brainstem response (ABR) threshold was >95 dB in all patients at baseline (Table 1). None of the patients received cochlear implants before the trial. A dose of 1.5 × 10^12^ vector genomes (vg) AAV1-hOTOF per ear, selected on the basis of the previous unilateral study^11^, was subsequently injected into the bilateral cochleae of the patient through the round window during a one-time operation. We have completed a 26-week assessment in patients 1, 2 and 3, and a 13-week assessment in patients 4 and 5. The study is ongoing.

**Table 1:** Baseline characteristics of the patients

	Patient 1	Patient 2	Patient 3	Patient 4	Patient 5
Sex	Female	Male	Male	Female	Male
Age (years)	11.0	1.2	2.6	3.1	2.8
Mutations in *OTOF*^a^					
Mutation 1	c.3723G>A (p.Trp1241*)	c.1498C>T (p.Arg500*)	c.2405_2565del (p.Leu802Glnfs*37)	c.5000C>A (p.Ala1667Asp)	c.5197G>A (p.Glu1733Lys)
Mutation 2	c.2215-1G>C	c.5989del (p.Ala1997Hisfs*68)	c.5566C>T (p.Arg1856Trp)	c.4030C>T (p.Arg1344*)	c.2610_2615dupGCTCTT (p.Leu870_Leu871dup)
ABR threshold (dB)^b^					
Left ear	>95	>95	>95	>95	>95
Right ear	>95	>95	>95	>95	>95
ASSR threshold (dB)^b^					
Left ear	103	81	100	106	88
Right ear	103	79	100	106	85

Mutation 1, mutation in *OTOF* allele 1; Mutation 2, mutation in *OTOF* allele 2.

^a^Human OTOF transcript: NM_001287489.2.

^b^Average hearing threshold at 0.5–4 kHz; ‘>95’ means no response at the maximum sound intensity level.

### Primary outcome

The primary endpoint was dose-limiting toxicity, defined as hematologic toxicity ≥ grade 4, nonhematologic toxicity ≥ grade 3 or aural toxicity ≥ grade 2 within 6 weeks. The grade was assessed according to Common Terminology Criteria for Adverse Events Version 5.0 (CTCAE V5.0). The dose of 1.5 × 10^12^ vg AAV1-hOTOF was selected for bilateral treatment based on the results of the unilateral study that tested different doses^11^. No dose-limiting toxicity happened in five patients receiving binaural gene therapy with a dose of 1.5 × 10^12^ vg AAV1-hOTOF per ear.

### Efficacy

Efficacy outcomes include auditory function and speech perception. ABR, auditory steady-state response (ASSR), distortion product otoacoustic emission (DPOAE), and related questionnaires and tests were used to evaluate the auditory function, speech perception and sound source localization in patients.

**Fig. 2:**
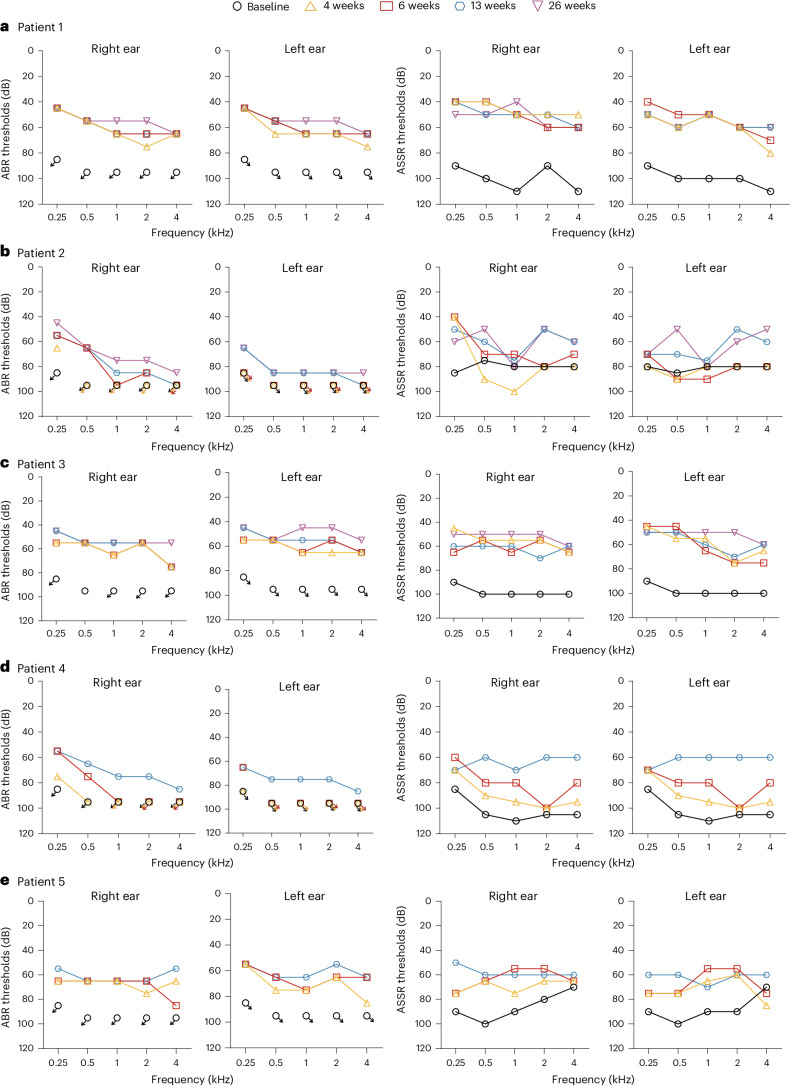
Audiometric test. **a**–**e**, The ABR and ASSR thresholds of patients 1 (**a**), 2 (**b**), 3 (**c**), 4 (**d**) and 5 (**e**). The arrows indicate no response even at the maximum sound intensity level. Arrows pointing left and downward, right ear; arrows pointing right and downward, left ear.

At baseline, the average ABR threshold in the right (left) ear was >95 dB (>95 dB) in all five patients. In patient 1, the average ABR threshold in the right (left) ear was restored to 65 dB (68 dB) at 4 weeks, 63 dB (63 dB) at 6 weeks, 63 dB (63 dB) at 13 weeks and 58 dB (58 dB) at 26 weeks; the average ASSR threshold in the right (left) ear was 103 dB (103 dB) at baseline, and was restored to 48 dB (63 dB) at 4 weeks, 53 dB (58 dB) at 6 weeks, 53 dB (58 dB) at 13 weeks and 53 dB (58 dB) at 26 weeks (Fig. 2a). In patient 2, the average ABR threshold in the right (left) ear was >95 dB (>95 dB) at 4 weeks, >85 dB (>95 dB) at 6 weeks, 83 dB (88 dB) at 13 weeks and 75 dB (85 dB) at 26 weeks; the average ASSR threshold in the right (left) ear was 88 dB (83 dB) at 4 weeks, 73 dB (85 dB) at 6 weeks, 61 dB (64 dB) at 13 weeks and 60 dB (60 dB) at 26 weeks, compared with 79 dB (81 dB) at baseline (Fig. 2b). In patient 3, the average ABR threshold in the right (left) ear was restored to 63 dB (63 dB) at 4 weeks, 63 dB (60 dB) at 6 weeks, 60 dB (58 dB) at 13 weeks and 55 dB (50 dB) at 26 weeks; the average ASSR threshold in the right (left) ear was restored to 58 dB (63 dB) at 4 weeks, 60 dB (65 dB) at 6 weeks, 63 dB (60 dB) at 13 weeks and 53 dB (53 dB) at 26 weeks, compared with 100 dB (100 dB) at baseline (Fig. 2c). In patient 4, the average ABR threshold in the right (left) ear was >95 dB (>95 dB) at 4 weeks, >90 dB (>95 dB) at 6 weeks and 75 dB (78 dB) at 13 weeks; the average ASSR threshold in the right (left) ear was restored to 95 dB (95 dB) at 4 weeks, 85 dB (85 dB) at 6 weeks and 63 dB (60 dB) at 13 weeks, compared with 106 dB (106 dB) at baseline (Fig. 2d). In patient 5, the average ABR threshold in the right (left) ear was restored to 68 dB (75 dB) at 4 weeks, 70 dB (68 dB) at 6 weeks and 63 dB (63 dB) at 13 weeks; the average ASSR threshold in the right (left) ear was restored to 68 dB (71 dB) at 4 weeks, 60 dB (65 dB) at 6 weeks and 60 dB (63 dB) at 13 weeks, compared with 85 dB (88 dB) at baseline (Fig. 2e).

**Extended Data Fig. 2:**
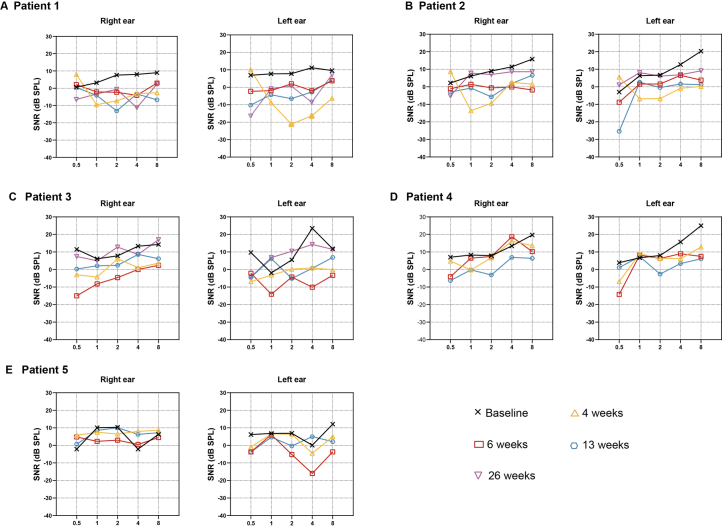
The signal-to-noise ratio of the DPOAE in patients at baseline and follow-up visits. **a–c**, In patients 1, 2 and 3, the SNR of DPOAE decreased at most of frequencies at 4 weeks and showed the tendency to recover to the baseline at following timepoints. **d**, The SNR of DPOAE in both ears of patient 4 was stable at some frequencies at 4 weeks and 6 weeks, but it decreased at most frequencies at 13 weeks. **e**, The SNR of DPOAE in patient 5 was stable at some frequencies at 4 weeks, but it decreased at some frequencies at 6 weeks and showed recovery to some extent at 13 weeks. SNR: signal-to-noise ratio. DPOAE: distortion product otoacoustic emission.

In both ears of patients 1–3, the signal-to-noise ratio (SNR) of DPOAE decreased at most frequencies at 4 weeks and gradually recovered at the later follow-up (Extended Data Fig. 2a–c). In patient 4, the SNR was stable at some frequencies at 4 weeks, decreased to some extent at later follow-up and has not recovered at 13 weeks (Extended Data Fig. 2d). In patient 5, the SNR decreased at some frequencies at 6 weeks and recovered to some degree at 13 weeks (Extended Data Fig. 2e).

In patient 1, the Meaningful Auditory Integration Scale (MAIS) and Categories of Auditory Performance (CAP) scores were 1 and 0, respectively, at baseline, and 28 and 4, respectively, at 26 weeks; the Speech Intelligibility Rating (SIR) and Meaningful Use of Speech Scale (MUSS) scores were 1 and 0, respectively, at baseline, and 1 and 7, respectively, at 26 weeks. The Speech of the Speech, Spatial, and Other Qualities of Hearing Scale for Parents (SSQ-P), the Spatial of the SSQ-P and the Other Qualities of the SSQ-P scores were 0.3, 0 and 0, respectively, at baseline, and were improved to 7.8, 2.8 and 5.0, respectively, at 26 weeks (Table 2). In a quiet environment, the perception of monosyllable, disyllable and sentence was all 0% at baseline and 2.0%, 1.4% and 0%, respectively, at 26 weeks after treatment; ambient sound, tone, initial and final was all 0% at baseline, and 31.3%, 31.3%, 20.8% and 20.8%, respectively, at 26 weeks (Extended Data Table 2). For sound source localization tests, the bilateral root mean square error (RMSE) was 92.8° ± 1.1° at baseline and 40.0° ± 1.7° at 26 weeks; when one ear was covered, the unilateral RMSE (75.5° ± 1.0°) at 26 weeks was worse (Extended Data Table 2). In Supplementary Video 1, patient 1 could not hear at baseline and could recognize sound 4 weeks and 6 weeks after injection. At 13 weeks, she could speak syllables such as ‘a’, ‘ba’ (father), ‘i’, ‘u’, ‘s’ and ‘ma’ (mother). She was able to complete the sound localization test well at 13 weeks.

**Table 2:** Scores of auditory, speech perception and sound location

		MAIS or IT-MAIS	CAP	SIR	MUSS	SSQ-P		
						Speech	Spatial	Other qualities
Patient 1	Baseline	1	0	1	0	0.3	0	0
	6 weeks	8	1	1	0	5.0	0	0
	13 weeks	17	1	1	2	7.8	1.7	2.5
	26 weeks	28	4	1	7	7.8	2.8	5.0
Patient 2	Baseline	0	0	1	0	0	0	0
	6 weeks	12	2	1	2	3.3	3.3	0.6
	13 weeks	30	4	1	4	3.3	3.3	3.8
	26 weeks	35	5	2	9	6.7	5.3	8.5
Patient 3	Baseline	0	0	1	0	0	0	0
	6 weeks	21	1	1	2	3.9	1.7	2.5
	13 weeks	32	2	1	4	5.0	4.2	5.6
	26 weeks	35	5	2	15	7.3	8.0	8.5
Patient 4	Baseline	2	0	1	2	0.3	0	0
	6 weeks	9	2	1	4	1.9	0.8	3.6
	13 weeks	16	4	1	7	3.6	5.8	4.5
Patient 5	Baseline	2	0	1	0	0.2	0	0
	6 weeks	31	3	2	7	7.4	7.0	5.6
	13 weeks	29	4	2	7	7.6	7.2	6.6

MAIS, IT-MAIS, CAP, SIR and MUSS questionnaires were used for assessment of auditory function and speech perception. SSQ-P, including Speech, Spatial and Other Qualities, was used for evaluation of sound source localization. Patients aged ≧3 years were assessed using MAIS; patients aged less than 3 years were assessed using IT-MAIS. Patients 1 and 4 were evaluated using MAIS, and patient 2 was evaluated using IT-MAIS. Patient 3 was evaluated using IT-MAIS (at baseline, 6 weeks and 13 weeks) and MAIS (at 26 weeks). Patient 5 was evaluated using IT-MAIS (at baseline and 6 weeks) and MAIS (at 13 weeks).

In patient 2, the Infant–Toddler MAIS (IT-MAIS) and CAP scores were 0 and 0, respectively, at baseline, and 35 and 5, respectively, at 26 weeks; the SIR and MUSS scores were 1 and 0, respectively, at baseline, and 2 and 9, respectively, at 26 weeks; the Speech of the SSQ-P, the Spatial of the SSQ-P and the Other Qualities of the SSQ-P scores were all 0 at baseline and 6.7, 5.3 and 8.5, respectively, at 26 weeks (Table 2). In Supplementary Video 2, patient 2 could not respond to sound and music at baseline, but he was able to turn to the sound source when his name was called from the left and right of his backward side 6 weeks after injection. He could dance to the music and complete some simple instructions at 15 weeks, and he could say some simple words, for example, ‘ayi’ (aunt) and ‘bai’ (bye), and communicate with others at 26 weeks.

In patient 3, the IT-MAIS or MAIS, and CAP, scores were all 0 at baseline, and 35 and 5, respectively, at 26 weeks; the SIR and MUSS scores were 1 and 0, respectively, at baseline, and 2 and 15, respectively, at 26 weeks; the Speech of the SSQ-P, the Spatial of the SSQ-P and the Other Qualities of the SSQ-P scores were all 0 at baseline, and 7.3, 8.0 and 8.5, respectively, at 26 weeks (Table 2). In Supplementary Video 3, patient 3 had no response to sound and music at baseline, but he could turn back when his name was called 3 weeks after injection. At 13 weeks, he was able to move his body and dance when he heard the music. He was able to say some simple words at 26 weeks, such as ‘baba’ (father), ‘nainai’ (grandmother) and ‘yeye’ (grandfather).

In patient 4, the MAIS and CAP scores were 2 and 0, respectively, at baseline, and 16 and 4, respectively, at 13 weeks; the SIR and MUSS scores were 1 and 2, respectively, at baseline, and 1 and 7, respectively, at 13 weeks; the Speech of the SSQ-P, the Spatial of the SSQ-P and the Other Qualities of the SSQ-P scores were 0.3, 0 and 0, respectively, at baseline, and 3.6, 5.8 and 4.5, respectively, at 13 weeks (Table 2). In Supplementary Video 4, patient 4 had no response to sound at baseline, but she could turn back when her name was called 4 weeks after injection. She could complete some instructions at 13 weeks, and she could say simple words at 20 weeks, for example, ‘baba’ (father), ‘mama’ (mother) and ‘nainai’ (grandmother).

In patient 5, the IT-MAIS or MAIS, and CAP, scores were 2 and 0, respectively, at baseline, and 29 and 4, respectively, at 13 weeks; the SIR and MUSS scores were 1 and 0, respectively, at baseline, and 2 and 7, respectively, at 13 weeks; the Speech of the SSQ-P, the Spatial of the SSQ-P and the Other Qualities of the SSQ-P scores were 0.2, 0 and 0, respectively, at baseline, and 7.6, 7.2 and 6.6, respectively, at 13 weeks (Table 2).

### Safety

To minimize the potential inflammatory response, dexamethasone was used intravenously for 8 days starting from 3 days before AAV1-hOTOF bilateral injection. No serious adverse event (AE) occurred. A total of 36 AEs occurred (Table 3), including emesis (patient 1), fever (patient 2), increased lymphocyte counts (patients 1–4), decreased lymphocyte counts (patient 3), decreased neutrophil counts (patient 2), decreased hemoglobin levels (patients 2 and 3), increased triglyceride levels (patient 2), increased cholesterol levels (patients 2–5), transient reduction in fibrinogen levels (patient 3), increased creatine phosphokinase levels (patient 2), decreased haptoglobin levels (patients 1 and 5), increased lactate dehydrogenase levels (patients 2–5), hyperglycemia (patient 5), proteinuria (patient 1) and hematuresis (patients 1 and 4). All 36 AEs were grade 1 or 2. The most common AEs were increased lymphocyte counts (6 out of 36) and increased cholesterol levels (6 out of 36), followed by increased lactate dehydrogenase levels (5 out of 36). In patient 1, emesis occurred at 2 h after injection and was resolved with symptomatic treatment within 1 day. In patient 2, fever (highest temperature, 38.7 °C) occurred at 18 days and 29 days after injection, with mild cough and increased lymphocyte counts, but no evidence of pneumonia or other concomitant symptoms.

**Table 3:** AEs

	Number of events	Grade	Number of patients
Any AE	36		5
Increased lymphocyte counts	6	2	4
Decreased lymphocyte counts	1	1	1
Decreased neutrophil counts	1	2	1
Decreased hemoglobin levels	2	1	2
Increased lactate dehydrogenase levels	5	1	4
Increased triglyceride levels	2	2	1
Increased cholesterol levels	5	1	4
Increased cholesterol levels	1	2	1
Decreased fibrinogen levels	1	1	1
Increased creatine phosphokinase levels	1	1	1
Decreased haptoglobin levels	2	1	2
Proteinuria	2	1	1
Hematuresis	3	1	2
Fever	2	1	1
Emesis	1	1	1
Hyperglycemia	1	1	1

**Extended Data Fig. 3:**
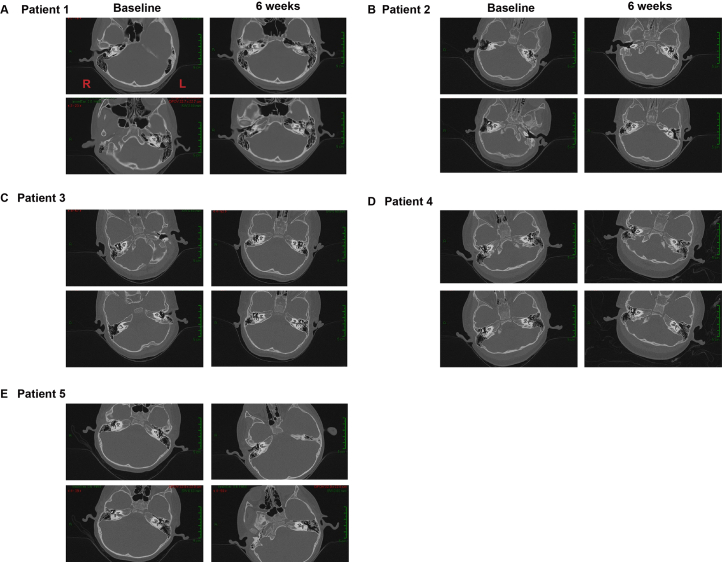
Computed tomography in patients’ ears. The images from computed tomography showed that no abnormal structure was observed in both ears of patients 1 (**a**), 2 (**b**), 3 (**c**), 4 (**d**) or 5 (**e**) at baseline and 6 weeks after gene therapy.

**Extended Data Fig. 4:**
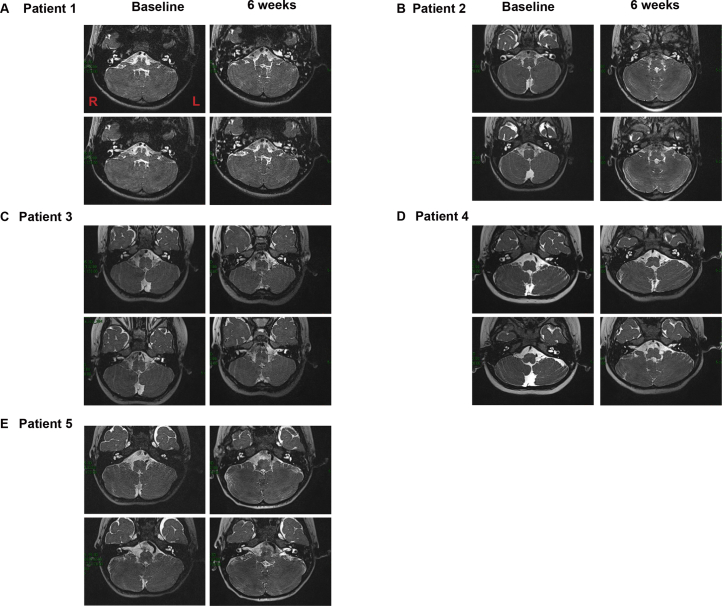
Magnetic resonance imaging in patients’ ears. **a–e**. The images from magnetic resonance imaging showed that no abnormal structure was observed in patients’ ears at baseline and 6 weeks after gene therapy.

In addition, the structure of the ears was observed by computed tomography and magnetic resonance imaging, showing the normality of the ear structure after injection (Extended Data Figs. 3 and 4).

**Extended Data Fig. 5:**
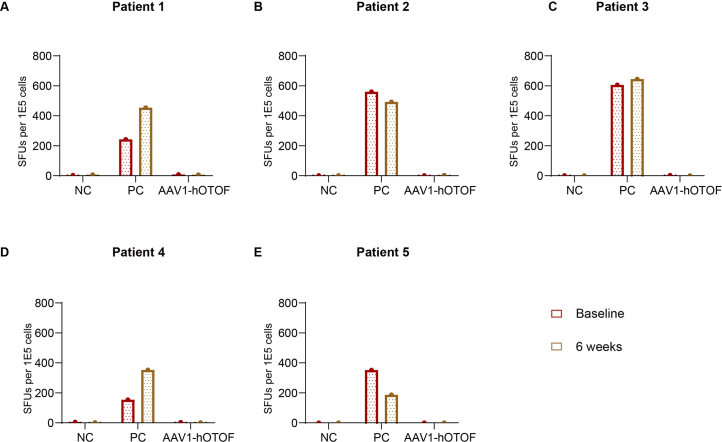
Interferon-gamma ELISpot responses to the AAV1 capsid peptide pools in patients. Interferon-gamma was detected by ELISpot assay in patients 1 (**a**), 2 (**b**), 3 (**c**), 4 (**d**), and 5 (**e**). T cell responses to the AAV1 capsid were negative in 5 patients at baseline and 6 weeks after AAV1-hOTOF gene therapy. SFU, spot-forming unit. NC: negative control; PC: positive control.

Neutralizing antibodies against AAV1 were increased in all patients at 6 weeks after treatment (Extended Data Table 3). Vector DNA in the blood was not detectable in any patient at 7 days after treatment (Extended Data Table 3). Interferon gamma (IFN-γ) enzyme-linked immunosorbent spot (ELISpot) responses to AAV1 capsid peptide pools with peripheral blood mononuclear cells (PBMCs) drawn from each patient at 6 weeks after AAV1-hOTOF binaural gene therapy were negative (Extended Data Fig. 5)

## Discussion

Here we report the results of an in-human clinical trial investigating bilateral-ear gene therapy for hearing loss. For safety, no dose-limiting toxicity or serious AEs occurred during the period of follow-up, and all 36 AEs were grade 1 or 2. For efficacy, bilateral *OTOF* gene therapy restored the bilateral hearing in all five patients; all patients showed the amelioration of auditory and speech function, and the restoration of sound source localization.

For binaural gene therapy, 3 × 10^12^ vg AAV1-hOTOF was injected into the inner ear, compared with the unilateral injection of 1.5 × 10^12^ vg (ref. ^11^). The operative time was extended and doubled during bilateral injection, compared with the unilateral injection. Also, the patients receiving binaural gene therapy were relatively younger (a median age of 2.8 years) than the patients receiving unilateral gene therapy (a median age of 4.1 years). These factors suggest that the patients receiving binaural gene therapy face potentially more risks. To reduce inflammatory response and potential infection risk, dexamethasone and ceftriaxone were administered intravenously. During the surgery, standard operational procedure was conducted, and after injection, professional nursing was provided. During the follow-up, no dose-limiting toxicity, ear or systemic infection, or serious AEs were observed. All 36 AEs were grade 1 or 2 (Table 3). Similar to the unilateral gene therapy, the IFN-γ ELISpot responses to the AAV1 capsid peptide pools and vector DNA in the blood were negative during the bilateral follow-up (Extended Data Fig. 5 and Extended Data Table 3). The titer of neutralizing antibodies in 5 patients with bilateral gene therapy at 6 weeks was 1:1,215, while the titer in 5 participants receiving a dose of 1.5 × 10^12^ vg for unilateral injection was 1:135–1:3,645 (3 patients with 1:135–1:405 neutralizing antibodies) at 6 weeks. The result suggests that the neutralizing antibodies were relatively higher in the bilateral injection group than in the unilateral injection group, which was expected owing to an increase in the viral load. The exact cause of fever in patient 2 was unknown. It might have been caused by influenza, as no other concomitant symptoms or abnormalities were observed, except mild cough and elevated lymphocyte counts. These results indicate that binaural gene therapy of AAV1-hOTOF was relatively safe in DFNB9 patients via one-time surgery.

Efficacy analysis showed binaural hearing amelioration in all five patients. Compared with >95 dB at baseline, the average ABR threshold in the right (left) ear was improved to 58 dB (58 dB) in patient 1 and 55 dB (50 dB) in patient 3 26 weeks after injection (Fig. 2a,c); the average ABR threshold in the right (left) ear was 75 dB (78 dB) in patient 4 and 63 dB (63 dB) in patient 5 at 13 weeks (Fig. 2d,e). The results indicate that the hearing improvement is comparable in both ears in patients 1, 3, 4 and 5. At 26 weeks, in patient 2, the right (left) ear showed an improvement of more than 20 dB (>10 dB) of the average ABR threshold (Fig. 2b). A possible leakage of the AAV1-hOTOF solution from the round window during or after injection may account for the modest hearing recovery in patient 2. Another reason for different responses to gene therapy among patients may be related to individual differences. After treatment, patient 2 responded to the sound, including dancing to the music, as shown in Supplementary Video 2. A better recovery of the ABR threshold at 0.25 kHz may partly contribute to his sensitive response to the sound in daily life.

The arithmetic mean for the average ABR thresholds of the 10 ears in 5 patients with binaural treatment was 69 dB at 13 weeks after injection, while the arithmetic mean for the average ABR thresholds of the 5 ears in 5 patients receiving a dose of 1.5 × 10^12^ vg for unilateral treatment was >64 dB at 13 weeks (ref. ^11^). The arithmetic mean for the average ASSR thresholds at 13 weeks was 60 dB for the patients receiving bilateral gene therapy and 67 dB for the unilateral patients administered with 1.5 × 10^12^ vg AAV1-hOTOF^11^.

The study further evaluated the additional benefits of bilateral ear treatment for DFNB9 patients in a noisy environment and sound source localization. It is known that bilateral hearing improves speech recognition in a noisy environment and is required for better music perception, sound source localization and higher life satisfaction^12,13^. To evaluate the patient’s ability of auditory and speech perception, we used appropriate questionnaires and observed that the MAIS or IT-MAIS, CAP or MUSS scores were improved in five patients (Table 2), suggesting the amelioration of auditory function and speech perception. The improvement of speech perception was also shown by tests and videos in patients (Extended Data Table 2 and Supplementary Videos 1–4). These results correlated with the reduction of ABR and ASSR thresholds (Fig. 2). Music information is a complex acoustic signal. In this study, patients 2 and 3 showed the ability to appreciate music at 13–15 weeks after AAV1-hOTOF gene therapy, suggested by their dance movements when listening to music (Supplementary Videos 2 and 3). Due to the young age and short follow-up, more detailed evaluation is needed during subsequent follow-up visits.

The ability to localize sound source, determining the position of a sound source in three dimensions, is important for speech communication and daily safety such as driving^20^. Patients had congenital hearing loss without the capability of sound source localization before treatment. After gene therapy, the ability of sound source localization was restored in all patients, indicated by the SSQ-P questionnaires, videos and tests (Table 2, Supplementary Videos 1 and 2, and Extended Data Table 2). In patient 2, the improvement of the average ABR threshold was >10 dB in the left ear and >20 dB in the right ear at 26 weeks; the average ASSR threshold showed an improvement of 19 dB (21 dB) in the right (left) ear at 26 weeks (Fig. 2b). Interestingly, patient 2 regained the capability of sound source localization, suggesting that even a modest hearing improvement in auditory function was sufficient to reconstitute the ability of sound source localization.

Binaural hearing recovery has been associated with better speech perception in the noise environment, the capability of sound source localization and higher life satisfaction and quality in patients^12,13,20^. Our results show that AAV1-hOTOF binaural gene therapy for patients with DFNB9 is feasible, safe and efficacious. The study expands the scope of DFNB9 treatment, potentially improving clinical intervention for hereditary deafness and promoting clinical transformation of gene therapy for hereditary deafness caused by other deafness genes. For children with congenital hearing loss, we recommend implementing universal genetic screening so that early intervention can be performed. In the future, investigation of gene therapy and cochlear implant in a larger randomized trial needs to be explored.

This trial is limited by the small patient numbers and the relatively short follow-up period. The trial is ongoing; long-term follow-up visit and more patients are needed for further investigation.

In conclusion, binaural AAV1-hOTOF gene therapy did not cause dose-limiting toxicity or serious AEs in five treated patients. Binaural AAV1-hOTOF gene therapy resulted in bilateral hearing restoration, the improvement of auditory and speech function, and the restoration of the ability of sound source localization in all treated patients.

## Methods

### Study design and patients

This single-arm, single-center trial was conducted at the Eye & ENT Hospital of Fudan University (Shanghai, China). Patients (1–18 years of age) with a confirmed genetic diagnosis of biallelic *OTOF* gene mutations and the average ABR thresholds ≥65 dB in both ears were eligible. Exclusion criteria included having a ratio of the titer of neutralizing antibodies to AAV1 > 1:2,000. Detailed inclusion and exclusion criteria are listed below.

#### Patient inclusion criteria

1. Participants or their legal guardians can fully understand and voluntarily sign the informed consent form of this study and are willing to cooperate with follow-up visits at the specified timepoints in the trial.
2. Participants are able to communicate well with the researchers and comply with the requirements with the help of guardians. Young children without mature language skills could cooperate and comply with the requirements with the help of guardians.
3. A proper understanding of the trial and an appropriate expectation of the benefits.
4. 1–18 years old; gender is not limited.
5. A diagnosis of DFNB9 congenital deafness was determined based on the clinical symptoms and gene mutation analysis for the presence of either *OTOF* homozygous or biallelic mutations in *OTOF*.
6. Audiological inclusion criterion: severe-to-complete hearing loss (≥65 dB).
7. Participants satisfy the requirements for otologic surgery. Participants with middle–inner ear deformity, vestibular–cochlear nerve development abnormality, ear inflammation and so on, determined through computed tomography (CT) and/or magnetic resonance imaging (MRI) within 3 months or during screening, are excluded.

#### Patient exclusion criteria

1. Gene analysis does not suggest any *OTOF* mutation or gene analysis suggests other concomitant gene mutations causing hearing loss.
2. Other types of deafness that are not suitable for otologic surgery, such as conductive deafness, mixed deafness, malformation syndrome caused by middle–inner ear dysplasia or malformation, and abnormalities of the vestibular nerve or cochlear nerve determined through CT or MRI scan within 3 months.
3. Preexisting otologic diseases that may interfere with the interpretation of study endpoints, such as acute–chronic otitis media, Meniere’s disease, acoustic neuroma or unrecovered sudden deafness.
4. A history of substance abuse, any ototoxic drug treatment (such as aminoglycosides, cisplatin or loop diuretics) within 6 months, antiviral therapy or immunotherapy within 3 months, or vaccination within 1 month.
5. A history of complex immunodeficiency or organ transplantation.
6. Patients with severe systemic disease or active bacterial or viral infection, such as pulmonary tuberculosis, active hepatitis B or C infection, active herpes zoster infection, pancreatitis, renal failure or gastrointestinal ulcers.
7. Patients with contraindications to surgery or anesthesia certified by the surgeon, anesthesiologist or designee, such as an allergy to the study medication and a cardiovascular or cerebrovascular accident that occurred within the past 6 months, including myocardial infarction, heart failure, angina pectoris, cerebrovascular accident or transient ischemic attack.
8. Currently participating in or planning to participate in another clinical trial involving a drug or device within 1 year, or within 5 half-lives after the last dosing in another clinical trial.
9. Bilateral ear implants (for example, cochlear implants).
10. With >1:2,000 neutralizing antibodies against the AAV1 capsid.
11. Other severe congenital diseases.
12. A clear history of neurological or psychiatric disorders, including epilepsy or dementia.
13. Patients who require long-term anticoagulants and cannot be interrupted in the short term.
14. A history of radiotherapy and chemotherapy.
15. Other conditions that investigators do not consider appropriate for participating in the present clinical study.

To promote safety, older children (aged ≧3 years) were enrolled first, followed by younger children. The patients were sequentially enrolled after evaluation of dose-limiting toxicity. Firstly, we conducted AAV1-hOTOF unilateral gene therapy including 1 patient receiving a dose of 9 × 10^11^ vg and 5 patients receiving a dose of 1.5 × 10^12^ vg. The results showed that AAV1-hOTOF unilateral gene therapy is safe and efficacious and has recently been published^11^. Subsequently, we expanded the study to bilateral gene therapy to provide additional benefits to patients, including better speech perception in the noise environment, the ability to localize the sound source and higher life satisfaction. We carried out the study after we amended the protocol that was approved by the ethics committee. Based on the safety and efficacy of the 1.5 × 10^12^ vg dosage in multiple patients in the study of unilateral gene therapy, we selected a dose of 1.5 × 10^12^ vg per ear for bilateral gene therapy. For the binaural gene therapy, the first patient was 11.0 years old; subsequently, the younger children were enrolled.

The trial was approved by the Ethics Committee of Eye & ENT Hospital of Fudan University and conducted in accordance with the principles of the Declaration of Helsinki. Written informed consent was obtained from parents or legal guardians of the children before enrollment. Before sharing videos of patients, consent was obtained again. A safety monitoring board was involved with the study.

### Protocol amendment

For unilateral gene therapy, the protocol was approved by the Ethics Committee of Eye & ENT Hospital of Fudan University on 24 June 2022. The trial was prospectively registered in September 2022. During the trial, the protocol was amended to make it more reasonable and operationally feasible, considering the clinical risks and benefits for participating subjects. We provided detailed protocol amendments here and in Supplementary Information.

#### Protocol amendments

1. The age of participants was expanded (from 3–10 years to 1–18 years).
2. The number of enrolled patients was expanded (from 2–3 cases to 4–12 cases), and more patients could be recruited into the 50 μl (1.5 × 10^12^ vg) group after confirming that dose-limiting toxicity occurred in ≤1/3 of the patients in this group.
3. Add an alternative exploratory dose group (70 μl).
4. Add the option of double injection (including bilateral injection).
5. For evaluation of speech, remove Sun Xibin’s method and add the Auditory Performance Rating Scale (CAP) and SIR.
6. Add additional indicators (that is, near-infrared light functional imaging, electroencephalogram, music test, and growth and development scales).
7. Adjust follow-up timepoints for otoscopy and vestibular function.
8. Add follow-up timepoints for blood collection.

Bilateral *OTOF* gene therapy would provide important benefits for DFNB9 patients. After confirming the safety and efficacy of unilateral gene therapy in DFNB9 patients^11^, we expanded the trial to bilateral administration. The revised protocol was approved by the Ethics Committee of Eye & ENT Hospital of Fudan University on 6 July 2023. For binaural gene therapy, the first patient was enrolled on 14 July 2023, and the last patient was enrolled on 15 November 2023.

### Endpoints

The dose-limiting toxicity at 6 weeks was the primary endpoint, defined as hematologic toxicity ≥ grade 4, nonhematologic toxicity ≥ grade 3 or aural toxicity ≥ grade 2 within 6 weeks. The grade was assessed according to the CTCAE V5.0. The secondary endpoint included safety and efficacy. Safety was measured using AEs after treatment. Routine blood tests, blood biochemistry, coagulation function and routine urine tests were evaluated at baseline, 3 days, 7 days, 2 weeks, 4 weeks, 6 weeks, 13 weeks and 26 weeks after gene therapy. CT and MRI were assessed at baseline and 6 weeks. Neutralizing antibodies and IFN-γ ELISpot assays were measured at baseline and 6 weeks. Vector DNA was measured at baseline and 7 days. Efficacy outcomes included auditory function and speech perception. ABR and ASSR were used to evaluate the auditory function in the patients. The average threshold of ABR or ASSR was defined as the arithmetic mean at 0.5, 1, 2 and 4 kHz (ref. ^21^). The SNR of DPOAE was also detected. To evaluate auditory function and speech perception, questionnaires were used, including MAIS^22^, IT-MAIS^22^, CAP^23^, SIR^24^ and MUSS^25^. Speech assessment software was also used to assess the speech perception, including Mandarin Speech Perception (version 5.04.01)^26^ and Angel Test (version 5.01.01)^27^. To assess the ability of sound source localization, SSQ-P questionnaires^28,29^ were used and a sound source localization test^30,31^ was performed. ABR, ASSR and DPOAE were performed at baseline, 4 weeks, 6 weeks, 13 weeks and 26 weeks after bilateral injection. Speech perception and sound source localization were assessed at baseline, 6 weeks, 13 weeks and 26 weeks.

### Clinical study treatment

Genotyping was conducted using whole exome sequencing and verified by three independent geneticists. Starting from 3 days before AAV1-hOTOF injection, patients received daily intravenous dexamethasone (0.3 mg kg^−1^) until 5 days after AAV1-hOTOF injection. Under general anesthesia, patients received AAV1-hOTOF bilaterally through the round window membrane with stapes fenestration at a dose of 1.5 × 10^12^ vg per ear in a volume of 50 μl. The injection was performed using an endoscope through the external auditory canal to minimize the trauma. The detailed surgical procedure is described in Supplementary Information. Starting on the day of AAV1-hOTOF injection, patients received daily intravenous ceftriaxone (80 mg kg^−1^) for 5 consecutive days, at a maximum dose of 2 g day^−1^.

### Production and delivery of AAV1-hOTOF

The AAV1-hOTOF, containing the functional human *OTOF* coding sequence packaged by dual-AAV vectors, was produced by PackGene Biotechnology and stored at ≤−65 °C. The detailed composition and structure of AAV1-hOTOF (patent application number 202311051611.4) have been described in our previous paper^10,11^. Briefly, the full-length human *OTOF* coding sequence (NM_001287489.2) was split into 5′ N-terminal and 3′ C-terminal segments between the exon 21 and exon 22 junction sites. AAV1-hOTOF included AAV1-hOTOF NT (5’ terminal segment of human *OTOF* coding sequence) and AAV1-hOTOF CT (3’ terminal segment of human *OTOF* coding sequence). Hair cell-specific promoter, *Myo15* promoter (patent number US 2021/0388045 A1), was used to drive the expression of the human *OTOF* coding sequence. The AAV1-hOTOF NT carried the *Myo15* promoter, the 5′ N-terminal segment of *OTOF* coding sequence, a splicing donor sequence and a recombinogenic sequence (AK) derived from F1 phage. The AAV1-hOTOF CT carried an AK sequence, a splicing acceptor sequence, the 3′ C-terminal segment of the *OTOF* coding sequence, a woodchuck hepatitis virus posttranscriptional regulatory element and a bovine growth hormone polyadenylation sequence. The full sequence is provided in Supplementary Information. The AAV1-hOTOF was injected into the inner ear via the round window membrane under an endoscope (7220AA, Karl Storz) through the external tympanic auditory canal route. The injection volume was 50 μl (1.5 × 10^12^ vg) per ear, and the injection speed was 120 nl s^−1^.

### Detection of anti-AAV1 neutralizing antibody

Blood samples were collected from the patients. At baseline and after surgery, the titer of anti-AAV1 neutralizing antibodies was determined. Cultured in complete medium containing DMEM (Gibco, 11995-065), 10% fetal bovine serum (Gibco, A5669701) and 1% penicillin–streptomycin (Gibco, 15140122), 1 × 10^4^ HEK-293FT cells per well were seeded into a 96-well plate and cultured for 24 h at 37 °C in a cell culture incubator. After gradient dilution, the patient’s serum (60 μl) was mixed with 60 μl AAV1-Luc Solution (Packgene Biotechnology) and incubated for 1 h at 37 °C. Then, the incubated blood sample (30 μl) was co-incubated with cells for 24 h at 37 °C. Next, the liquid was removed from the 96-well plate, luciferase detection reagent was added to the wells, and the plate was shaken at 400 rpm for 5–10 min at room temperature. Subsequently, the relative light unit (RLU) was measured using a microplate reader (MD, Spectra Max i3x). The titer of anti-AAV1 neutralizing antibodies was defined as the reciprocal of maximal dilution, at which over 50% inhibition of RLU was yielded relative to the negative control. Percentage inhibition was calculated using the following equation: inhibition (%) = (100 − ((sample RLU − cell control RLU)/(negative control RLU − cell control RLU)) × 100)%. Cell control is the HEK-293FT cells without treatment of AAV1-Luc Solution. Negative control is the negative serum without anti-AAV1 neutralizing antibody.

### IFN-γ ELISpot

To detect circulating T cell responses to the AAV1 capsid in blood, IFN-γ ELISpot assay was performed, according to our previous report^11^. At baseline and after injection of AAV1-hOTOF, a fresh whole blood sample was collected. Then, PBMCs were isolated using PBMC isolation buffer (TBD Science, HY2015 (LTS10770125)), washed twice in 50 ml 1× PBS and centrifuged for 10 min at 500 × *g*. Subsequently, PBMCs were resuspended in serum-free cryopreservation medium (NCM Biotech, C40100) and stored at −80 °C before analysis.

ELISpot assay was performed using an ELISpot PRO: Human IFN-γ (ALP) Kit (MABTECH, code: 3420-2AST-10). Precoated ELISpot strip plates (MABTECH, code: 3420-3SPT) were washed in 1× PBS four times. Then, the wells were blocked with 200 µl PBMC complete media (including RPMI medium, 10% fetal bovine serum and 1% penicillin–streptomycin) for 30 min at room temperature and washed in 1× PBS. Next, 100 µl PBMC resuspension was added to the well and incubated with 100 µl AAV1 mixed peptide pool solution (GL Biochem) for 24 h at 37 °C. After washing, 100 µl IFN-γ antibody (MABTECH, code: 3420-9A) was added to each well, and the plate was incubated at room temperature for 2 h in the dark. After the wells were washed in 1× PBS five times, 100 µl BCIP/NBT-plus substrate (MABTECH, code: 3650-10) was added to each well, and the reaction was incubated at room temperature for 30 min in the dark. Dark spots signaling the activated T cells were detected, and the reactions were terminated by washing the plate with 1× PBS. The number of spots forming units (SFUs) was calculated via an ELISpot Reader (AID iSpot). A positive result would be reported when the number of SFUs of the sample was over ten times the number of SFUs of the negative control. The negative control included PBMCs plus the medium alone. The positive control contained 100 µl of CTL-Test medium including 2 µg ml^−1^ anti-human CD3 antibody (MABTECH, code: 3605-1S), which could activate all T cells in a nonspecific manner.

### Detection of vector DNA

To detect the amount of vector DNA in blood, quantitative polymerase chain reaction (qPCR) was performed. At baseline and after injection, a whole blood sample was collected, and then 200 μl of blood sample was incubated with 220 μl of mixture including lysis buffer and protease K (Roche, 03115828001) at 56 °C for 10 min. The genomic DNA was isolated using a DNeasy Blood & Tissue Kit (QIAGEN, 69506) according to the manufacturer’s instructions. qPCRs were prepared with AceQ qPCR Probe Master Mix (Vazyme, Q112-02) and performed in LightCycler 480 Instrument II (Roche). The sequence for the reverse primer was GCAAAATCCCAGAAACGCAAGAG; the sequence for the forward primer was CTGAGGCTGTGCCAGAACT; the sequence for the probe was 5′-FAM-TCCTGGCGGACGAGGTAAGTATCAAGG-BHQ1-3′.

### ABR

The patients were anesthetized. In a double-walled soundproof room, the ABR thresholds were assessed at 0.25 kHz, 0.5 kHz, 1 kHz, 2 kHz and 4 kHz using the auditory evoked potential system (Bio-logic). Three electrodes (non-inverting, inverting and grounding electrodes) were placed at the high forehead, ipsilateral mastoid process and contralateral mastoid, respectively. The visually detectable wave V marked the presence of the auditory brainstem response waveform.

### ASSR

ASSR was performed in a double-walled soundproof room and measured using the auditory evoked potential system (Bio-logic), as previously described^11^. The hearing thresholds were assessed at five frequencies (0.25 kHz, 0.5 kHz, 1 kHz, 2 kHz and 4 kHz) using air conduction stimulation. The simulation was evoked at different intensities by changing the stimulus level in 5–10 dB steps between 20 dB and 120 dB. Electrode disks were fixed with electrolytic paste at Fz (positive), ipsilateral mastoid (negative) and Fpz (ground). Fpz is the nasion (bridge of the nose). Fz is the middle of the forehead. Impedance was no more than 5 kOhm in all electrodes. Amplifier gain was 100,000 with cutoff frequencies of 10 Hz and 300 Hz; the sample period was digitized with 1.37 ms. Each signal epoch was recorded for about 3 min, and approximately 20–24 epochs were averaged. ASSR values were detected to a 1% error margin (automatically with the detection algorithm).

### DPOAE

An AudX Plus OAE system (Madsen) was used to record DPOAE in a double-walled soundproof room. To elicit the DPOAEs, two pure tones, including *f*_1_ and *f*_2_ primary tones (*f*_2_/*f*_1_ = 1.22), were evoked simultaneously, with the lower-frequency primary tone at 65 dB and the higher-frequency primary tone at 55 dB. Five frequencies, including 0.5 kHz, 1 kHz, 2 kHz, 4 kHz and 8 kHz, were tested. The levels of 2*f*_1_–*f*_2_ DPOAE were recorded. The SNR was reported at each tested frequency. An SNR > 6 dB was defined as ‘present and normal’.

### Auditory and speech perception

Various types of questionnaires were used to assess auditory and speech perception, according to the auditory level and cognitive development in patients. Questionnaires included MAIS^22^, IT-MAIS^22^, CAP^23^, SIR^24^ and MUSS^25^. Speech assessment software including Mandarin Speech Perception (version 5.04.01)^26^ and Angel Test (version 5.01.01)^27^ was used. Speech perception tests included monosyllable, disyllable, sentence recognition, environmental sound test, final recognition test, initial recognition test and lexical tone test in a quiet environment.

### Sound source localization

Questionnaires were used to evaluate the ability of sound source localization in patients, including the SSQ-P^28,29^. SSQ-P was used to evaluate children’s ability of speech perception and spatial hearing. Sound source localization was also measured using I-CAST software (version 5.05.03)^30,31^ in the sound field. RMSE was used as the evaluation index of sound source localization accuracy.

### Statistical analysis

The sample size of the study was based on enrollment feasibility. The definition of hearing restoration is a 10 dB reduction in the average ABR threshold, according to the guidelines for sudden sensorineural hearing loss^32^. Regarding the statistical analysis plan, descriptive statistics included number of subjects, mean, median and s.d.; all analyses, including patient disposition, primary outcome, auditory function, speech perception, sound source localization and safety, were descriptively summarized. And analyses are performed on all enrolled patients. Audiometric and ELISpot figures were made using Graphpad Prism 8.

### Reporting summary

Further information on research design is available in the Nature Portfolio Reporting Summary linked to this article.

## Online content

Any methods, additional references, Nature Portfolio reporting summaries, source data, extended data, supplementary information, acknowledgements, peer review information; details of author contributions and competing interests; and statements of data and code availability are available at 10.1038/s41591-024-03023-5.

### Supplementary information

Supplementary Information AAV1-hOTOF Structure and Sequence, Supplementary Videos, Trial Protocol.

Reporting Summary

Supplementary Video 1 Patient 1, aged 11.0 years, was born deaf. It has been 11 years for her without her hearing. She could not respond to sound and recognize sound at baseline. After gene therapy, she could turn back when her name was called 4 weeks after injection. And she could recognize sound 6 weeks after injection. Remarkably, she could speak syllables at 13 weeks, such as ‘a’, ‘ba’ (father), ‘i’, ‘u’, ‘s’ and ‘ma’ (mother). She was able to complete the sound localization test well at 13 weeks.

Supplementary Video 2 Patient 2 could not respond to sound owing to deafness at baseline. After gene therapy, he was able to turn to his grandmother and grandfather when his name was called from the left and right of his backward side at 6 weeks. He could dance to music, complete some simple instructions at 15 weeks and say some simple words at 26 weeks (for example, ‘ayi’ (aunt) and ‘bai’ (bye)).

Supplementary Video 3 Patient 3 showed no response to sound at baseline. After gene therapy, he could turn back when his name was called 3 weeks after injection. At 13 weeks, he was able to move his body and dance when he heard music. At 26 weeks, he was able to say some simple words, for example, ‘baba’ (father), ‘nainai’ (grandmother) and ‘yeye’ (grandfather).

Supplementary Video 4 Patient 4 showed no response to sound at baseline. After gene therapy, she could turn back when her name was called at 4 weeks. She could complete some simple instructions at 13 weeks, and she could say simple words at 20 weeks, for example, ‘baba’ (father), ‘mama’ (mother) and ‘nainai’ (grandmother).

### Source data

Source Data Fig. 1 Fig. 2, Extended Data Fig. 2 and Extended Data Fig. 5.

## Figures and Tables

**Extended Data Table 1:**
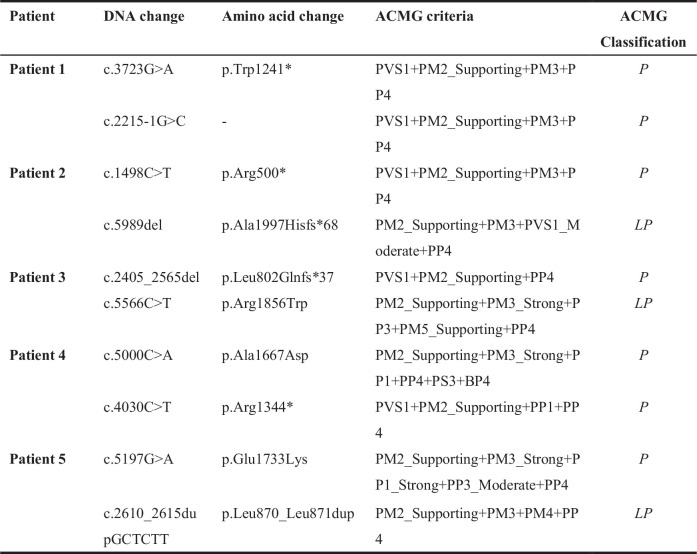
*OTOF* variant interpretation in patients

Transcript ID: NM_001287489.2. ACMG, the American College of Medical Genetics and Genomics; *P*, pathogenic; *LP*, likely pathogenic.

**Extended Data Table 2:**
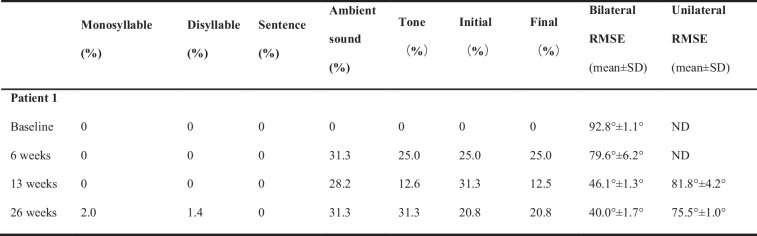
Speech perception and sound source localization in patient 1

RMSE: root mean square error. ND: not done. Patients 2, 3, 4 and 5 were unable to complete the above tests due to age limitation.

**Extended Data Table 3:**
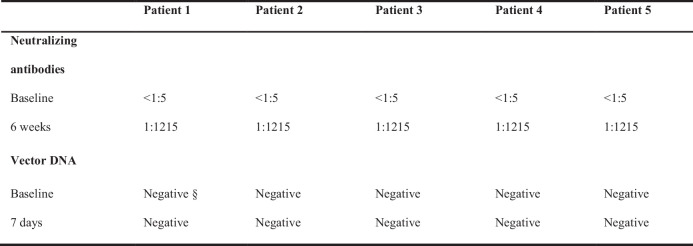
Immune responses and vector shedding

§ Negative indicates the amount of vector DNA is below the lower limit of detection.

## Data Availability

Individual de-identified participant data are available in the text, tables and figures of the Article. The detailed trial protocol including the statistical analysis plan is available in Supplementary Information. Requests for more information on the trial should be directed to corresponding author Y.S. and will be responded to within 120 days. Source data are provided with this paper.
