# Supplementary material for: Bilateral gene therapy in children with autosomal recessive deafness 9: single-arm trial results
Source: Nat Med. 2024 Jun 5;30(7):1898–904. doi: 10.1038/s41591-024-03023-5 (PMC11271389; doi:10.1038/s41591-024-03023-5)
Supplement: Supplementary file 1 — AAV1-hOTOF Structure and Sequence, Supplementary Videos, Trial Protocol. [file 41591_2024_3023_MOESM1_ESM.pdf]

nature medicine

Supplementary information https://doi.org/10.1038/s41591-024-03023-5

Bilateral gene therapy in children with autosomal recessive deafness 9: single-arm trial results

In the format provided by the authors and unedited

SUPPLEMENTARY NOTE

AAV1-hOTOF structure and sequence

Supplementary Videos | Behavioral audiometry and speech communication

Trial Protocol

AAV1-hOTOF structure and sequence

AAV1-hOTOF included AAV1-hOTOF NT (5’ terminal segment of human *OTOF* coding sequence) and AAV1-hOTOF CT (3’ terminal segment of human *OTOF* coding sequence).

The sequence of AAV1-hOTOF NT:

GCGGCCGCACGCGTGGAGCTAGCCCATATATGGAGTTCCGCTGCAGCTCAGCCTACT
ACTTGCTTTCCAGGCTGTTCCTAGTTCCCATGTCAGCTGCTTGTGCTTTCCAGAGACAA
AACAGGAATAATAGATGTCATTAAATATACATTGGGCCCCAGGCGGTCAATGTGGCA
GCCTGAGCCTCCTTTCCATCTCTGTGGAGGCAGACATAGGACCCCCAACAAACAGCA
TGCAGGTTGGGAGCCAGCCACAGGACCCAGGTAAGGGGCCCTGGGTCCTTAAGCTTC
TGCCACTGGCTCCGGCATTGCAGAGAGAAGAGAAGGGGCGGCAGAGCTGAACCTTA
GCCTTGCCTTCCTGGGTACCCTTCTGAGCCTCACTGTCTTCTGTGAGATGGGCAAAGT
GCGGGTGTGACTCCTTGGCAACGGTGTTACACCAGGGCAGGTAAAGTTGTAGTTATTT
GTGGGGTACACCAGGACTGTTAAAGGTGTAACTATGGTCTCACCCAGCATTTTCACTT
CTAATAAGTTCAAATGTGATACGGCACCTTTCTAAAAATTAGTTTTCAGGGAAATAGG
GTTCAAAACTGGTAGTGGTAGGGTCCATTCTCACGACCCCCAGGCCTGCTAACCCTGA
CCAAGCTACCTATTACTTACCCTCCTCTTTCTCCTCCTCCTCTTTCTCCTTCTCCTGCTT
CCCCTCTTCCTTCTCCCTCCCTTCCTCTCCCTCCTCCCCCTCCTTGGCTGTGATCAGATC
CAGAGCCTGAATGAGCCTCCTGACCCCACACCCCCACTAGCATGGGCCTGCAAGTGC
CCAGAAGTCCCTCCTGCCTCCTAAACTGCCCAGCCGATCCATTAGCTCTTCCTTCTTCC
CAGTGAAAGAAGCAGGCACAGCCTGTCCCTCCCGTTCTACAGAAAGGAAGCTACAGC
ACAGGGAGGGCCAAAGGCCTTCCTGGGACTAGACAGTTGATCAACAGCAGGACTGG
AGAGCTGGGCTCCATTTTTGTTCCTTGGTGCCCTGCCCCTCCCCATGACCTGCAGAGA
CATTCAGCCTGCCAGGCTTTATGAGGTGGGAGCTGGGCTCTCCCTGATGTATTATTCA
GCTCCCTGGAGTTGGCCAGCTCCTGTTACACTGGCCACAGCCCTGGGCATCCGCTTCT
CACTTCTAGTTTCCCCTCCAAGGTAATGTGGTGGGTCATGATCATTCTATCCTGGCTTC
AGGGACCTGACTCCACTTTGGGGCCATTCGAGGGGTCTAGGGTAGATGATGTCCCCCT
GTGGGGATTAATGTCCTGCTCTGTAAAACTGAGCTAGCTGAGATCCAGGAGGGCTTG
GCCAGAGACAGCAAGTTGTTGCCATGGTGACTTTAAAGCCAGGTTGCTGCCCCAGCA
CAGGCCTCCCAGTCTACCCTCACTAGAAAACAACACCCAGGCACTTTCCACCACCTCT
CAAAGGTGAAACCCAAGGCTGGTCTAGAGAATGAATTATGGATCCTCGCTGTCCGTG
CCACCCAGCTAGTCCCAGCGGCTCAGACACTGAGGAGAGACTGTAGGTTCAGCTACA
AGCAAAAAGACCTAGCTGGTCTCCAAGCAGTGTCTCCAAGTCCCTGAACCTGTGACA
CCTGCCCCAGGCATCATCAGGCACAGAGGGCCACCTTGGGATTCGAACATCGATTGA
ATTCCCCGGGGATCCTCTAGGCCACCATGGCCTTGCTCATCCACCTCAAGACAGTCTC
GGAGCTGCGGGGCAGGGGCGACCGGATCGCCAAAGTGACTTTCCGAGGGCAATCCTT
CTACTCTCGGGTCCTGGAGAACTGTGAGGATGTGGCTGACTTTGATGAGACATTTCGG

TGGCCGGTGGCCAGCAGCATCGACAGAAATGAGATGCTGGAGATTCAGGTTTTCAAC
TACAGCAAAGTCTTCAGCAACAAGCTCATCGGGACCTTCCGCATGGTGCTGCAGAAG
GTGGTAGAGGAGAGCCATGTGGAGGTGACTGACACGCTGATTGATGACAACAATGCT
ATCATCAAGACCAGCCTGTGCGTGGAGGTCCGGTATCAGGCCACTGACGGCACAGTG
GGCTCCTGGGACGATGGGGACTTCCTGGGAGATGAGTCTCTTCAAGAGGAAGAGAAG
GACAGCCAAGAGACGGATGGACTGCTCCCAGGCTCCCGGCCCAGCTCCCGGCCCCCA
GGAGAGAAGAGCTTCCGGAGAGCCGGGAGGAGCGTGTTCTCCGCCATGAAGCTCGGC
AAAAACCGGTCTCACAAGGAGGAGCCCCAAAGACCAGATGAACCGGCGGTGCTGGA
GATGGAAGACCTTGACCATCTGGCCATTCGGCTAGGAGATGGACTGGATCCCGACTC
GGTGTCTCTAGCCTCAGTCACAGCTCTCACCACTAATGTCTCCAACAAGCGATCTAAG
CCAGACATTAAGATGGAGCCAAGTGCTGGGCGGCCCATGGATTACCAGGTCAGCATC
ACGGTGATCGAGGCCCGGCAGCTGGTGGGCTTGAACATGGACCCTGTGGTGTGCGTG
GAGGTGGGTGACGACAAGAAGTACACATCCATGAAGGAGTCCACTAACTGCCCCTAT
TACAACGAGTACTTCGTCTTCGACTTCCATGTCTCTCCGGATGTCATGTTTGACAAGA
TCATCAAGATTTCGGTGATTCACTCCAAGAACCTGCTGCGCAGTGGCACCCTGGTGGG
CTCCTTCAAAATGGACGTGGGAACCGTGTACTCGCAGCCAGAGCACCAGTTCCATCA
CAAGTGGGCCATCCTGTCTGACCCCGATGACATCTCCTCGGGGCTGAAGGGCTACGT
GAAGTGTGACGTTGCCGTGGTGGGCAAAGGGGACAACATCAAGACGCCCCACAAGG
CCAATGAGACCGACGAAGATGACATTGAGGGGAACTTGCTGCTCCCCGAGGGGGTGC
CCCCCGAACGCCAGTGGGCCCGGTTCTATGTGAAAATTTACCGAGCAGAGGGGCTGC
CCCGTATGAACACAAGCCTCATGGCCAATGTAAAGAAGGCTTTCATCGGTGAAAACA
AGGACCTCGTGGACCCCTACGTGCAAGTCTTCTTTGCTGGCCAGAAGGGCAAGACTTC
AGTGCAGAAGAGCAGCTATGAGCCCCTGTGGAATGAGCAGGTCGTCTTTACAGACCT
CTTCCCCCCACTCTGCAAACGCATGAAGGTGCAGATCCGAGACTCGGACAAGGTCAA
CGACGTGGCCATCGGCACCCACTTCATTGACCTGCGCAAGATTTCTAATGACGGAGA
CAAAGGCTTCCTGCCCACACTGGGCCCAGCCTGGGTGAACATGTACGGCTCCACACG
TAACTACACGCTGCTGGATGAGCATCAGGACCTGAACGAGGGCCTGGGGGAGGGTGT
GTCCTTCCGGGCCCGGCTCCTGCTGGGCCTGGCTGTGGAGATCGTAGACACCTCCAAC
CCTGAGCTCACCAGCTCCACAGAGGTGCAGGTGGAGCAGGCCACGCCCATCTCGGAG
AGCTGTGCAGGTAAAATGGAAGAATTCTTTCTCTTTGGAGCCTTCCTGGAGGCCTCAA
TGATCGACCGGAGAAACGGAGACAAGCCCATCACCTTTGAGGTCACCATAGGCAACT
ATGGGAACGAAGTTGATGGCCTGTCCCGGCCCCAGCGGCCTCGGCCCCGGAAGGAGC
CGGGGGATGAGGAAGAAGTAGACCTGATTCAGAACGCAAGTGATGACGAGGCCGGT
GATGCCGGGGACCTGGCCTCAGTCTCCTCCACTCCACCAATGCGGCCCCAGGTCACCG
ACAGGAACTACTTCCATCTGCCCTACCTGGAGCGAAAGCCCTGCATCTACATCAAGA
GCTGGTGGCCGGACCAGCGCCGCCGCCTCTACAATGCCAACATCATGGACCACATTG
CCGACAAGCTGGAAGAAGGCCTGAACGACATACAGGAGATGATCAAAACGGAGAAG
TCCTACCCTGAGCGTCGCCTGCGGGGCGTCCTGGAGGAGCTGAGCTGTGGCTGCTGCC

GCTTCCTCTCCCTCGCTGACAAGGACCAGGGCCACTCATCCCGCACCAGGCTTGACCG
GGAGCGCCTCAAGTCCTGCATGAGGGAGCTGGAAAACATGGGGCAGCAGGCCAGGA
TGCTGCGGGCCCAGGTGAAGCGGCACACGGTGCGGGACAAGCTGAGGCTGTGCCAG
AACTTCCTGCAGAAGCTGCGCTTCCTGGCGGACGAGGTAAGTATCAAGGTTACAAGA
CAGGTTTAAGGAGACCAATAGAAACTGGGCTTGTCGAGACAGAGAAGACTCTTGCGT
TTCTGGGATTTTGCCGATTTCGGCCTATTGGTTAAAAAATGAGCTGATTTAACAAAAA
TTTAACGCGAATTTTAACAAAATCTGATTTTGTAGGTAACCACGTGCGGACCGAGCGG
CCGC

The sequence of AAV1-hOTOF CT:
GCGGCCGCACGCGTGGAGCTAGCCCATATATGGAGTTCCGGGGATTTTGCCGATTTCG
GCCTATTGGTTAAAAAATGAGCTGATTTAACAAAAATTTAACGCGAATTTTAACAAA
ATGATAGGCACCTATTGGTCTTACTGACATCCACTTTGCCTTTCTCTCCACAGCCCCAG
CACAGCATTCCCGACATCTTCATCTGGATGATGAGCAACAACAAGCGTGTCGCCTATG
CCCGTGTGCCCTCCAAGGACCTGCTCTTCTCCATCGTGGAGGAGGAGACTGGCAAGG
ACTGCGCCAAGGTCAAGACGCTCTTCCTTAAGCTGCCAGGGAAGCGGGGCTTCGGCT
CGGCAGGCTGGACAGTGCAGGCCAAGGTGGAGCTGTACCTGTGGCTGGGCCTCAGCA
AACAGCGCAAGGAGTTCCTGTGCGGCCTGCCCTGTGGCTTCCAGGAGGTCAAGGCAG
CCCAGGGCCTGGGCCTGCATGCCTTCCCACCCGTCAGCCTGGTCTACACCAAGAAGC
AGGCGTTCCAGCTCCGAGCGCACATGTACCAGGCCCGCAGCCTCTTTGCCGCCGACA
GCAGCGGACTCTCAGACCCCTTTGCCCGCGTCTTCTTCATCAATCAGAGTCAGTGCAC
AGAGGTGCTGAATGAGACCCTGTGTCCCACCTGGGACCAGATGCTGGTGTTCGACAA
CCTGGAGCTCTATGGTGAAGCTCATGAGCTGAGGGACGATCCGCCCATCATTGTCATT
GAAATCTATGACCAGGATTCCATGGGCAAAGCTGACTTCATGGGCCGGACCTTCGCC
AAACCCCTGGTGAAGATGGCAGACGAGGCGTACTGCCCACCCCGCTTCCCACCTCAG
CTCGAGTACTACCAGATCTACCGTGGCAACGCCACAGCTGGAGACCTGCTGGCGGCC
TTCGAGCTGCTGCAGATTGGACCAGCAGGGAAGGCTGACCTGCCCCCCATCAATGGC
CCGGTGGACGTGGACCGAGGTCCCATCATGCCCGTGCCCATGGGCATCCGGCCCGTG
CTCAGCAAGTACCGAGTGGAGGTGCTGTTCTGGGGCCTACGGGACCTAAAGCGGGTG
AACCTGGCCCAGGTGGACCGGCCACGGGTGGACATCGAGTGTGCAGGGAAGGGGGT
GCAGTCGTCCCTGATCCACAATTATAAGAAGAACCCCAACTTCAACACCCTCGTCAA
GTGGTTTGAAGTGGACCTCCCAGAGAACGAGCTGCTGCACCCGCCCTTGAACATCCG
TGTGGTGGACTGCCGGGCCTTCGGTCGCTACACACTGGTGGGCTCCCATGCCGTCAGC
TCCCTGCGACGCTTCATCTACCGGCCCCCAGACCGCTCGGCCCCCAGCTGGAACACCA
CGGTCAGGCTTCTCCGGCGCTGCCGTGTGCTGTGCAATGGGGGCTCCTCCTCTCACTC
CACAGGGGAGGTTGTGGTGACTATGGAGCCAGAGGTACCCATCAAGAAACTGGAGA
CCATGGTGAAGCTGGACGCGACTTCTGAAGCTGTTGTCAAGGTGGATGTGGCTGAGG
AGGAGAAGGAGAAGAAGAAGAAGAAGAAGGGCACTGCGGAGGAGCCAGAGGAGGA

GGAGCCAGACGAGAGCATGCTGGACTGGTGGTCCAAGTACTTTGCCTCCATTGACAC
CATGAAGGAGCAACTTCGACAACAAGAGCCCTCTGGAATTGACTTGGAGGAGAAGG
AGGAAGTGGACAATACCGAGGGCCTGAAGGGGTCAATGAAGGGCAAGGAGAAGGCA
AGGGCTGCCAAAGAGGAGAAGAAGAAAACTCAGAGCTCTGGCTCTGGCCAGGG
GTCCGAGGCCCCCGAGAAGAAGAAACCCAAGATTGATGAGCTTAAGGTATACCCCAA
AGAGCTGGAGTCCGAGTTTGATAACTTTGAGGACTGGCTGCACACTTTCAACTTGCTT
CGGGGCAAGACCGGGGATGATGAGGATGGCTCCACCGAGGAGGAGCGCATTGTGGG
ACGCTTCAAGGGCTCCCTCTGCGTGTACAAAGTGCCACTCCCAGAGGACGTGTCCCG
GGAAGCCGGCTACGACTCCACCTACGGCATGTTCCAGGGCATCCCGAGCAATGACCC
CATCAATGTGCTGGTCCGAGTCTATGTGGTCCGGGCCACGGACCTGCACCCTGCTGAC
ATCAACGGCAAAGCTGACCCCTACATCGCCATCCGGCTAGGCAAGACTGACATCCGC
GACAAGGAGAACTACATCTCCAAGCAGCTCAACCCTGTCTTTGGGAAGTCCTTTGAC
ATCGAGGCCTCCTTCCCCATGGAATCCATGCTGACGGTGGCTGTGTATGACTGGGACC
TGGTGGGCACTGATGACCTCATTGGGGAAACCAAGATCGACCTGGAGAACCGCTTCT
ACAGCAAGCACCGCGCCACCTGCGGCATCGCCCAGACCTACTCCACACATGGCTACA
ATATCTGGCGGGACCCCATGAAGCCCAGCCAGATCCTGACCCGCCTCTGCAAAGACG
GCAAAGTGGACGGCCCCCACTTTGGGCCCCCTGGGAGAGTGAAGGTGGCCAACCGCG
TCTTCACTGGGCCCTCTGAGATTGAGGACGAGAACGGTCAGAGGAAGCCCACAGACG
AGCATGTGGCGCTGTTGGCCCTGAGGCACTGGGAGGACATCCCCCGCGCAGGCTGCC
GCCTGGTGCCAGAGCATGTGGAGACGAGGCCGCTGCTCAACCCCGACAAGCCGGGCA
TCGAGCAGGGCCGCCTGGAGCTGTGGGTGGACATGTTCCCCATGGACATGCCAGCCC
CTGGGACGCCTCTGGACATCTCACCTCGGAAGCCCAAGAAGTACGAGCTGCGGGTCA
TCATCTGGAACACAGATGAGGTGGTCTTGGAGGACGACGACTTCTTCACAGGGGAGA
AGTCCAGTGACATCTTCGTGAGGGGGTGGCTGAAGGGCCAGCAGGAGGACAAGCAG
GACACAGACGTCCACTACCACTCCCTCACTGGCGAGGGCAACTTCAACTGGCGCTAC
CTGTTCCCCTTCGACTACCTGGCGGCGGAGGAGAAGATCGTCATCTCCAAGAAGGAG
TCCATGTTCTCCTGGGACGAGACCGAGTACAAGATCCCCGCGCGGCTCACCCTGCAG
ATCTGGGATGCGGACCACTTCTCCGCTGACGACTTCCTGGGGGCCATCGAGCTGGACC
TGAACCGGTTCCCGCGGGGCGCAAAGACAGCCAAGCAGTGCACCATGGAGATGGCC
ACCGGGGAGGTGGACGTGCCCCTCGTGTCCATCTTCAAGCAAAAGCGCGTCAAAGGC
TGGTGGCCCCTCCTGGCCCGCAATGAGAACGATGAGTTTGAGCTCACGGGCAAGGTG
GAGGCTGAGCTGCATTTACTGACAGCAGAGGAGGCAGAGAAGAACCCAGTGGGCCT
GGCCCGCAATGAACCTGACCCCCTAGAGAAACCCAACCGGCCCGACACGGCCTTCGT
CTGGTTCCTCAACCCTCTCAAGTCCATCAAGTACCTCATCTGCACCCGGTACAAGTGG
CTCATCATCAAGATCGTGCTGGCGCTGTTGGGGCTGCTCATGTTGGGGCTCTTCCTCT
ACAGCCTCCCTGGCTACATGGTCAAAAAGCTCCTTGGGGCATGAAAGCTTGCCTCGA
GCCATTCCCGATAATCAACCTCTGGATTACAAAATTTGTGAAAGATTGACTGGTATTC
TTAACTATGTTGCTCCTTTTACGCTATGTGGATACGCTGCTTTAATGCCTTTGTATCAT

GCTATTGCTTCCCGTATGGCTTTCATTTTCTCCTCCTTGTATAAATCCTGGTTGCTGTCT
CTTTATGAGGAGTTGTGGCCCGTTGTCAGGCAACGTGGCGTGGTGTGCACTGTGTTTG
CTGACGCAACCCCCACTGGTTGGGGCATTGCCACCACCTGTCAGCTCCTTTCCGGGAC
TTTCGCTTTCCCCCTCCCTATTGCCACGGCGGAACTCATCGCCGCCTGCCTTGCCCGCT
GCTGGACAGGGGCTCGGCTGTTGGGCACTGACAATTCCGTGGTGTTGTCGGGGAAGC
TGACGTCCTTTCCATGGCTGCTCGCCTGTGTTGCCACCTGGATTCTGCGCGGGACGTC
CTTCTGCTACGTCCCTTCGGCCCTCAATCCAGCGGACCTTCCTTCCCGCGGCCTGCTGC
CGGCTCTGCGGCCTCTTCCGCGTCTTCGCCTTCGCCCTCAGACGAGTCGGATCTCCCTT
TGGGCCGCCTCCCCGCATCGGGGACTGTGCCTTCTAGTTGCCAGCCATCTGTTGTTTG
CCCCTCCCCCGTGCCTTCCTTGACCCTGGAAGGTGCCACTCCCACTGTCCTTTCCTAAT
AAAATGAGGAAATTGCATCGCATTGTCTGAGTAGGTGTCATTCTATTCTGGGGGGTGG
GGTGGGGCAGGACAGCAAGGGGGAGGATTGGGAAGACAATAGCAGGCATGCTGGGG
ATGCGGTGGGCTCTATGGCTGATTTTGTAGGTAACCACGTGCGGACCGAGCGGCCGC

Supplementary Videos | Behavioral audiometry and speech communication

To prepare and show the videos, we obtained informed consent from the patient's guardians. To protect the privacy of patients and their family members, we covered the patient's eyes and muted the patient's full name.

Supplementary Video 1: Patient #1, aged 11.0 years, is born deaf. It has been 11 years for her without hearing. She could not respond to sound and recognize sound at baseline. Excitingly, after gene therapy, she could turn back when her name was called 4 weeks after injection. And she could recognize sound 6 weeks after injection. Remarkably, she could speak syllables at 13 weeks, such as "a, Ba (Father), i, u, s, Ma (Mother)". She was able to complete the sound localization test well at 13 weeks.

Supplementary Video 2: Patient #2 could not response to sound due to deafness at baseline. After gene therapy, he was able to turn to his grandmother and grandfather when his name was called from the left/right of his backward side at 6 weeks. He could dance to music and complete some simple instructions at 15 weeks and say some simple words at 26 weeks (e.g., Ayi (Aunt) and Bai (Bye)).

Supplementary Video 3: Patient #3 showed no response to sound at baseline. After gene therapy, he could turn back when his name was called 3 weeks after injection. At 13 weeks, he was able to move his body and dance when he heard the music. At 26 weeks, he was able to say some simple words, e.g., Baba (Father), Nainai (Grandmother) and Yeye (Grandfather).

Supplementary Video 4: Patient #4 showed no response to sound at baseline. After gene therapy, she could turn back when her name was called at 4 weeks. She could complete some simple instructions at 13 weeks; and she could say simple words at 20 weeks, e.g., Baba (Father), Mama (Mother) and Nainai (Grandmother).

Trial Protocol

This supplement contains the following items:

1. The original protocol, and the final protocol, a summary of protocol amendments.

2. The original statistical analysis plan (Protocol v2; Section 15) and the final statistical analysis plan (Protocol v5; Section 15).

The safety, tolerability, and preliminary efficacy of RRG-003 AAV in the treatment of DFNB9 congenital deafness

Principal Investigator: Shu Yilai, Li Huawei

Sponsor: Eye & ENT Hospital of Fudan University

Protocol version number: 02, June 24, 2022

Signature

Date: June 24, 2022

Contents

1. Background ... 6

2. Research purpose ... 8

2.1 Primary purpose ... 8

2.2 Secondary purpose ... 8

3. Study design ... 8

4. Subject inclusion criteria, exclusion criteria, rejection criteria and suspension criteria ... 11

4.1 Inclusion Criteria ... 11

4.2 Exclusion Criteria ... 11

4.3 Suspension criteria ... 12

5. The number of cases required to achieve the intended purpose of the study based on statistical principles ... 13

6. The dosage form, dose, route of administration, method of administration, frequency of administration, course of treatment, and related regulations for combined medications, as well as instructions for packaging and labeling ... 13

6.1 General information ... 13

6.2 Administration Proposal ... 14

6.3 Surgical steps ... 15

7. Items of clinical and laboratory examinations to be performed, examination times, the amount of blood samples or tissues collected each time, sample collection times, and the total amount of samples to be collected ... 16

7.1 Observation indicators ... 16

7.2 Tolerability and safety observation indicators ... 16

7.3 Preliminary curative effect observation indicators ... 17

7.4 Biological sample collection, processing and testing ... 17

8. Registration and usage records, delivery and distribution methods and storage conditions of investigational drugs ... 17

9. Clinical studies, follow-up and measures to ensure subject compliance ... 18

10. Criteria for suspending the clinical study, regulations for ending the clinical study 18

11. Efficacy assessment criteria, including methods for evaluating parameters, observation time, recording and analysis ... 19

12. The storage procedure of subject's code, random number table, and case report form 19

13. The recording requirement of adverse event (AE) and the reporting, handling, follow-up visit, timing and outcome of serious AE 20

13.1 Definition 20
13.2 Severity of adverse event 21
13.3 Judgement of the causal relationship between adverse event and the investigational drug 22
13.4 Handling of adverse event 23
13.5 Recording of adverse event 23
13.6 Reporting of serious adverse event 24
13.7 Trail and follow-up visit of adverse event 25
13.8 Expected adverse reaction and handling 25

14. The establishment and maintenance of investigational drug code, the method of unblinding, and the rule of unblinding in the event of an emergency 29

15. Statistical analysis plan, definition, and selection of statistical analysis data set 29

15.1 Definition and selection of analysis data set 29
15.2 Statistical analysis plan 30

16. Data management and information Confidentiality 31

17. Quality control and quality assurance of clinical trial 31

17.1 Quality control (QC) 31

17.2 Quality assurance (QA) 32

18. Research relevant ethics 35

18.1 Examination and approval of trial documents from Ethics Committee before starting the trial 35
18.2 Subjects undergo the process of informed consent and acquire informed consent before starting the trial 36
18.3 Any AE that occurred during the trial can be effectively managed and followed up .. 36

19. Subject recruitment method and the process of acquiring informed consent 36

21. Follow-up and medical care after completing the trial 37

22. Responsibilities of the parties and other relevant regulation 37

List of Abbreviations

ABR	Auditory Brainstem Response
ADL	Activities of Daily Living
AAV	Adeno-associated Virus
ADR	Adverse Drug Reaction
AE	Adverse Event
ASSR	Auditory Steady State Response
AN	Auditory Neuropathy
ANSD	Auditory Neuropathy Spectrum Disorder
DFNB9	Autosomal Recessive Deafness 9
DLT	Dose-Limiting Toxicity
DPOAE	Distortion Product Otoacoustic Emission
EEAS	Efficacy Evaluable Analysis Set
EPS	Enrolled Patients Set
FDA	Food and Drug Administration
FAS	Full Analysis Set
GCP	Good Clinical Practice
IT-MAIS	Infant Toddler Meaningful Auditory Integration Scale
ITT	Intentionality Analysis
MSP	Mandarin Speech Perception
MAIS	Meaningful Auditory Integration Scale
PPS	Per-Protocol Set
PTA	Pure Tone Audiometry
QA	Quality Assurance
QC	Quality Control
SAS	Safety Analysis Set
SAE	Serious Adverse Event
SMP	Standard Management Procedure

SOP	Standard Operating Procedure
TEAE	Treatment-Emergent Adverse Event
UV	Ultraviolet
UADR	Unexpected Adverse Drug Reaction
WHO	World Health Organization

1. Background

Hearing loss is a common disease due to abnormalities in the auditory system. According to the World Health Organization (WHO), nearly 5% (about 466 million) of the population worldwide suffered from the disabling hearing loss, including 432 million adults and 34 million children, moreover, about one-third of elderly people over 65 own the disabling hearing loss as well. One billion people may be affected by the disease up to the middle of the century if hearing loss is not treated in time.[1] China is the country with the largest number of patients with hearing loss and 30,000 newborn infants are affected by this diease every year. Hereditary hearing loss accounts for about 60% of all hearing loss, additionally, there is no any medications used for the treatment of this disease. Auditory neuropathy (AN), also known as auditory neuropathy spectrum disorder (ANSD), it is one of the common otologic diseases that cause hearing loss, the prevalence of which is about 8.44% in children with profound hearing loss. Mutations to the *OTOF* gene (encoding the otoferlin), which causes autosomal recessive deafness 9 (DFNB9), are among the most common reasons for auditory neuropathy. The prevalence of *OTOF* is up to 41.2% in Chinese infants with AN.[2] However, there are no available therapeutic drugs for the treatment of the disease yet. Reconstruction of the auditory function by gene therapy is considered to be one of the most promising strategies to thoroughly treat hereditary deafness. Gene therapy refers to the delivery exogenous target gene to the patient with deafness by using delivery vector correct or compensate the abnormal genes, achieving a treatment of deafness.

The *OTOF* gene is recognized as among the most promising target in the field of gene therapy for deafness, which has great potential for clinical translation. *OTOF* mutation belongs to autosomal recessive inheritance, and can therefore be corrected by compensation of normal proteins. For the *Otof*$^{-/-}$ model mice, in recent years both groups in Germany and America have used dual AAV to deliver normal murine-derived Otof into the hair cells of mice, which significantly improved hearing of the model for at least 20 weeks.[3,4] The Akouos and Decibel companies in America firstly made the clinical protocols for the *OTOF* gene therapy system, which was approved by the FDA and was scheduled to be applied in patients in 2022. The highest prevalence of deafness in Chinese, there are no any clinical investigations for gene therapy of deafness yet, so it is of great public health importance to accelerate the development of drugs for treatment of deafness.

Our group is invariably committed to treating deafness by gene therapy and has developed an

effective and safe *OTOF* gene therapy system for DFNB9 mouse models. The therapy system used the adeno-associated virus vector (AAV) to deliver the normal human-derived *OTOF* gene into the inner ear of *Otof*$^{-/-}$ mice and significantly improved their auditory function. In addition, the *OTOF* gene therapy system was delivered into the inner ear followed by a comprehensive evaluation of its efficacy and safety through comparing different routes of administration, testing the hearing phenotypes of the animal, and assessing the toxicological risks of the drug. Based on our previous studies, the present project intends to conduct an investigator-initiated clinical trial to obtain a clinically available *OTOF* gene therapy drug, which will fill the domestic gap of clinical translation of gene therapy drugs for genetic deafness in China and will enable DFNB9 patients to obtain benefits.

The *OTOF* gene contains 48 exons and encodes the otoferlin which consists of 1997 amino acids, which mainly expresses in inner hair cells. The length of the *OTOF* sequence is approximately 6 kb, which together with the gene regulatory elements is longer than 7.5 kb, so it is difficult to package *OTOF* into a single AAV (~4.7 kb). Therefore, the present project uses dual AAV strategies to achieve in vivo high-efficient recombination of the *OTOF* sequence and to express functional otoferlin; also mouse hair cells will be high-efficiently and safely repaired by the inner ear hair cell-specific promoter, thereby expecting to restore auditory function of patients. Compared to previous reports, we intended to use human-derived *OTOF* and a specific-targeted promoter, which is characterized by higher safety and better tissue specificity.

AAV is the most commonly used delivery vector for gene therapy. Currently, three AAV-based drugs have been listed, including Glybera, Luxturna, and Zolgensma for the treatment of lipoprotein esterase deficiency, Leber congenital amaurosis, and spinal muscular atrophy, respectively. In addition, there are hundreds of AAV-related gene therapy drugs in the development of clinical pipelines. In the present research, AAV1 was used as the delivery vector due to its high usage frequency and proven manufacturing process. Glybera, the first listed gene therapy drug, uses AAV1 as its delivery vector. The pipelines developed by the AAV1 vector have been applied in various diseases, including muscular dystrophy, liver disease, hemophilia, viral infections, heart failure, and Huntington's chorea. Also, AAV1 is able to efficiently infect the inner ear hair cells of the target tissue in this project. The segmental package of the target gene using dual AAV vectors is a solution to break the upper limit of single vector packaging.

2. Research purpose

2.1 Primary purpose

To evaluate the safety and tolerability of RRG-003 injection in children with DFNB9 congenital hearing loss.

2.2 Secondary purpose

To evaluate the efficacy of escalating dose injections of RRG-003 in children with DFNB9 congenital hearing loss.

3. Study design

Study type: Interventional

Allocation and masking: non-randomized, open label

Intervention model: single arm, sequential assignment

This is a single-arm, open-label, sequential-assignment, non-randomized trial with dose ascending exploration. Considering the limited number of patients with rare diseases of Otoferlin mutations, and minimizing exposure to the risk of ineffective or low effective dose, the study design of investigational drug is as the following doses escalating protocol:

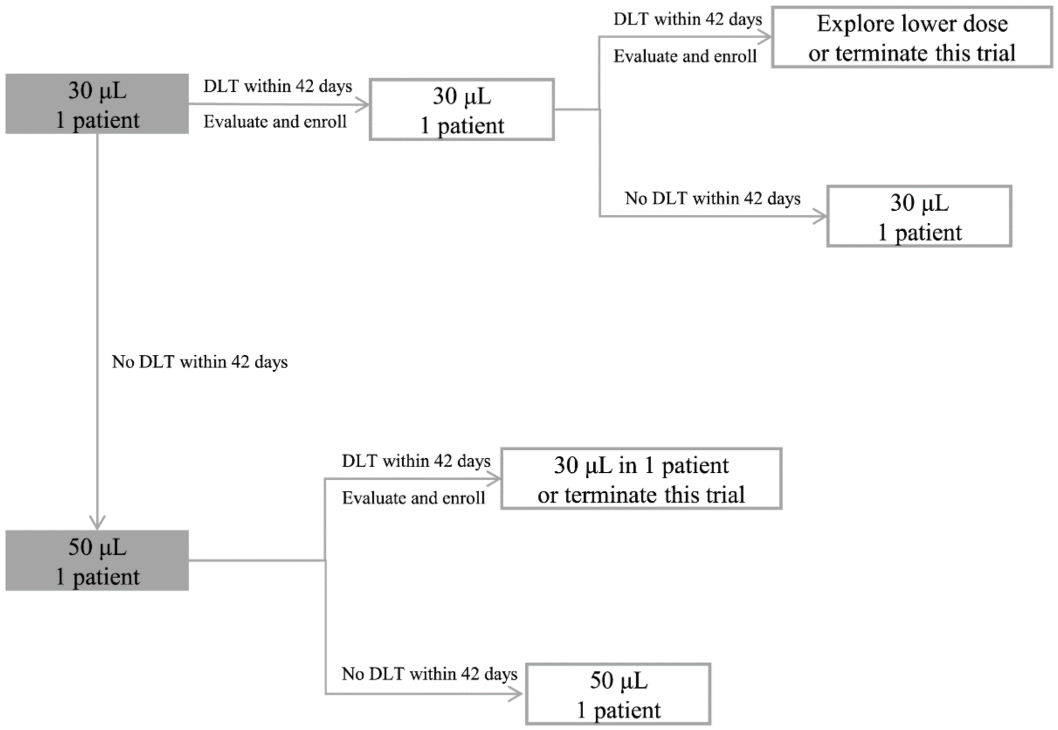

For participants who satisfy the inclusion criteria will be enrolled into this trial with the drug

concentration from low dose to high dose. To begin with, equally mixed AAV-OTOF-N and AAV-OTOF-C drug administrate through ear canal to infuse cochlea with a micropump.

The first participant is enrolled into the dose group of 30 μL (9E11 vg/ear) with single-dose administration. If dose limiting toxicity (DLT) is not observed within 42 days after administration, the next participant can be enrolled into the dose group of 50 μL (1.5E12 vg/ear). If DLT is observed in the first participant during the 42-day DLT evaluation period, the second participant should be enrolled in the dose group of 30 μL after risk evaluation by the investigators. If DLT is not observed in the second participant during the 42-day DLT evaluation period, the third participant can be enrolled in 30 μL group after risk evaluation by the investigators. If DLTs are observed in the second participant within 42 days, the investigator can enroll one participant into the lower dose group or terminate the study after careful evaluation.

The next dosage group is 50 μL (1.5E12 vg/ear). After the first participant receives a single-dose administration, the second participant can be enrolled into this dose group if there is no DLT observed during the 42-day DLT evaluation period. If DLT is observed in the first participant in the 42-day DLT evaluation period, one more participant can be enrolled in the 30 μL group or terminate the clinical trial after cautious evaluation.

The condition of the first participant in each dose group will promptly apply to the ethics committee for follow-up review as a report. The researchers would apply and adjust the number of subjects strictly according to the study results.

Dose limiting toxicity (DLT)

Dose limiting toxicity (DLT) is evaluated within 42 days after administration of RRG-003 according to Common Terminology Criteria for Adverse Events, NCI-CTCAE V5.0. DLT is defined as hematologic toxicity ≥ grade 4, nonhematologic toxicity ≥ grade 3, or aural toxicity in grade 2 or 3 within 6 weeks. Aural toxicity was listed as follow:

Adverse Events	Grade 2 ear adverse events	Grade 3 ear adverse events
Ear pain	Moderate pain; limiting instrumental Activities of Daily Living (ADL)	Severe pain; limiting self care ADL

External ear pain	Moderate pain; limiting instrumental ADL	Severe pain; limiting self care ADL
Hearing impaired (Pediatric)	Pediatric (on a 1, 2, 3, 4, 6, and 8 kHz audiogram): Threshold shift >20 dB at 4 kHz in at least one ear	Pediatric (on a 1, 2, 3, 4, 6, and 8 kHz audiogram): Hearing loss sufficient to indicate therapeutic intervention, including hearing aids; threshold shift >20 dB at 2 to < 4 kHz in at least one ear
Middle ear inflammation	Serous otitis, medical intervention indicated	Mastoiditis; necrosis of canal soft tissue or bone
Tinnitus	Moderate pain; limiting instrumental ADL	Severe pain; limiting self care ADL
Vertigo	Moderate pain; limiting instrumental ADL	Severe pain; limiting self care ADL
Vestibular disorder	Moderate pain; limiting instrumental ADL	Severe pain; limiting self care ADL
Ear and labyrinth disorders - Other, specify	Moderate; minimal, local or noninvasive intervention indicated; limiting age-appropriate instrumental ADL	Severe or medically significant but not immediately life-threatening; hospitalization or prolongation of existing hospitalization indicated; limiting self-care ADL
Otitis externa	Oral intervention indicated (e.g., antibiotic, antifungal, or antiviral)	IV antibiotic, antifungal, or antiviral intervention indicated; invasive intervention indicated
Otitis media	Localized; oral intervention	IV antibiotic, antifungal, or

	indicated (e.g., antibiotic, antifungal, or antiviral)	antiviral intervention indicated; invasive intervention indicated

4. Subject inclusion criteria, exclusion criteria, rejection criteria and suspension criteria

4.1 Inclusion Criteria

1. Paticipants and their guardians can fully understand and voluntarily sign the informed consent of this study, willing to cooperate with follow-up at the specified time points in the trial.
2. Be able to communicate well with the researchers and comply with the requirements under the help of the guardian. For young children without mature language skills, they could cooperate and comply with the requirements under the help of guardians.
3. A proper understanding of the trial and an appropriate expectation of the benefit.
4. 3-10 years old, gender is not limited.
5. A diagnosis of DFNB9 congenital deafness was determined based on the clinical symptoms, and gene mutation analysis for the presence of either homozygous or bi-allelic mutations in *OTOF*.
6. Audiology includion criteria: Severe to complete hearing loss (≥65 dB)
7. Satisfy the requirements for otological surgery: conditions such as middle-inner ear deformity, vestibular-cochlear nerve development abnormality, and ear inflammation, etc. are excluded through CT scan and/or MRI scan within 3 months or during screening.

4.2 Exclusion Criteria

1. Gene analysis does not suggest any *OTOF mutation*, or concomitant with other gene mutations causing hearing loss.
2. Other types of deafness that are not suitable for otological surgery, such as conductive deafness, mixed deafness, malformation syndrome caused by middle - inner ear dysplasia or malformed, abnormalities of vestibular nerve or cochlear nerve through CT/MRI scan within 3 months.
3. Pre-existing ontological diseases that may interfere with the interpretation of study endpoints, such as: acute-chronic otitis media, Meniere's disease, acoustic neuroma or unrecovered sudden deafness, etc.
4. A history of substance abuse, any ototoxic drug treatment (such as aminoglycosides, cisplatin,

loop diuretics, etc.) within 6 months, antiviral or immunization therapy within 3 months, or vaccination within 1 month.

5. A history of complex immunodeficiency, or organ transplantation.
6. Patients with severe systemic disease or active bacterial or viral infection, such as pulmonary tuberculosis, active hepatitis B or C infection, active herpes zoster infection, pancreatitis, renal failure, or gastrointestinal ulcers.
7. Patients with contraindications to surgery or anesthesia certified by the surgeon, anesthesiologist or designee, such as an allergy to the study medication, cardiovascular or cerebrovascular accident which occurred within the past 6 months, including myocardial infarction, heart failure, angina pectoris, cerebrovascular accident or transient ischemic attack.
8. Currently participating in or planning to participate in another clinical trial involving a drug or device within 1 year, or within 5 half-lives after the last dosing in another clinical trial.
9. Bilateral ear implants (e.g., cochlear implants).
10. With > 1:2000 neutralizing antibodies against AAV1 capsid.
11. Other severe congenital diseases.
12. A clear history of neurological or psychiatric disorders, including epilepsy or dementia.
13. Patients who require long-term use of anticoagulants.
14. A history of radiotherapy and chemotherapy.
15. Other conditions that investigators do not consider appropriate for participating in the present clinical study.

4.3 Suspension criteria

1. Unsuccessful administration. Drug administration is not completed according to the protocol.
2. The participants withdraw from the test or loss of follow-up.
3. Allergic reaction or serious adverse events. Due to the obvious abnormalities of drug safety related laboratory indicators or adverse events (such as severe allergic reactions) during administration process, the investigator and/or the sponsor considers that it is not appropriate to continue injecting drugs based on ethical consideration. People who need rescued due to serious adverse events. Complicated by other serious diseases during the trial period, and the investigator judges that it is not appropriate to continue to participat in the trial;

4. During the 6-week evaluation period, the original condition progresses or other treatments are required: if no treatment measures are taken temporarily, they can continue to participate in the trial or withdraw from the trial at the discretion of the investigator; if other treatments are required, the trial should be withdrawn;
5. Serious violation of trial protocol. The participants or the participants' guardian refused to follow-up or keep serum samples, and the combined use of prohibited drugs that significantly exceeds the dosage may bring risks; the required inspection items are not complete as planned, resulting in an inability to assess safety and efficacy; the informed consent process is incomplete or there is no informed consent procedure; the participant's guardian did not sign the informed consent or temporarily withdrew the informed consent.

The original data and documents of the selected or enrolled subjects should retain for retention and intentionality analysis (ITT), whether they drop out or not.

5. The number of cases required to achieve the intended purpose of the study based on statistical principles

According to the description above, the total number of effective cases was 2-3. Two dose groups were set for administration, and the exploration dose was set as 30 μL (9.0E11 vg/ear), 50 μL (1.5E12 vg/ear). The exploration age was 3-10 years old. An effective case was defined as compliance with the protocol and observed for 6 weeks at least. The actual number of subjects can be adjusted according to trial requirements.

6. The dosage form, dose, route of administration, method of administration, frequency of administration, course of treatment, and related regulations for combined medications, as well as instructions for packaging and labeling

6.1 General information

- **Generic name:** RRG-003 ear injection
- **Product name:** Not available
- **Study code:** RRG-003
- **Active ingredients:** Two AAV1 gene therapy vectors, AAV-OTOF-N and AAV-OTOF-C,

carrying the N-terminal and C-terminal coding sequence of human otoferlin, respectively.

- **Characteristics:** The contents of this product are colorless transparent or slightly yellow liquid.
- **Dosage:** 30-50 μL.
- **Administration route:** Injection through the round window following the stapes punched.
- **Administration method:*****
- **Number of doses:** one time.
- **Treatment course:** one time.
- **Specification:** AAV-OTOF-N and AAV-OTOF-C
- **Lot number:** To be determined.
- **Expired period (tentative):** 24 months
- **Storage condition:** ≤-65°C
- **Entrusted manufacturer:** Shanghai Refreshgene Therapeutics Co., LTD.
- **Manufacturer:** Guangzhou PackGene Biotechnology Co., LTD

RRG-003 was packaged in small boxes, each containing one vial of AAV-OTOF-N and one vial of AAV-OTOF-C. Preparation buffer and blank penicillin vials are also provided for mixing prior to clinical administration.

All packaging and drug labels are marked "for clinical trial use only". If there are quality problems such as breakage and discoloration, another box of drugs could be taken and the sponsor would be contacted in time to reissue the drugs.

6.2 Administration Proposal

6.2.1 Preoperative care

1. Clean the external auditory canal thoroughly before surgery.
2. Daily ultraviolet (UV) disinfection in the ward.
3. Nurses participate in the preoperative discussions to understand the surgical methods, objectives, methods, procedures, required equipment, and nursing requirements.
4. Familiar with the possible postoperative complications, such as infection, dizziness, facial paralysis, bleeding, labyrinthitis, and wound nonhealing, along with the observation and report in time.

6.2.2 Steroids Prophylaxis

Dexamethasone intravenous infusion usage based on the body weight, starting on the -3 day and lasts until the 5th day of the post-operation, to reduce the postoperative inflammation and immune response to AAV medications.

6.3 Surgical steps

1. ***

6.3.1 Postoperative care

1. Before waking up from general anesthesia, the patient was lay down on the pillow with the head turned to one side. After waking up, take the supine position and do not press the injected ear. Do not move the head excessively. According to the doctor's advice, give ECG monitoring (closely observe the changes of vital signs), oxygen inhalation, and blood oxygen saturation monitoring (SPO_2 above 90%).
2. Intravenous injection of third-generation cephalosporin antibiotics start on the day of post-operation lasts for 5 days to prevent infection. Glucocorticoids could be used appropriately if accompanied by obvious acute dizziness.
3. Observation and nursing of whole brain syndrome. Closely observe the changes of vital signs, and continuously monitor the patient's consciousness, pupil, blood pressure, pulse, and respiration after waking up from general anesthesia. If the patient vomits, attention should be paid to distinguish whether it is a reaction after general anesthesia or an intracranial complication. If the blood pressure rises progressively, the pulse is slow and powerful, the breathing is deep and fast, the body temperature rises, and headaches are accompanied by changes in consciousness and pupils, one should be alert to the occurrence of intracranial complications and immediately report to the doctor for treatment.
4. Observation and nursing of local wound conditions. Observe whether the ear dressing oozed blood, keep the external ear canal clean, and prevent sewage from entering the ear. Do not blow your nose hard, and wipe it off gently to prevent backflow to the middle ear causing infection.
5. Observation and nursing of facial nerve function. Pay attention to whether there is injury of the facial nerve and chorda tympani nerve, focusing on observing whether there are facial

paralysis manifestations such as crooked mouth corners, inability to close eyelid, reduction or disappearance of forehead lines, and taste alteration. Glucocorticoids, neurotrophins and physiotherapy should be applied once facial paralysis occurs.

7. Items of clinical and laboratory examinations to be performed, examination times, the amount of blood samples or tissues collected each time, sample collection times, and the total amount of samples to be collected

Timeline/Procedure	Baseline	Day											Week					
		-3	-2	-1	0	1	2	3	4	5	6	7	2	4	6	13	26	52
Informed consent	X																	
Selected/excluded subjects	X																	
Next generation sequencing	X																	
Demography data	X																	
History of alcohol and drug dependence	X																	
Medical history/treatment history/family histor	X													X	X	X		X
Physical examination	X													X	X	X		X
Vital signs	X		X		X	X	X	X	X	X	X	X	X	X	X			X
Urinalysis	X		X					X				X	X	X	X			
Blood collection	X		X					X				X	X	X	X	X		
Blood routine	X		X					X				X	X	X	X			
Serum Biochemistry	X							X				X	X	X	X			
Coagulation function	X																	
Virology examination (HIV, HBV, HCV)	X																	
Circulating blood qPCR	X											X						
ELISpot	X														X	X		
Serum anti AAV1 antibody	X														X	X		
12 lead electrocardiogram (ECG)	X		X												X			
Chest X-ray examination	X																	
MRI (ear, vestibule, brain)	X														X			X
Middle Inner Ear CT	X																	
Pure tone audiometry	X													X	X	X	X	X
ABR	X													X	X	X	X	X
DPOAE	X													X	X	X	X	X
ASSR	X													X	X	X	X	X
Speech recognition	X															X	X	X
Vestibular function	X							X					X	X	X	X	X	X
Otoscopy examination	X							X				X	X	X	X			X
Ear care					X	X	X	X		X		X						
Inner ear injection administration					X													
dexamethasone		X	X	X	X	X	X	X	X	X								
Adverse event evaluation	X					X	X					X	X	X	X	X	X	X

7.1 Observation indicators

7.1.1 Demographic information: age, gender, height and weight

7.1.2 Present history, past history and family history: medical history, previous treatment history, other disease history and family history of the DFNB9 children;

7.1.3 Disease observation indicators (screening period): Gene sequencing, otoscopy examination, ABR (Auditory Brainstem Response), DPOAE (Distortion Product Otoacoustic Emission), PTA (pure tone audiometry), ASSR (Auditory Steady State Response), speech recognition rate (for infants and toddlers who cannot cooperate, methods of "family game", video observation and questionnaire survey can be used for evaluation), MRI (internal auditory canal), CT (middle inner ear) and vestibular function examination (for those who can cooperate).

7.2 Tolerability and safety observation indicators

7.2.1 Vital signs: heart rate, respiration, temperature, blood pressure;

7.2.2 Physical examination: major organs and systems;

7.2.3 Neurological examination: MRI;

7.2.4 Laboratory tests: blood biochemistry, coagulation function, blood and urine routine, anti-AAV neutralizing antibody, ELISpot, blood qPCR for AAV shedding detection;

7.2.5 Electrocardiography: standard 12-lead ECG;

7.2.6 Imaging examination: CT or MRI;

7.2.7 Specialist medical examination: external ear and ear canal, tympanic membrane, tympanic chamber, mastoid process;

7.2.8 Vestibular function assessment: if coopetate;

7.2.9 Adverse events.

7.3 Preliminary curative effect observation indicators

7.3.1 Pure tone audiometry test (if cooperate);

7.3.2 ABR test;

7.3.3 ASSR;

7.3.4 DPOAE;

7.3.5 Speech recognition: speech assessment methods include questionnaires and speech tests. 1-3 years old children questionnaire survey adopts “Infant Toddler Meaningful Auditory Integration Scale (IT-MAIS)”, 3 years old and above children questionnaire survey adopts “Meaningful Auditory Integration Scale (MAIS)”. For the speech assessment of children aged from 1 to 6 years old, “Criteria and Methods for Assessing the Language Ability of Hearing-Impaired Children” from Sun Xibin’s is adopt, and three general tests of speech clarity, listening and picture recognition, and imitation sentence length are used for children under 3 years old. Evaluation data of Mandarin Speech Perception (MSP) made by Qianjie Fu will be used for speech assessment of 7-14 years old children.

7.4 Biological sample collection, processing and testing

7.4.1 Urine sample collection (urine routine);

7.4.2 Blood and serum collection (blood routine, blood biochemistry, coagulation function, neutralizing antibody, ELISpot, circulating blood qPCR)

8. Registration and usage records, delivery and distribution methods and storage conditions of investigational drugs

RRG-003 should be refrigerated at ≤ -65°C. All investigational drugs should keep in the central

(good clinical practice, GCP) pharmacy or phase I special pharmacy, and kept, issued and dispensed by dedicated personnel. The Trial Drug Registration Form should be filled out, signed and confirmed by the drug administrator and investigator each time the drug is issued and returned. Store the trial drug in a refrigerator that only authorized personnel can open until it is used or returned to the trial drug provider. The investigator should ensure proper storage conditions and regularly check and verify the quantity and storage of the drug. All test drugs provided by the investigational drugs provider only be used for experimental research purposes and shall not be used for purposes other than those specified in this protocol. The investigator must undertake not to provide the investigational drugs to anyone not related to the trial.

9. Clinical studies, follow-up and measures to ensure subject compliance

In this study, patients will be taken care in hospital during the operation and per-operative periods. For guaranteeing the compliance during the surgical period, the inspection would be carried out. Since this clinical study is a lifetime single dose regimen, there are no compliance issues in terms of medication administration. During the follow-up phase, patients will be contacted for checkups at the hospital, and testing accommodation and testing costs during this period are covered by a dedicated fee. If the patients encounter special circumstances that prevent them from coming to our hospital for examination, they will be contacted to go to the local hospital for examination.

**Pre administration**	↓	↓	↓														
**RRG-003 administration**				↓													
**Screening period**	Day-3	Day 2	Day-1	Day0	Day1	Day2	Day3	Day4	Day5	Day6	Day7	W2	W4	W6	W13	W26	W52
**Blood collection** ↑		↑					↑				↑	↑	↑	↑	↑		
**Hospitalization**	↑	↑	↑	↑	↑	↑	↑	↑	↑	↑	↑						
**follow-up**												↑	↑	↑	↑	↑	↑

10. Criteria for suspending the clinical study, regulations for ending the clinical study

1) The principal investigator believes that the drug own potential safety risks and continuing the trial is not conducive to protecting the rights and interests of the subjects;

2) There are major defects in the trial protocol and the trial cannot be carried out smoothly;

3) The sponsor requests termination；

4) The ethics committee or drug regulatory authority requires termination of the trial;

5) Other cases that the investigator considers it is not appropriate to continue the study or considers it difficult to proceed the study.

11. Efficacy assessment criteria, including methods for evaluating parameters, observation time, recording and analysis

Focusing on the purpose of the study, involving the efficacy assessment must have evaluation indicators or observation indicators, including primary and secondary evaluation indicators. At the same time, corresponding evaluation standards should be established for these indicators.

Primary indicators: tolerability (dose-limiting toxicity, DLT).

Secondary indicators: safety, preliminary efficacy.

Initial efficacy:

-Pure tone test: change in pure tone audiometric thresholds at each follow-up evaluation point compared to the pre-treatment baseline: a stable reduction of 20 dB at any one of the PTA audiometric frequencies or a stable reduction of 10 dB at any two audiometric frequencies is considered clinically significant;

-ABR test: change in ABR thresholds at each frequency at each follow-up evaluation point compared to the pre-treatment baseline;

-ASSR: change in ASSR thresholds at each frequency at each follow-up evaluation point compared to the pre-treatment baseline;

-Speech recognition: change in speech recognition rate at each follow-up evaluation point compared to the pre-treatment baseline. Speech was assessed by questionnaires and speech tests, using the Infant toddler meaningful auditory integration scale (IT-MAIS) for children 1-3 years of age and the Meaningful Auditory Integration Scale (MAIS) for children 3 years of age and older. For the speech assessment of children aged 1-6 years, Sun Xibin's “Criteria and methods for assessing the language ability of children with hearing impairment” will be used. The Chinese speech assessment materials (Mandarin Speech Perception (MSP)) by Qianjie Fu will be used for children aged 7-14 years.

12. The storage procedure of subject's code, random number table, and case report form

Each participant will be given a unique screening number, and enrolled participants will be given a unique enrollment number. The trial will be conducted using single-arm sequential enrollment without randomization. Beginning with screening, a dedicated case report is established for each subject. Relevant storage procedures are handled in accordance with the management regulations of clinical research institutions.

13. The recording requirement of adverse event (AE) and the reporting, handling, follow-up visit, timing and outcome of serious AE

13.1 Definition

13.1.1 Adverse Event (AE)

AE is any adverse medical event observed after the use of a trial drug in the subject, regardless of whether it is treatment related.

The difference between an AE and an adverse reaction: an adverse reaction means that there is a causal relationship between therapeutic drug and adverse reaction; an adverse event indicates that the causal relationship between therapeutic drug and adverse event is not confirmed, requiring further analysis and evaluation.

13.1.2 Serious Adverse Event (SAE)

SAEs are any undesirable clinical events occurring at any dose, such as death, life-threatening, hospitalization or prolongation of hospitalization to treat AEs, persistent or significant disability, congenital anomaly or birth defect.

An adverse event should be reported to the sponsor by the research center within 24 hours if the adverse event meets one of the following criteria and is diagnosed as serious adverse event:

Death: the death of subject resulting from adverse event;

Life-threatening: in the opinion of the investigator, an adverse event is likely to result in the immediate death of the subject without medical intervention, excluding the adverse event that occurs more seriously, and already results in death;

Hospitalization: admission to the hospital that is a result of the adverse event, not including emergency or outpatient visit;

Prolongation of hospitalization: adverse event occurs during the subject's hospitalization, which leads to the prolongation of hospitalization;

Persistent or significant loss of function or disability: the subject is unable to perform normal daily activities. Loss of function does not include relatively minor medical events, such as headache, nausea, emesis, diarrhea, influenza and accidental trauma (e.g., sprained ankle);

Important medical event: the event may not result in immediate death, life-threatening or hospitalization, but the important medical event may jeopardize the subject and require medical or surgical intervention to prevent any of the events above (i.e., death, life-threatening, hospitalization or prolongation of hospitalization, etc.). For example, allergic bronchospasm requiring treatment in the emergency room or at home, convulsions that do not require hospitalization, cachexia, drug dependence or substance abuse, etc.

The hospitalization due to the progression of the original disease is not considered as serious adverse event. The hospitalization resulting from elective surgery and examination or other treatments that are scheduled prior to entering into this trial, or social reasons.

13.1.3 Adverse Drug Reaction (ADR)

For the drug not yet approved for marketing, an adverse reaction is an uncomfortable and unexpected reaction associated with the administration of drug at any dose. There is a causal relationship, or at least a reasonable possibility, between the adverse reaction and the administration of the drug.

13.1.4 Unexpected Adverse Drug Reaction (UADR)

An unexpected adverse drug reaction is an adverse reaction, the nature or severity of which is not consistent with the current product information (e.g., expected adverse events not listed in the investigator's brochure).

The "expected" or "unexpected" adverse reaction is distinguished on the basis of the events observed previously and cannot be predicted from the pharmacological property of drug: ① for drugs not yet approved for marketing, the investigator's brochure is used to determine whether the nature or severity of adverse reaction is consistent with the information described in the investigator's brochure; ② new clinically meaningful information on the nature and severity of a known and documented serious adverse event is also classified as an unexpected adverse reaction.

13.2 Severity of adverse event

The severity of adverse event, not associated with ear, should be judged according to the NCI-CTCAE v5.0.

The severity of anticipated adverse events in the ear is defined in terms of mild, moderate, severe, and life-threatening:

Mild/Grade 1: discomfort that not interfere with daily activities; children at 8 kHz with a threshold shift of 20 dB hearing loss; sensorineural hearing loss above 4 kHz in at least one ear.

Moderate/Grade 2: discomfort interfering with daily activities; children at 4 kHz with >20 dB threshold shift in one ear

Severe/Grade 3: inability to work or perform daily activities; hearing loss requiring therapeutic intervention, including hearing aids; threshold shift of 20 dB in at least one ear at ≥2 kHz.

Life-threatening/Grade 4: Adverse event may lead to death; cochlear implant; threshold shift of >40 dB of hearing loss, sensorineural hearing loss at >2 kHz.

Death/Grade 5.

13.3 Judgement of the causal relationship between adverse event and the investigational drug

At present, the causal relationship between adverse event and medication administration is usually classified into 5 cases in China and internationally:

① Definite Related: adverse event occurs explained by drug administration;

② Probably Related: adverse event occurrence probably highly related to drug administration;

③ Possibly Related: adverse event occurrence possibly related to drug administration;

④ Unlikely to be Related: adverse event occurs more likely to be related to another factor;

⑤ Not Related: adverse event due to other significant factors.

Items ① to ③ above should be recorded as adverse drug reactions. Incidence of adverse reaction = number of (① + ② + ③) cases/number of cases in safety data set × 100%.

The causal relationship between adverse event and medication administration

	① Definite Related	② Probably Related	③ Possibly Related	④Unlikely to be Related	⑤Not Related
Temporal relationship: a reasonable temporal relationship between drug	+	+	+	+	-

administration and the occurrence of reaction / event					
Known: the reaction that is consistent with known adverse reaction of drug	+	+	+	-	-
Dose-response relationship: disappearance or reduction of reaction/event after drug withdrawal or dose reduction	+	+	±	±	-
Recurrence: reaction/event that reoccurs after a rechallenge	+	?	?	?	-
Explainable: reaction/event that is not explained by the effect of the concomitant medications, the progression of subject's condition, the effect of other treatments.	+	+	-	±	-

13.4 Handling of adverse event

In the event of an adverse event, the investigator decides whether to terminate the trial based on the subject's condition; in the event of a serious adverse event, the investigator must immediately conduct the correct treatment or rescue treatment to protect the subject's safety.

The main measures to be taken in the event of an adverse event include:

1) observation only;

2) dosage adjustment of the investigational drug or dose interruption;

3) withdrawal of the investigational drug;

4) treatment of concomitant medications;

5) non-pharmacological therapies;

6) hospitalization or prolongation of hospitalization.

13.5 Recording of adverse event

Serious adverse events occurring after the acquirement of the Informed Consent Form and before the end of the trial; all adverse events need to be recorded in the corresponding section of the CRF after the acquirement of the Informed Consent Form until subject out, regardless of whether the adverse event is related to the investigational drug unless the subject withdraws the informed consent and follow-up visit cannot be performed.

All adverse events should be recorded in concise medical terms include ① a description of the adverse event and all associated symptoms; ② the time of occurrence and the duration of the adverse event; ③ the severity of the adverse event; ④ the examinations and treatments performed due to the adverse event; ⑤ the final outcome of adverse event; ⑥ judge whether the adverse event was related to the investigational drug.

13.6 Reporting of serious adverse event

All serious adverse events should be reported by the investigator to the sponsor and the If all serious adverse events are judged as serious adverse events, the investigator should report to the sponsor and the drug regulatory department within 24 hours, regardless of whether the adverse events are related to the investigational drug or trial operations. The sponsor has the responsibility for reporting the serious adverse event to the Department of Drug Administration and other clinical trial centers for the same drug in accordance with the regulations. Unless another requirement from the local regulatory authority and other documentation from the Ethics Committee, the investigator should also report the serious adverse event to the Ethics Committee that approved the protocol in accordance with the regulations.

The investigator fills in the Serious Adverse Event Report Form with a detailed description of the course of adverse event, the diagnosis, the treatment given, and the possible relationship to the drug. Follow-up reports should be completed and reported to all parties according to the initial reporting procedures if the diagnosis is unclear or changed after it has been reported, or there is an important change in the subject's condition (e.g., the adverse event progresses to death from prolongation of hospitalization).

Serious adverse events should be sent by the investigator to the sponsor or the supervisor by fax:

Fax number:

Contact for the sponsor:

Phone number:

The sponsor should report the serious adverse event to the following department within 24 hours after receiving the Serious Adverse Event Report Form from the investigator:

China Food and Drug Administration, Department of Research Supervision:

Shanghai Food and Drug Administration, Registry:

When the investigator and sponsor report serious adverse event to related departments, the relevant correspondence records should be stored, such as fax-sending reports, handover documentation, telephone records, etc.

13.7 Trail and follow-up visit of adverse event

All adverse events should be followed up with detailed documentation of handling process and outcome, until they are appropriately resolved or stable. Any laboratory test results that are still abnormal at the end of the trial should be followed up by the investigator until they return to be normal or are clinically stable. According to the severity of adverse event, the distance of the subjects' residence from the hospital, and the medical specialty of the trial center, various forms of follow-up visit can be selected, such as inpatient, outpatient, home visit, telephone, and communication. The outcomes of all adverse events should be recorded in the CRF timely, and if the adverse event has not recovered by the end of the trial, the investigator should continue to follow the subject and record the relevant results in the medical record.

All serious adverse events should be followed up until the events are fully resolved; and at least one follow-up or summary report should be provided to detailed describe the treatment given to the subject, the outcome of subject’s condition, the final diagnosis, and the relationship between final diagnosis and investigational drug, since the onset of the serious adverse events. The summary report of the serious adverse events should be reported to the sponsor and the Ethics Committee in accordance with the initial report procedures, and should be also reported to the Department of Drug Administration by the sponsor.

13.8 Expected adverse reaction and handling

13.8.1 Risk assessment of the trial

Based on the clinical trial data from systemic administration of AAV drug, the risk of serious

adverse events associated with AAV drugs includes hepatotoxicity (elevated liver enzymes, liver failure, etc.), thrombocytopenia, hemolysis, anemia, acute kidney injury, and neurotoxicity found on MRI. The local administration of AAV drug in the clinical trial such as ophthalmic trial gives rise to few systemic toxicity, mainly local inflammation at the site of administration and systemic reactions due to hormone administration at the same time.

Based on the data from drug administration by injected into inner ear of patients with profound deafness in clinical trials, adverse reactions may be associated with AAV drugs, injection procedures, concomitant corticosteroids or a combination of these procedures and products. The most common adverse reactions (>5%) in the ear include mild hearing loss, dizziness, lightheadedness, nausea, and vestibular dysfunction, etc. Other adverse reactions with an incidence of less than 5% include otalgia, ear bleeding, unilateral deafness, tinnitus, taste change due to the injury of chorda tympani nerve, otitis media, perilymphatic fistula, reparative granuloma and tympanic membrane perforation, facial palsy, inner ear infection and meningitis, and sinusitis, etc. Systemic adverse events include hepatic impairment (elevation of ALT, AST, etc.), fatigue, food allergies, seasonal allergies, atopic dermatitis, urticaria, etc. These are systemic events that may be caused by systemic administration of corticosteroids and anesthetic reactions.

In summary, this clinical trial may be exposed to risks caused by the above-mentioned adverse reactions and risk control will be performed during the clinical trial to ensure the safety of the subjects.

13.8.2 Treatment Precautions

In accordance with the regulations of NMPA's new GCP, patients who are ready to participate in the trial will be screened strictly according to the inclusion and exclusion criteria, avoiding to include patients who do not meet the criteria in the trial. To guarantee the safety of subjects, risk control will be carried out during the trial and is planned as follows:

(1) Multiple follow-up visits will be performed before dosing, during single-dose treatment and thereafter, including subject's general condition, clinical signs and symptoms, any adverse events during discharge, medical physical examination, observation of injection site reactions and other adverse events, and otologic examinations. Otologic examinations include ear inflammation, ear infection, ear bleeding, pure tone test, speech recognition, ABR, DPOAE, vestibular assessment, etc. Laboratory tests include blood, urine, function of liver and kidney, blood electrolytes,

coagulation. Monitor the occurrence of adverse events specially. The above contents include the safety indicators for RRG-003, aiming to control possible adverse drug reactions and risk factors during the trial.

(2) Monitor and control possible adverse drug reactions and risk factors during the trial by signs and symptoms, laboratory tests, and otologic imaging; and give advice on handing.

(3) The subject can withdraw from the trial at any time during any phase of the trial.

(4) The safety and efficacy data from this trial are regularly analyzed by the principal investigators and/or statisticians, who decide whether the trial should be continued.

(5) If the subject experiences an adverse event related to the investigational drug during the trial, the following principles need to be followed for handing of adverse event.

If an accident occurs during inner ear surgery, terminate injection promptly. And medical treatment should be conducted by the investigator;

Adverse events that do not meet the DLT during the observation of DLT are not treated generally;

After completion of DLT observation, adverse events should be handled actively;

Once adverse event happens in the subject, the investigator is responsible for making relevant medical decisions based on clinical practice to ensure that the subject is treated appropriately and the handling of AE should be documented.

(6) Risk of infection after inner ear drug administration

Nurse the subject carefully during the perioperative period of inner ear surgery; prevent infection with antibiotics and glucocorticoids after surgery. Contact the investigator promptly if subject fails to comply with medical advice or an accident occurs.

(7) Other toxic reactions: Symptomatic treatment is available. If blood tests and urine tests are abnormal, collect and detect samples again within 24 hours; and the corresponding treatment or therapy should be performed according to the test results.

(8) Risks associated with dexamethasone via intravenous injection:

Dexamethasone can reduce postoperative inflammation and decrease the immune response to AAV drugs, and is often used before and after ear surgery.

Common side effects of dexamethasone include increased appetite, stomach upset, nervousness, or restlessness. Less common but serious side effects include decreased or blurred vision, fluid retention, weight gain, increased blood sugar level, frequent urination, thirst, mood swings,

confusion, and rash or urticaria. Other rare side effects include darkening or brightening of the skin, dizziness, facial flushing, hiccups, and increased sweating.

Dexamethasone may reduce resistance to the infection, which may lead to a sore throat, fever, sneezing or coughing.

Once side effects occur, discontinue dexamethasone immediately.

(9) General anesthesia:

Serious but very rare adverse events (negative side effects) associated with any types of anesthesia/surgery include seizures, coma, and death.

Rare but serious complications associated with general anesthesia include cardiac arrhythmias, increased or decreased blood pressure, transient high fever, rare reactions to drugs used in anesthesia, and airway obstruction.

The risk associated with a small amount of local anesthesia is low. Anesthetic injection is accompanied with a small risk of penetration of the eye or optic nerve, and the possibility of hemorrhage, which need to be treated if necessary.

(10) Risks associated with surgery:

1. Injury to ear ossicles such as injury to stapes, resulting in the dislocation of stapes; or even injury to oval window, leading to perilymphatic fistula.

2. Injury to jugular bulb, causing hemorrhage. In the event of hemorrhage, the ear canal should be filled with pressure to stop bleeding.

3. Facial paralysis, is usually caused by injury to the facial nerve or excessive strain on the chorda tympani nerve. If facial paralysis occurs, analyze the cause and perform facial nerve decompression surgery timely if necessary.

4. Labyrinthitis: nurse surgical cavity carefully after surgery; and use antibiotics and glucocorticoids that can cross the blood-brain barrier to prevent the spread of inflammation; persist with follow-up visit.

5. Post-operative infection, short or long-term ear discharge, perichondritis. Strictly grasp the surgical indications and contraindications before surgery and pay attention to aseptic operation during surgery. Dressing should be changed after surgery, and granulation in the surgical cavity should be treated in time to protect the new epithelium. Pay attention to drainage and removal of secretions.

6. Tinnitus.

7. Cerebrospinal fluid leak. Promptly repair it with mastoid cortex and temporalis fascia.

8. Taste change, usually caused by injury of chorda tympani nerve.

(11) Thrombocytopenia:

1. Hemostasis: in the event of hemorrhage, stop bleeding via antihemorrhagic, prevent internal bleeding, and then identify the cause.

2. Hormone therapy: In the event of mucous membrane or more extensive skin bleeding, treat the subject with adrenocortical hormone to control the condition and reduce the damage to the body. Use estrogen for a short time in an acute condition.

3. Blood transfusion: When a platelet count is less than 30*10^9/L with continuous bleeding, platelet transfusion should be performed in time to stop bleeding as soon as possible.

In conclusion, according to the risk control plan, the possible risks of RRG-003 during the trial are controlled, and suggestions are med for the treatment of possible adverse reactions, ensuring participants take drugs reasonably and safely.

14. The establishment and maintenance of investigational drug code, the method of unblinding, and the rule of unblinding in the event of an emergency

Not applicable.

15. Statistical analysis plan, definition, and selection of statistical analysis data set

15.1 Definition and selection of analysis data set

Enrolled Patients Set (EPS): defined as all enrolled cases, with or without investigational drug. The data set is used to analyze data such as demography and baseline information, subject allocation, and protocol violation.

Safety Analysis Set (SAS): defined as all enrolled cases that use the investigational drug at least once. The data set is used to analyze safety data.

Efficacy Evaluable Analysis Set (EEAS): defined as all enrolled cases who had at least one efficacy assessment after receiving investigational drug. The dataset is used for analysis of preliminary efficacy data.

Full Analysis Set (FAS): Defined as all enrolled cases who receive investigational drug at least

once and have at least one post-baseline efficacy assessment according to the intention-to-treat (ITT) principle. The dataset is used for efficacy analysis.

Per-Protocol Set (PPS): defined as all cases, incorporated in the full analysis set, who comply with the protocol, have good medication adherence, do not use prohibited drugs during the trial, and have a defined efficacy assessment in the trial. The dataset is used for efficacy analysis.

Statistical analysis methods

15.2 Statistical analysis plan

Descriptive statistics are primarily used to analyze trial results. In general, measurement data will list the number of observational cases, mean, standard deviation, median, quartile, maximum and minimum. Enumeration data will list frequency and relative frequency. Unless otherwise specified, all of statistical tests will perform two-tailed test with a 95% confidence interval. Statistical analysis will be performed in a version of SAS 9.4 or higher version.

Use descriptive statistics to describe the number and proportion of subjects entering each analysis set, the number and proportion of subjects completing the trial and withdrawing consent, and the reasons for withdrawing consent (and proportion of subjects) for each dosage group; to describe the baseline characteristic of each dosage group.

Use the Safety Analysis Set to summarize treatment-emergent adverse event (TEAE) and adverse reaction for each dosage group; the AEs, occurs after signing informed consent and before treatment, will be only tabulated based on per subject and not summarized. TEAE is defined as the adverse event that is not present before dosing and occurs within 28 days (inclusive) after one administration. The severity of adverse event and adverse reaction will be graded based on the NCI-CTCAE v5.0. Adverse event and adverse reaction will further be summarized based on System Organ Class and Preferred Term.

Using the safety analysis set baseline data, post-dosage data, and change from baseline were summarized for each follow-up visit and each dosage group, according to safety data such as laboratory, vital signs, electrocardiogram, physical examination, and otologic examination. Shift tables will be used to describe change from baseline after each follow-up visit to determine whether each examination result was normal and clinically significant as categorical data.

Use Per-Protocol Set to summarize efficacy data at each efficacy evaluation point and plot the efficacy-time curve of each subject, the mean efficacy-time curve of each dosage group, and the

mean efficacy-curve of all subjects, according to efficacy data change from baseline in each dosage group.

16. Data management and information Confidentiality

The investigator is responsible for data management in this trial.

The data on the eCRF is derived from the original medical records and completed by investigator or the individual specified by investigator, ensuring the completeness and accuracy of the information. If there are errors that need to be corrected, the amendment should be made according to the eCRF completion instructions.

17. Quality control and quality assurance of clinical trial

17.1 Quality control (QC)

17.1.1 Qualification of research institute and investigator

The research institute should have relevant qualifications for conducting the clinical trial of medicinal product; and the facilities and conditions of the department conducting the trial should meet the requirements for carrying out clinical trial safely and effectively. The investigators should have the specialty, qualification and skill to conduct the trial and should be trained for GCP and this protocol. Prior to the start of the trial, the project director of the center should organize the investigators to study the protocol, and only those who are trained for the protocol can participate in the trial. The investigators participating in the trial should be relatively fixed, and those who join the trial during the trial should be trained first, and the clinical trial participator authorization form should be updated timely.

17.1.2 Laboratory quality control measures

The laboratory of trial center establishes standard operating procedures and quality control procedures for laboratory test index and provides appropriate certificate of qualification.

17.1.3 Guarantee compliance

Subject compliance: The process of informed consent and the signing of informed consent form are important aspects of ensuring subject compliance. The investigator should patiently explain informed consent to the subject's guardian, so that the subject's guardian can fully understand the content and process of trial, as well as his or her rights and obligations, like complying with medical prescriptions to take medication on time, follow-up visit on time and completing required clinical

observations and physical and chemical examinations.

Investigator compliance: Investigators should strictly comply with the trial protocol and relevant regulations to carry out clinical trial. Their compliance is mainly reflected in the three main aspects, including selecting qualified subjects, controlling imposed factors, and observing and evaluating effect indicators. The specific contents are as follows.

a) Strictly comply with the trial protocol and regulations, with no serious violations of the protocol;

b) Select qualified subjects to participate in this trial and sign the informed consent form;

c) Strictly comply with the protocol to enroll subjects and perform trial observations and records;

d) Impose reasonable treatment on the subject. All effects, derived from imposed factors (including investigational drugs, concomitant medications) and other measures, can be measured, assessed, and judged with objective, credible, scientific and feasible criteria;

e) Handle, record and report adverse events in time, especially serious adverse events;

f) The trial strictly complies with the standard operating procedure (SOP);

g) Data management and statistical processing strictly comply with SOP.

17.2 Quality assurance (QA)

17.2.1 Good Clinical Practice and the Declaration of Helsinki

The Good Clinical Practice (GCP) and the Declaration of Helsinki are the legal support for maintaining the scientific and ethical principles of this clinical trial, as well as are the legal and regulatory guidelines for this clinical trial. Any person involved in this trial, especially the supervisors and inspectors appointed by the sponsor, has the right to correct any behavior and clinical treatment that violates the GCP and the Declaration of Helsinki. Especially when the rights of the subjects are not guaranteed and protected, the subjects have the right to terminate the clinical trial.

17.2.2 Trial protocol

The protocol is the guiding document of this clinical trial, guiding all investigators involved in this trial how to start and conduct the clinical trial, and it is also an important basis for data collection, recording, reporting and statistical analysis after the trial is completed. Therefore, the formulating a scientific and detailed trial protocol is not only a fundamental and important condition to ensure the success of the trial, but also an important document for quality control and quality assurance performed in the trial.

Before the start of the trial, the first investigators' meeting shall be held to discuss the protocol, the specific steps of the trial operation, the EDC data entry, sample collection methods in detail, The project director of the center, investigator and sponsor attend the meeting. If it is necessary to amend this protocol, either the investigator or the sponsor should proceed with the consent of the other party. All amendment of the protocol will be issued by the sponsor and submitted by the investigator to the Ethics Committee for review or filing.

17.2.3 Statistical analysis plan

The statistical analysis plan is a data guarantee for quality management in the three main aspects: selecting qualified subjects, controlling imposed factors, and observing and evaluating effect indicators.

17.2.4 Informed consent and Informed Consent Form

Prior to the start of the clinical trial, the investigator must provide the subject's guardians with detailed information about the clinical trial, including the nature of the trial, the purpose of the trial, possible benefits and risks, alternative treatments available, and the subject's rights and obligations, etc. The clinical trial can only be carried out after the subject's guardian fully understand and agree, and sign the "Informed Consent Form". Informed Consent is one of trial documents to ensure the safety, rights and health of the subjects are maintained and protected during the trial process, i.e., any clinical trial is conducted on the premise that the health and rights of the subjects are not compromised.

17.2.5 Ethics Committee and Trial Document Review

As Informed Consent Form, the Ethics Committee is an important safeguard to protect the rights of subjects. In addition to reviewing the trial documents, the Ethics Committee audits protocol compliance and the protection and safeguarding of subjects' rights during the trial, in particular the treatment, trail and follow-up visit of subjects in the event of serious adverse events, ensuring that the trial is conducted in accordance with scientific and ethical principles.

Before the trial begins, the protocol should be discussed and revised by the project director and submitted to the Ethics Committee of the trial center for written examination and approval. The investigator should use a copy of the ethical approval as the basis for initiating and conducting this trial. The trial documents submitted to the Ethics Committee for examination and approval include:

1) Submitted and reviewed documentation checklist;

2) Ethics Review Application Form;

3) Clinical Trial Protocol;

4) Subject Informed Consent Form;

5) Recruitment Advertisement;

6) Curriculum Vitae of Principal Investigator;

7) Case Report Form;

8) Quality Verification Report of Investigational Drug;

9) Other relevant materials required by the Ethics Committee.

17.2.6 Supervisor’s Oversight and Sponsor’s Audit

The oversight of the trial is performed by the supervisor appointed by the sponsor to monitor the trial center according to the Standard Operating Procedure (SOP). The supervisor's oversight is one of the important guarantees to ensure the quality of the multicenter trial. The supervisor should communicate with the investigator and sponsor regularly.

The supervisor will assess the capabilities of each trial site and report issues related to facilities, technical equipment, or medical staff to the sponsor. During the trial, the supervisor will be responsible for monitoring that written informed consent is properly obtained from all subjects and that data records are accurately and completely documented. The supervisor also has the authority to compare data entered into the eCRF with the original data and to notify the investigator of errors or omissions. The supervisor will also ensure that the trial site adheres to the protocol, arranges and supplies the investigational drug, and ensures that the drug is kept in appropriate conditions.

The supervisor conducts monitoring visits in accordance with all relevant laws and regulations, and will conduct regular monitoring visits to each center beginning with the enrollment of subjects. After each visit to the investigator, whether by telephone or trial center visit, the supervisor shall submit a written report to the sponsor.

During the monitoring visit, the supervisor will compare the data entered into the eCRF with the original data file (Source Data Verification). This includes, but is not limited to, following items conducted100% verification:

- Subject number;
- Obtaining informed consent from subjects;
- Subject eligibility (inclusion and exclusion criteria);

- Drug handling procedures;
- efficacy index;
- AE medical records.

The sponsor will also dispatch quality assurance personnel to audit the clinical trial. The scope of audit includes the protection of subjects' rights, the implementation of the protocol and the work of investigators and supervisors, etc.

17.2.7 Establish and perfect the SMP and SOP of the center

The related research institutions should establish and improve regulations and Standard Management Procedure (SMP), and strengthen the training of GCP and related regulations for the investigators, ensuring that the trial is going well. Standard Operating Procedure (SOP) of the trial should be established and improved, and implemented and perfected during the trial, making the trial procedural, institutionalized, standardized and formatted.

18. Research relevant ethics

18.1 Examination and approval of trial documents from Ethics Committee before starting the trial

Before starting the trial, the protocol should be discussed and revised by project director of the trial center, and submitted to the Ethics Committee of the trial center for written examination and approval. The center should take ethical approval as the basis of initiation and implementation of the trial.

1) Submitted and reviewed documentation checklist;

2) Ethics Review Application Form;

3) Clinical Trial Protocol;

4) Subject Informed Consent Formtrial;

5) Recruitment Advertisement;

6) Curriculum Vitae of Principal Investigator;

7) Case Report Form;

8) Quality Verification Report of Investigational Drug;

9) Other relevant materials required by the Ethics Committee.

18.2 Subjects undergo the process of informed consent and acquire informed consent before starting the trial

After the official start of the trial and before the screening of the subjects, the investigators should provide informed consent to the guardian of each subject and explain the specific circumstances of the clinical trial to them in detail. The guardian of the subject should sign the informed consent voluntarily on the premise that the guardian of the subject fully understands the trial process and agrees to participate in the trial.

18.3 Any AE that occurred during the trial can be effectively managed and followed up

If any adverse event occurs during the trial, the investigator can decide whether to terminate the trial based on the condition. Once an adverse event occurs, the investigator will immediately implement appropriate treatment or rescue treatment to protect the safety of the subject.

All adverse events will be followed up until they are properly resolved or stabilized. According to the severity of adverse event, the distance of the subjects' residence and the hospital, and the medical specialty of the trial center, hospitalization, outpatient visit, home visit, telephone, and communication can be selected.

RRG-003 meets the requirement of IIT clinical trial in terms of quality standard, animal pharmacodynamics and safety.

This protocol follows the ethical principles of medical research involving human subjects outlined in the Declaration of Helsinki, is consistent with the ethical principles of medical research involving human subjects, and benefits all subjects as far as possible.

Although there is no guarantee that the result of this trial will be as expected, it is possible that the condition of subjects participating in the trial will improve. At the same time, all investigational drugs and related examinations required by the protocol are free.

19. Subject recruitment method and the process of acquiring informed consent

The researchers and their team members will release the information through advertisements posted in the hospital, inquiries during the consultation, and multimedia platforms such as WeChat. When the subjects see the recruitment advertisement and contact the hospital team, they will be

fully informed about the risks and benefits of the procedure before guardians decide whether to voluntarily participate in the trial. After the patient and his/her guardian confirm to participate in the clinical trial, we will issue the informed consent form verbally and in writing one by one, and the patient and his/her guardian will confirm the informed consent one by one.

20. Expected progress and completion date of clinical trial;

During the observation period of the administration, the subject is hospitalized on Day 1-Day 7 and undergoes physical examination, laboratory test, and otologic examination according to the trial flow chart during the follow-up visit on W2, W4, W6, W13, W26, and W52. Safety, tolerability, and adverse events are evaluated on Day 1, Day 2, Day 3, W1, W2, W4, and W6; long-term safety and preliminary efficacy are evaluated on W13, W26 and W52.

21. Follow-up and medical care after completing the trial

Follow-up visits will continue every six months after completion of the trial.

22. Responsibilities of the parties and other relevant regulation

Principal investigator: Seriously perform the duties of the investigator, agree to conduct the clinical trial in accordance with the design and specifications of this protocol, and explain the relevant data, regulations, and responsibilities of the trial in detail to all personnel in the trial. Sufficient time should be guaranteed to carry out and complete the clinical trial within the prescribed period, and all relevant information and data will be truthfully, accurately, completely, timely, and legally entered into the medical records and case report forms. Strictly abide by the Declaration of Helsinki, explain the details of the clinical trial approved by the Ethics Committee to the subject's guardian who acquires informed consent. Be responsible for making medical decisions related to this clinical trial and ensure that subjects receive appropriate treatment in the event of adverse events during the trial. If a serious adverse event occurs during the trial, appropriate treatment will be taken immediately to ensure the safety of the subject, which will be reported to the relevant authorities specified in the GCP.

Sponsor: Responsible for initiating, applying, organizing, supervising, and reviewing the clinical trial and providing trial funding in accordance with GCP standards. In particular, provide therapeutic

compensation for subjects who suffer trial-related damage or death during the trial and provide legal and economic guarantees for the investigators.

Reference

1. Deafness and hearing loss. 2021. at https://www.who.int/news-room/fact-sheets/detail/deafness-and-hearing-loss.)
2. Zhang QJ, Han B, Lan L, et al. High frequency of OTOF mutations in Chinese infants with congenital auditory neuropathy spectrum disorder. Clinical genetics 2016;90:238-46.
3. Akil O, Dyka F, Calvet C, et al. Dual AAV-mediated gene therapy restores hearing in a DFNB9 mouse model. Proceedings of the National Academy of Sciences 2019;116:4496-501.
4. Al-Moyed H, Cepeda AP, Jung S, Moser T, Kügler S, Reisinger E. A dual-AAV approach restores fast exocytosis and partially rescues auditory function in deaf otoferlin knock-out mice. EMBO Molecular Medicine 2019;11:e9396.

The safety, tolerability, and preliminary efficacy of RRG-003 AAV in the treatment of DFNB9 congenital deafness

Principal Investigator: Yilai Shu, Huawei Li

Sponsor: Eye & ENT Hospital of Fudan University

Protocol version number: 05, June 21, 2023

Signature

Date: June 21, 2023

Contents

1. Background 6

2. Research purpose 8

2.1 Primary purpose 8
2.2 Secondary purpose 8

3. Study design 8

4. Subject inclusion criteria, exclusion criteria, and suspension criteria 11

4.1 Inclusion Criteria 11
4.2 Exclusion Criteria 12
4.3 Suspension criteria 13

5. The number of cases required to achieve the intended purpose of the study based on statistical principles 14

6. The dosage form, dose, route of administration, method of administration, frequency of administration, course of treatment, and related regulations for combined medications, as well as instructions for packaging and labeling 14

6.1 General information 14
6.2 Administration Proposal 15
6.3 Surgical steps 16

7. Clinical and laboratory examinations to be performed, examination times, volume of collected blood samples or tissues, samples collection times and total amount of samples 17

7.1 Observation indicators 17
7.2 Tolerability and safety observation indicators 17
7.3 Preliminary curative effect observation indicators 18
7.4 Additional indicators 18
7.5 Biological sample collection, processing and testing 18

8. Registration and usage records, delivery and distribution methods and storage conditions of investigational drugs 19

9. Clinical studies, follow-up and measures to ensure subject compliance 19

10. Criteria for suspending the clinical study, regulations for ending the clinical study 19

11. Efficacy assessment criteria, including methods for evaluating parameters, observation time, recording and analysis 20

12. The storage procedure of subject's code, random number table, and case report form 20

13. The recording requirement of adverse event (AE) and the reporting, handling, follow-up visit, timing and outcome of serious AE 21

13.1 Definition 21
13.2 Severity of adverse event 22
13.3 Judgement of the causal relationship between adverse event and the investigational drug 23
13.4 Handling of adverse event 24
13.5 Recording of adverse event 24
13.6 Reporting of serious adverse event 25
13.7 Trail and follow-up visit of adverse event 26
13.8 Expected adverse reaction and handling 26

14. The establishment and maintenance of investigational drug code, the method of unblinding, and the rule of unblinding in the event of an emergency 30

15. Statistical analysis plan, definition and selection of statistical analysis data set 30

15.1 Definition and selection of analysis data set 30
15.2 Statistical analysis plan 31

16. Data management and information Confidentiality 31

17.1 Quality control (QC) 32
17.2 Quality assurance (QA) 33

18. Research relevant ethics 36

18.1 Examination and approval of trial documents from Ethics Committee before starting the trial 36
18.2 Subjects undergo the process of informed consent and acquire informed consent before starting the trial 36

19. Subject recruitment method and the process of acquiring informed consent 37

20. Expected progress and completion date of clinical trial 38

21. Follow-up and medical care after completing the trial 38

22. Responsibilities of the parties and other relevant regulation 38

List of Abbreviations

ABR	Auditory Brainstem Response
ADL	Activities of Daily Living
AAV	Adeno-associated Virus
ADR	Adverse Drug Reaction
AE	Adverse Event
ASSR	Auditory Steady State Response
AN	Auditory Neuropathy
ANSD	Auditory Neuropathy Spectrum Disorder
DFNB9	Autosomal Recessive Deafness 9
DLT	Dose-Limiting Toxicity
DPOAE	Distortion Product Otoacoustic Emission
EEAS	Efficacy Evaluable Analysis Set
EPS	Enrolled Patients Set
FDA	Food and Drug Administration
FAS	Full Analysis Set
GCP	Good Clinical Practice
IT-MAIS	Infant Toddler Meaningful Auditory Integration Scale
ITT	Intentionality Analysis
MSP	Mandarin Speech Perception
MAIS	Meaningful Auditory Integration Scale
PPS	Per-Protocol Set
PTA	Pure Tone Audiometry
QA	Quality Assurance
QC	Quality Control
SAS	Safety Analysis Set
SAE	Serious Adverse Event
SMP	Standard Management Procedure

SOP	Standard Operating Procedure
TEAE	Treatment-Emergent Adverse Event
UV	Ultraviolet
UADR	Unexpected Adverse Drug Reaction
WHO	World Health Organization

1. Background

Hearing loss is a common disease due to abnormalities in the auditory system. According to the World Health Organization (WHO), nearly 5% (about 466 million) of the population worldwide suffered from the disabling hearing loss, including 432 million adults and 34 million children, moreover, about one-third of elderly people over 65 own the disabling hearing loss as well. One billion people may be affected by the disease up to the middle of the century if hearing loss is not treated in time.[1] China is the country with the largest number of patients with hearing loss and 30,000 newborn infants are affected by this disease every year. Hereditary hearing loss accounts for about 60% of all hearing loss, additionally, there is no any medications used for the treatment of this disease. Auditory neuropathy (AN), also known as auditory neuropathy spectrum disorder (ANSD), it is one of the common otologic diseases that cause hearing loss, the prevalence of which is about 8.44% in children with profound hearing loss. Mutations to the *OTOF* gene (encoding the otoferlin), which causes autosomal recessive deafness 9 (DFNB9), are among the most common reasons for auditory neuropathy. The prevalence of *OTOF* is up to 41.2% in Chinese infants with AN.[2] However, there are no available therapeutic drugs for the treatment of the disease yet. Reconstruction of the auditory function by gene therapy is considered to be one of the most promising strategies to thoroughly treat hereditary deafness. Gene therapy refers to the delivery exogenous target gene to the patient with deafness by using delivery vector correct or compensate the abnormal genes, achieving a treatment of deafness.

The *OTOF* gene is recognized as among the most promising target in the field of gene therapy for deafness, which has great potential for clinical translation. *OTOF* mutation belongs to autosomal recessive inheritance, and can therefore be corrected by compensation of normal proteins. For the $Otof^{-/-}$ model mice, in recent years both groups in Germany and America have used dual AAV to deliver normal murine-derived Otof into the hair cells of mice, which significantly improved hearing of the model for at least 20 weeks.[3,4] The Akouos and Decibel companies in America firstly made the clinical protocols for the *OTOF* gene therapy system, which was approved by the FDA and was scheduled to be applied in patients in 2022. The highest prevalence of deafness in Chinese, there are no any clinical investigations for gene therapy of deafness yet, so it is of great public health importance to accelerate the development of drugs for treatment of deafness.

Our group is invariably committed to treating deafness by gene therapy and has developed an

effective and safe *OTOF* gene therapy system for DFNB9 mouse models. The therapy system used the adeno-associated virus vector (AAV) to deliver the normal human-derived *OTOF* gene into the inner ear of *Otof*$^{-/-}$ mice and significantly improved their auditory function. In addition, the *OTOF* gene therapy system was delivered into the inner ear followed by a comprehensive evaluation of its efficacy and safety through comparing different routes of administration, testing the hearing phenotypes of the animal, and assessing the toxicological risks of the drug. Based on our previous studies, the present project intends to conduct an investigator-initiated clinical trial to obtain a clinically available *OTOF* gene therapy drug, which will fill the domestic gap of clinical translation of gene therapy drugs for genetic deafness in China and will enable DFNB9 patients to obtain benefits.

The *OTOF* gene contains 48 exons and encodes the otoferlin which consists of 1997 amino acids, which mainly expresses in inner hair cells. The length of the *OTOF* sequence is approximately 6 kb, which together with the gene regulatory elements is longer than 7.5 kb, so it is difficult to package *OTOF* into a single AAV (~4.7 kb). Therefore, the present project uses dual AAV strategies to achieve in vivo high-efficient recombination of the *OTOF* sequence and to express functional otoferlin; also mouse hair cells will be high-efficiently and safely repaired by the inner ear hair cell-specific promoter, thereby expecting to restore auditory function of patients. Compared to previous reports, we intended to use human-derived *OTOF* and a specific-targeted promoter, which is characterized by higher safety and better tissue specificity.

AAV is the most commonly used delivery vector for gene therapy. Currently, three AAV-based drugs have been listed, including Glybera, Luxturna, and Zolgensma for the treatment of lipoprotein esterase deficiency, Leber congenital amaurosis, and spinal muscular atrophy, respectively. In addition, there are hundreds of AAV-related gene therapy drugs in the development of clinical pipelines. In the present research, AAV1 was used as the delivery vector due to its high usage frequency and proven manufacturing process. Glybera, the first listed gene therapy drug, uses AAV1 as its delivery vector. The pipelines developed by the AAV1 vector have been applied in various diseases, including muscular dystrophy, liver disease, hemophilia, viral infections, heart failure, and Huntington's chorea. Also, AAV1 is able to efficiently infect the inner ear hair cells of the target tissue in this project. The segmental package of the target gene using dual AAV vectors is a solution to break the upper limit of single vector packaging.

2. Research purpose

2.1 Primary purpose

To evaluate the safety and tolerability of RRG-003 injection in children with DFNB9 congenital hearing loss.

2.2 Secondary purpose

To evaluate the efficacy of escalating dose injections of RRG-003 in children with DFNB9 congenital hearing loss.

3. Study design

Study type: Interventional

Allocation and masking: non-randomized, open label

Intervention model: single arm, sequential assignment

This is a single-arm, open-label, sequential-assignment, non-randomized trial with dose ascending exploration. Considering the limited number of patients with rare diseases of Otoferlin mutations, and minimizing exposure to the risk of ineffective or low effective dose, the study design of investigational drug is as the following doses escalating protocol:

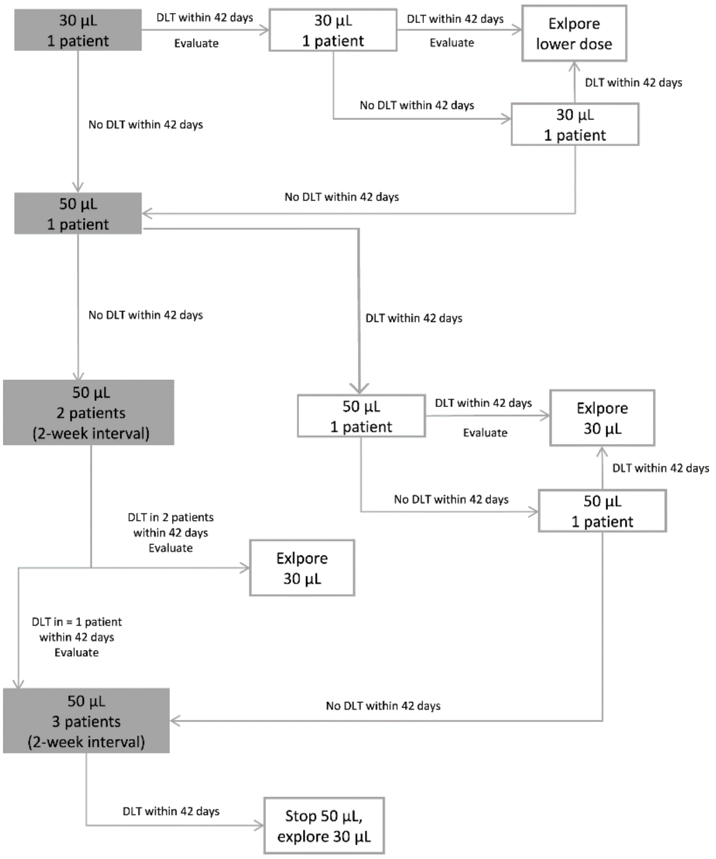

For participants who satisfy the inclusion criteria will be enrolled into this trial from with the drug concentration low dose to high dose. To begin with, equally volume mixed AAV-OTOF-N and AAV-OTOF-C drug administrate through ear canal to infuse cochlea with a micropump.

The first participant is enrolled in the dose group of 30 μL (9E11 vg/ear) with single-dose administration. If dose limiting toxicity (DLT) is not observed within 42 days after administration, the next participant can be enrolled into the dose group of 50 μL (1.5E12 vg/ear). If DLT is observed in the first participant during the 42-day DLT evaluation period, the second participant should be enrolled in the dose group of 30 μL after the risk evaluation of the investigators. If DLT is not observed in the second participant during the 42-day DLT evaluation period, the third participant can be enrolled in 30 μL group after the risk evaluation of the investigators. If DLTs are both observed at the first and the second participants within 42 days, the investigator can enroll one participant in the lower dose group or terminate the study after cautious evaluation. While the investigator is evaluating the safety of 50 μL, no more than 3 cases can be extended according to the clinical risks/benefits in the group of 30 μL dose.

The next dosage group is 50 μL (1.5E12 vg/ear). If there is no DLT observed during the 42-day DLT evaluation period after the first subject receiving a single dose, the second and the third participants can be enrolled in this dose group (the second and the third case are enrolled 14 days apart); If the number of DLT is less than or equal to 1/3 of the number of enrolled people in 50 μL dose group, no more than 3 participants can be enrolled in this dose group (the enrollment interval is 14 days); If there is one case of DLT observed in the second or third participants during the 42-day DLT evaluation period, it is necessary to enroll 3 more participants to determine whether the 50 μL dose is safe and tolerable. If DLT observed in the first participant during the 42-day DLT evaluation period, the second participant will be enrolled and received the injection after the risk assessment form investigators; However, if there is no DLT observed in the 42-day DLT evaluation period of the second participant, the third participant will be enrolled in this dose group after the risk assessment from investigator, and 3 more participants are required to be enrolled in the group of 50 μL dose (interval 14 days) for DLT evaluation. If 3-5 participants are evaluated without DLT events or 6 participants evaluated with ≤1 DLT event in this dosage group, investigators can carry out extended trials following comprehensive evaluation. During the period of expanding study, the investigator may enroll more participants in 30 μL or 50 μL dosage group for single injection or

double injection (including bilateral ear administration or repeated administration in single ear), or explore the administration of 70 μL does. If the number of participants is not less than 3 and no DLT is observed within 42 days, the investigator can explore double injection of administration in the 70 μL dose group. Once the number of participants with DLT in this dose group is more than 1, the exploration of this dose group should be stopped; the investigator could return to previous does group after the full evaluation.

The results of first participant in each dose group should be promptly reported to the ethics committee. During dose escalation among groups, investigators can perform expanding study in next dose group or previous dose group, based on the assessment of clinical risk and benefit. Investigators should apply in advance to adjust the number of participants strictly based on the study results. In appropriate circumstances, investigators may terminate the clinical trial in either dose group.

3.1 Dose limiting toxicity (DLT)

Dose limiting toxicity (DLT) is evaluated within 42 days after administration of RRG-003 according to Common Terminology Criteria for Adverse Events, NCI-CTCAE V5.0. DLT is defined as hematologic toxicity ≥ grade 4, nonhematologic toxicity ≥ grade 3, or aural toxicity in grade 2 or 3 within 6 weeks. Aural toxicity was listed as follow:

Adverse Events	Grade 2 ear adverse events	Grade 3 ear adverse events
Ear pain	Moderate pain; limiting instrumental Activities of Daily Living (ADL)	Severe pain; limiting self care ADL
External ear pain	Moderate pain; limiting instrumental ADL	Severe pain; limiting self care ADL
Hearing impaired (Pediatric)	Pediatric (on a 1, 2, 3, 4, 6, and 8 kHz audiogram): Threshold shift >20 dB at 4 kHz in at least one ear	Pediatric (on a 1, 2, 3, 4, 6, and 8 kHz audiogram): Hearing loss sufficient to indicate therapeutic

		intervention, including hearing aids; threshold shift >20 dB at 2 to < 4 kHz in at least one ear
Middle ear inflammation	Serous otitis, medical intervention indicated	Mastoiditis; necrosis of canal soft tissue or bone
Tinnitus	Moderate pain; limiting instrumental ADL	Severe pain; limiting self care ADL
Vertigo	Moderate pain; limiting instrumental ADL	Severe pain; limiting self care ADL
Vestibular disorder	Moderate pain; limiting instrumental ADL	Severe pain; limiting self care ADL
Ear and labyrinth disorders-Other, specify	Moderate; minimal, local or noninvasive intervention indicated; limiting age-appropriate instrumental ADL	Severe or medically significant but not immediately life-threatening; hospitalization or prolongation of existing hospitalization indicated; limiting self-care ADL
Otitis externa	Oral intervention indicated (e.g., antibiotic, antifungal, or antiviral)	IV antibiotic, antifungal, or antiviral intervention indicated; invasive intervention indicated
Otitis media	Localized; oral intervention indicated (e.g., antibiotic, antifungal, or antiviral)	IV antibiotic, antifungal, or antiviral intervention indicated; invasive intervention indicated

4. Subject inclusion criteria, exclusion criteria, and suspension criteria

4.1 Inclusion Criteria

8. Participants and their guardians can fully understand and voluntarily sign the informed consent of this study, willing to cooperate with follow-up at the specified time points in the trial.
9. Be able to communicate well with the researchers and comply with the requirements under the help of the guardian. For young children without mature language skills, they could cooperate and comply with the requirements under the help of guardians.
10. A proper understanding of the trial and an appropriate expectation of the benefit.
11. 1-18 years old, gender is not limited.
12. A diagnosis of DFNB9 congenital deafness was determined based on the clinical symptoms, and gene mutation analysis for the presence of either *OTOF* homozygous or bi-allelic mutations in *OTOF*.
13. Audiology inclusion criteria: Severe to complete hearing loss (≥65 dB).
14. Satisfy the requirements for ontological surgery: conditions such as middle-inner ear deformity, vestibular-cochlear nerve development abnormality, and ear inflammation, etc. are excluded through CT scan and/or MRI scan within 3 months or during screening.

4.2 Exclusion Criteria

16. Gene analysis does not suggest any *OTOF mutation*, or concomitant with other gene mutations causing hearing loss.
17. Other types of deafness that are not suitable for otological surgery, such as conductive deafness, mixed deafness, malformation syndrome caused by middle - inner ear dysplasia or malformed, abnormalities of vestibular nerve or cochlear nerve through CT/MRI scan within 3 months.
18. Pre-existing ontological diseases that may interfere with the interpretation of study endpoints, such as: acute-chronic otitis media, Meniere's disease, acoustic neuroma or unrecovered sudden deafness, etc.
19. A history of substance abuse, any ototoxic drug treatment (such as aminoglycosides, cisplatin, loop diuretics, etc.) within 6 months, antiviral or immunization therapy within 3 months, or vaccination within 1 month.
20. A history of complex immunodeficiency, or organ transplantation.
21. Patients with severe systemic disease or active bacterial or viral infection, such as pulmonary tuberculosis, active hepatitis B or C infection, active herpes zoster infection, pancreatitis, renal

failure, or gastrointestinal ulcers.

22. Patients with contraindications to surgery or anesthesia certified by the surgeon, anesthesiologist or designee, such as an allergy to the study medication, cardiovascular or cerebrovascular accident which occurred within the past 6 months, including myocardial infarction, heart failure, angina pectoris, cerebrovascular accident or transient ischemic attack.
23. Currently participating in or planning to participate in another clinical trial involving a drug or device within 1 year, or within 5 half-lives after the last dosing in another clinical trial.
24. Bilateral ear implants (e.g., cochlear implants).
25. With > 1:2000 neutralizing antibodies against AAV1 capsid.
26. Other severe congenital diseases.
27. A clear history of neurological or psychiatric disorders, including epilepsy or dementia.
28. Patients who require long-term use of anticoagulants is required.
29. A history of radiotherapy and chemotherapy.
30. Other conditions that investigators do not consider appropriate for participating in the present clinical study.

4.3 Suspension criteria

6. Unsuccessful administration. Drug administration was not completed according to the protocol.
7. The participants withdraw from the test or loss of follow-up
8. Allergic reaction or serious adverse events. Due to the obvious abnormalities of drug safety related laboratory indicators or adverse events (such as severe allergic reactions) during administration process, the investigator and/or the sponsor considers that it is not appropriate to continue injecting drugs based on ethical consideration. People who need rescue due to serious adverse events. Complicated by other serious diseases during the trial period, and the investigator judges that it is not appropriate to continue to participate in the trial;
9. During the 6-week evaluation period, the original condition progresses or other treatments are required: if no treatment measures are taken temporarily, they can continue to participate in the trial or withdraw from the trial at the discretion of the investigator; if other treatments are required, the trial should be withdrawn;
10. Serious violation of trial protocol. The participants or the participants' guardian refused to

follow-up or keep serum samples, and the combined use of prohibited drugs that significantly exceeds the dosage may bring risks; the required inspection items are not complete as planned, resulting in an inability to assess safety and efficacy; the informed consent process is incomplete or there is no informed consent procedure; the participant's guardian did not sign the informed consent or temporarily withdrew the informed consent.

The original data and documents of the selected or enrolled subjects should retain for retention and intentionality analysis (ITT), whether they drop out or not.

5. The number of cases required to achieve the intended purpose of the study based on statistical principles

According to the description above, the total number of effective cases was 4-12. Two to three dose groups were set for administration, and the exploration dose was set as 30 μL (9.0E11 vg/ear), 50 μL (1.5E12 vg/ear), 70 μL (2.1E12 vg/ear). The exploration age was 1-18 years old. An effective case was defined as a subject who was given drug in compliance with the protocol and was observed for 6 weeks at least. The actual number of subjects can be adjusted according to trial requirements.

6. The dosage form, dose, route of administration, method of administration, frequency of administration, course of treatment, and related regulations for combined medications, as well as instructions for packaging and labeling

6.1 General information

- **Generic name:** RRG-003 ear injection
- **Product name:** not available
- **Study code:** RRG-003
- **Active ingredients:** Two AAV1 gene therapy vectors, AAV-OTOF-N and AAV-OTOF-C, carrying the N-terminal and C-terminal coding sequence of human otoferlin, respectively.
- **Characteristics:** The contents of this product are colorless transparent or slightly yellow liquid.
- **Dose:** 30-70 μL/ear.
- **Administration route：** Injection through the round window following the stapes punched; Other more suitable minimally invasive inner ear injection methods could also be explored.

- **Administration method:** ***
- **Number of doses:** one time or two times.
- **Treatment course:** one time or two times.
- **Specification:** AAV-OTOF-N and AAV-OTOF-C are***
- **Lot number:** To be determined.
- **Validity period (tentative):** 24 months.
- **Storage condition:** ≤-65°C.
- **Entrusted manufacturer:** Shanghai Refreshgene Therapeutics Co., LTD.
- **Manufacturer:** Guangzhou PackGene Biotechnology Co., LTD.

RRG-003 was packaged in small boxes, each containing one vial of AAV-OTOF-N and one vial of AAV-OTOF-C. Preparation buffer and blank penicillin vials are also provided for mixing prior to clinical administration.

All packaging and drug labels are marked "for clinical trial use only". If there are quality problems such as breakage and discoloration, another box of drugs could be taken and the sponsor would be contacted in time to reissue the drugs.

6.2 Administration Proposal

6.2.1 Preoperative care

5. Clean the external auditory canal thoroughly before surgery.
6. Daily ultraviolet (UV) disinfection in the ward.
7. Nurses participate in the preoperative discussions to understand the surgical methods, objectives, methods, procedures, required equipment, and nursing requirements.
8. Familiar with the possible postoperative complications, such as infection, dizziness, facial paralysis, bleeding, labyrinthitis, and wound nonhealing, along with the observation and report in time.

6.2.2 Steroids Prophylaxis

Dexamethasone intravenous infusion usage based on the body weight, starting on the -3 day and lasts until the 5th day of the post-operation, to reduce the postoperative inflammation and immune response to AAV medications.

6.3 Surgical steps

***

Postoperative care

6. Before waking up from general anesthesia, the patient was lay down on the pillow with the head turned to one side. After waking up, take the supine position and do not press the injected ear. Do not move the head excessively. According to the doctor's advice, give ECG monitoring (closely observe the changes of vital signs), oxygen inhalation, and blood oxygen saturation monitoring (SPO_2 above 90%).
7. Intravenous injection of third-generation cephalosporin antibiotics start on the day of post-operation lasts for 5 days to prevent infection. Glucocorticoids could be used appropriately if accompanied by obvious acute dizziness.
8. Observation and nursing of whole brain syndrome. Closely observe the changes of vital signs, and continuously monitor the patient's consciousness, pupil, blood pressure, pulse, and respiration after waking up from general anesthesia. If the patient vomits, attention should be paid to distinguish whether it is a reaction after general anesthesia or an intracranial complication. If the blood pressure rises progressively, the pulse is slow and powerful, the breathing is deep and fast, the body temperature rises, and headaches are accompanied by changes in consciousness and pupils, one should be alert to the occurrence of intracranial complications and immediately report to the doctor for treatment.
9. Observation and nursing of local wound conditions. Observe whether the ear dressing oozed blood, keep the external ear canal clean, and prevent sewage from entering the ear. Do not blow the nose hard, and wipe it off gently to prevent backflow to the middle ear causing infection.
10. Observation and nursing of facial nerve function. Pay attention to whether there is injury of the facial nerve and chorda tympani nerve, focusing on observing whether there are facial paralysis manifestations such as crooked mouth corners, inability to close eyelid, reduction or disappearance of forehead lines, and taste alteration. Glucocorticoids, neurotrophins and physiotherapy should be applied once facial paralysis occurs.

7. Clinical and laboratory examinations to be performed, examination times, volume of collected blood samples or tissues, samples collection times and total amount of samples

Timeline/Procedure	Baseline	Day											Week					
		-3	-2	-1	0	1	2	3	4	5	6	7 (± 1 day)	2 (± 3 days)	4 (± 5 days)	6 (± 1 week)	13 (± 10 days)	26 (± 2 weeks)	52 (± 4 weeks)
Informed consent	X																	
Selected/excluded subjects	X																	
Next generation sequencing	X																	
Demography data	X																	
History of alcohol and drug dependence	X																	
Medical history/treatment history/family histor	X													X	X	X		X
Physical examination	X													X	X	X		X
Vital signs	X		X		X	X	X	X	X	X	X	X	X	X	X			X
Urinalysis	X		X					X				X	X	X	X	X		
Blood collection	X		X					X				X	X	X	X	X	Δ	X
Blood routine	X		X					X				X	X	X	X	X		
Serum Biochemistry	X		X					X				X	X	X	X	X		
Coagulation function	X		X					X				X	X	X	X	X		
Virology examination (HIV, HBV, HCV)	X																	
Circulating blood qPCR	X											X						
ELISpot	X														X	X		
Serum anti AAV1 antibody	X							Δ					X		X	X	Δ	X
12 lead electrocardiogram (ECG)	X		X												X			
Chest X-ray examination	X																	
MRI (ear, vestibule, brain)	X														X			X
Middle Inner Ear CT	X																	
Pure tone audiometry	X													X	X	X	X	X
ABR	X													X	X	X	X	X
DPOAE	X													X	X	X	X	X
ASSR	X													X	X	X	X	X
Speech recognition	X															X	X	X
Vestibular function	X													X	X	Δ	Δ	Δ
Otoscopy examination	X												X	X	X			X
Ear care					X	X	X	X		X		X						
Inner ear injection administration					X													
dexamethasone		X	X	X	X	X	X	X	X	X								
Adverse event evaluation	X					X	X					X	X	X	X	X	X	X
Near infrared functional imaging	Δ													Δ	Δ	Δ	Δ	Δ
electroencephalogram	Δ													Δ	Δ	Δ	Δ	Δ
Music testing	Δ													Δ	Δ	Δ	Δ	Δ
Growth and Development Scale	Δ															Δ	Δ	Δ

Note: Δ means optional text; if the subject has a cochlear implant in one ear prior to the therapy of the drug, the audiology examination could be performed in injected ear or both ears after the operation. Investigator can adjust the inspection items appropriately according to the participant's condition.

7.1 Observation indicators

7.1.1 Demographic information: age, gender, height and weight;

7.1.2 Present history, past history and family history: medical history, previous treatment history, other disease history and family history of the DFNB9 children;

7.1.3 Disease observation indicators (screening period): Gene sequencing, otoscopy examination, ABR (Auditory Brainstem Response), DPOAE (Distortion Product Otoacoustic Emission), PTA (pure tone audiometry), ASSR (Auditory Steady State Response), speech recognition rate (for infants and toddlers who cannot cooperate, methods of "family game", video observation and questionnaire survey can be used for evaluation), MRI (internal auditory canal), CT (middle inner ear) and vestibular function examination (for those who can cooperate).

7.2 Tolerability and safety observation indicators

7.2.1 Vital signs: heart rate, respiration, body temperature, blood pressure;

7.2.2 Physical examination: major organs and systems;

7.2.3 Neurological examination: MRI;

7.2.4 Laboratory tests: blood biochemistry, coagulation function, blood and urine routine, anti-AAV

neutralizing antibody, ELISpot, blood qPCR for AAV shedding detection;

7.2.5 Electrocardiography: standard 12-lead ECG;

7.2.6 Imaging examination: CT or MRI;

7.2.7 Specialist medical examination: external ear and ear canal, tympanic membrane, tympanic chamber, mastoid process

7.2.8 Vestibular function assessment: if cooperate

7.2.9 Adverse events.

7.3 Preliminary curative effect observation indicators

7.3.1 Pure tone audiometry test (if cooperate);

7.3.2 ABR test;

7.3.3 ASSR;

7.3.4 DPOAE;

7.3.5 Speech recognition: speech assessment methods include questionnaires and speech tests. The Infant toddler meaningful auditory integration scale (IT-MAIS), Auditory Performance Rating Scale (CAP) and Speech Intelligibility Rating Scale (SIR) were used in the questionnaire survey at the age of 1-3 years. Meaningful auditory integration scale (MAIS), CAP and SIR Were used in the questionnaire survey for children over 3 years old. Mandarin Speech Perception (MSP) by Fu Qianjie Fu was used for speech assessment in children older than 3 years old.

7.4 Additional indicators

7.4.1 Near-infrared light functional imaging: Optional exploration based on the actual situation and needs.

7.4.2 Electroencephalo-graph (EEG): Optional exploration based on the actual situation and needs.

7.4.3 Music test: Optional exploration based on the actual situation and needs.

7.4.4 Growth and development scales: Optional exploration based on the actual situation and needs.

7.5 Biological sample collection, processing and testing

7.5.1 Urine sample collection (urine routine);

7.5.2 Blood and serum collection (blood routine, blood biochemistry, coagulation function,

neutralizing antibody, ELISpot, circulating blood qPCR).

8. Registration and usage records, delivery and distribution methods and storage conditions of investigational drugs

RRG-003 should be refrigerated at ≤ -65°C, and kept, issued and dispensed by dedicated personnel. The Trial Drug Registration Form should be filled out, signed and confirmed by the drug administrator and investigator each time the drug is issued and returned. Store the trial drug in a refrigerator that only authorized personnel can open until it is used or returned to the trial drug provider. The investigator should ensure proper storage conditions and regularly check and verify the quantity and storage of the drug. All test drugs provided by the investigational drugs provider only be used for experimental research purposes and shall not be used for purposes other than those specified in this protocol. The investigator must undertake not to provide the investigational drugs to anyone not related to the trial.

9. Clinical studies, follow-up and measures to ensure subject compliance

In this study, patients will be taken care in hospital during the operation and per-operative periods. For guaranteeing the compliance during the surgical period, the inspection would be carried out. Since this clinical study is dosed by surge, there are no compliance issues in terms of medication administration. During the follow-up phase, patients will be contacted for checkups at the hospital, and testing accommodation and testing costs during this period are covered by a dedicated fee. If the patients encounter special circumstances that prevent them from coming to our hospital for examination, they will be contacted to go to the local hospital for examination.

10. Criteria for suspending the clinical study, regulations for ending the clinical study

1) The principal investigator believes that the drug own potential safety risks and continuing the trial is not conducive to protecting the rights and interests of the subjects;

2) There are major defects in the trial protocol and the trial cannot be carried out smoothly;

3) The sponsor requests termination；

4) The ethics committee or drug regulatory authority requires termination of the trial;

5) Other cases that the investigator considers it is not appropriate to continue the study or considers it difficult to proceed the study.

11. Efficacy assessment criteria, including methods for evaluating parameters, observation time, recording and analysis

Focusing on the purpose of the study, involving the efficacy assessment must have evaluation indicators or observation indicators, including primary and secondary evaluation indicators. At the same time, corresponding evaluation standards should be established for these indicators.

Primary indicators: tolerability (dose-limiting toxicity, DLT) at week 6.

Secondary indicators: safety, preliminary efficacy.

Initial efficacy:

-Pure tone test: change in pure tone audiometric thresholds at each follow-up evaluation point compared to the pre-treatment baseline: a stable reduction of 20 dB at any one of the PTA audiometric frequencies or a stable reduction of 10 dB at any two audiometric frequencies is considered clinically significant;

-ABR test: change in ABR thresholds at each frequency at each follow-up evaluation point compared to the pre-treatment baseline;

-ASSR: change in ASSR thresholds at each frequency at each follow-up evaluation point compared to the pre-treatment baseline;

-Speech recognition: change in speech recognition rate at each follow-up evaluation point compared to the pre-treatment baseline. Speech assessment takes the form of questionnaires aged 1-3 years by using IT-MAIS, CAP, SIR. Questionnaires for children aged 3 years old and older by using MAIS, CAP, SIR. The speech test for children over 3 years old.

12. The storage procedure of subject's code, random number table, and case report form

Each participant will be given a unique screening number, and enrolled participants will be given a unique enrollment number. The trial will be conducted using single-arm sequential enrollment without randomization. Beginning with screening, a dedicated case report is established for each subject. Relevant storage procedures are handled in accordance with the management regulations of clinical research institutions.

13. The recording requirement of adverse event (AE) and the reporting, handling, follow-up visit, timing and outcome of serious AE

13.1 Definition

13.1.1 Adverse Event (AE)

AE is any adverse medical event observed after the use of a trial drug in the subject, regardless of whether it is treatment related.

The difference between an AE and an adverse reaction: an adverse reaction means that there is a causal relationship between therapeutic drug and adverse reaction; an adverse event indicates that the causal relationship between therapeutic drug and adverse event is not confirmed, requiring further analysis and evaluation.

13.1.2 Serious Adverse Event (SAE)

SAEs are any undesirable clinical events occurring at any dose, such as death, life-threatening, hospitalization or prolongation of hospitalization to treat AEs, persistent or significant disability, congenital anomaly or birth defect.

An adverse event should be reported to the sponsor by the research center within 24 hours if the adverse event meets one of the following criteria and is diagnosed as serious adverse event:

Death: the death of subject resulting from adverse event;

Life-threatening: in the opinion of the investigator, an adverse event is likely to result in the immediate death of the subject without medical intervention, excluding the adverse event that occurs more seriously, and already results in death;

Hospitalization: admission to the hospital that is a result of the adverse event, not including emergency or outpatient visit;

Prolongation of hospitalization: adverse event occurs during the subject's hospitalization, which leads to the prolongation of hospitalization for AE therapy;

Persistent or significant loss of function or disability: the subject is unable to perform normal daily activities. Loss of function does not include relatively minor medical events, such as headache, nausea, emesis, diarrhea, influenza and accidental trauma (e.g., sprained ankle);

Important medical event: the event may not result in immediate death, life-threatening or hospitalization, but the important medical event may jeopardize the subject and require medical or surgical intervention to prevent any of the events above (i.e., death, life-threatening, hospitalization

or prolongation of hospitalization, etc.). For example, allergic bronchospasm requiring treatment in the emergency room or at home, convulsions that do not require hospitalization, cachexia, drug dependence or substance abuse, etc.

The hospitalization due to the progression of the original disease is not considered as serious adverse event. The hospitalization resulting from elective surgery and examination or other treatments that are scheduled prior to entering into this trial, or social reasons.

13.1.3 Adverse Drug Reaction (ADR)

For the drug not yet approved for marketing, an adverse reaction is an uncomfortable and unexpected reaction associated with the administration of drug at any dose. There is a causal relationship, or at least a reasonable possibility, between the adverse reaction and the administration of the drug.

13.1.4 Unexpected Adverse Drug Reaction (UADR)

An unexpected adverse drug reaction is an adverse reaction, the nature or severity of which is not consistent with the current product information (e.g., expected adverse events not listed in the investigator's brochure).

The "expected" or "unexpected" adverse reaction is distinguished on the basis of the events observed previously and cannot be predicted from the pharmacological property of drug: ① for drugs not yet approved for marketing, the investigator's brochure is used to determine whether the nature or severity of adverse reaction is consistent with the information described in the investigator's brochure; ② new clinically meaningful information on the nature and severity of a known and documented serious adverse event is also classified as an unexpected adverse reaction.

13.2 Severity of adverse event

The severity of adverse event, not associated with ear, should be judged according to the NCI-CTCAE v5.0.

The severity of anticipated adverse events in the ear is defined in terms of mild, moderate, severe, and life-threatening:

Mild/Grade 1: discomfort that not interfere with daily activities; children at 8 kHz with a threshold shift of 20 dB hearing loss; sensorineural hearing loss above 4 kHz in at least one ear.

Moderate/Grade 2: discomfort interfering with daily activities; children at 4 kHz with >20 dB threshold shift in one ear.

Severe/Grade 3: inability to work or perform daily activities; hearing loss requiring therapeutic intervention, including hearing aids; threshold shift of 20 dB in at least one ear at ≥2 kHz.

Life-threatening /Grade 4: Adverse event may lead to death; cochlear implant; threshold shift of >40 dB of hearing loss, sensorineural hearing loss at >2 kHz.

Death/Grade 5.

13.3 Judgement of the causal relationship between adverse event and the investigational drug

At present, the causal relationship between adverse event and medication administration is usually classified into 5 cases in China and internationally:

a) Definite Related: adverse event occurs explained by drug administration;

b) Probably Related: adverse event occurrence probably highly related to drug administration;

c) Possibly Related: adverse event occurrence possibly related to drug administration;

d) Unlikely to be Related: adverse event occurs more likely to be related to another factor;

e) Not Related: adverse event due to other significant factors.

Items ① to ③ above should be recorded as adverse drug reactions. Incidence of adverse reaction = number of (① + ② + ③) cases/number of cases in safety data set × 100%.

The causal relationship between adverse event and medication administration

	① Definite Related	② Probably Related	③ Possibly Related	④ Unlikely to be Related	⑤Not Related
Temporal relationship: a reasonable temporal relationship between drug administration and the occurrence of reaction / event	+	+	+	+	-
Known: the reaction that is consistent with known adverse reaction of drug	+	+	+	-	-
Dose-response relationship:	+	+	±	±	-

disappearance or reduction of reaction/event after drug withdrawal or dose reduction					
Recurrence: reaction/even that reoccurs after a rechallenge	+	?	?	?	-
Explainable: reaction/event that is not explained by the effect of the concomitant medications, the progression of subject's condition, the effect of other treatments.	+	+	-	±	-

13.4 Handling of adverse event

In the event of an adverse event, the investigator decides whether to terminate the trial based on the subject's condition; in the event of a serious adverse event, the investigator must immediately conduct the correct treatment or rescue treatment to protect the subject's safety.

The main measures to be taken in the event of an adverse event include:

1) observation only;

2) dosage adjustment of the investigational drug or dose interruption;

3) withdrawal of the investigational drug;

4) treatment of concomitant medications;

5) non-pharmacological therapies;

6) hospitalization or prolongation of hospitalization.

13.5 Recording of adverse event

Serious adverse event occurring after acquirement of Informed Consent Form and before the end of the trial; all adverse events need to be recorded in the corresponding section of the CRF after acquirement of Informed Consent Form until subject out, regardless of whether the adverse event is related to the investigational drug, unless the subject withdraws the informed consent and follow-up visit cannot be performed.

All adverse events should be recorded in concise medical terms include: ① a description of adverse event and all associated symptoms; ② the time of occurrence and the duration of adverse event; ③ the severity of the adverse event; ④ the examinations and treatments performed due to the adverse event; ⑤ the final outcome of adverse event; ⑥ judge whether the adverse event was related to the investigational drug (if it could be judged).

13.6 Reporting of serious adverse event

If all serious adverse events are judged as serious adverse events, the investigator should report to the sponsor and the drug regulatory department within 24 hours, regardless of whether the adverse events are related to the investigational drug or trial operations. The sponsor has responsibility for reporting the serious adverse event to the Department of Drug Administration and other clinical trial centers for the same drug in accordance with the regulations. Unless other requirement from the local regulatory authority and other documentation from the Ethics Committee, the investigator should also report the serious adverse event to the Ethics Committee that approved the protocol in accordance with the regulations.

The investigator fills in the Serious Adverse Event Report Form with a detailed description of the course of adverse event, the diagnosis, the treatment given, and the possible relationship to the drug. Follow-up report should be completed and reported to all parties according to the initial reporting procedures if the diagnosis is unclear or changed after it has been reported, or there is an important change in the subject's condition (e.g., the adverse event progresses to death from prolongation of hospitalization).

Serious adverse events should be sent by the investigator to the sponsor or the supervisor by fax:

Fax number:

Contact for the sponsor:

Phone number:

The sponsor should report the serious adverse event to the following department within 24 hours after receiving the Serious Adverse Event Report Form from the investigator:

China Food and Drug Administration, Department of Research Supervision:

Shanghai Food and Drug Administration, Registry:

When the investigator and sponsor report serious adverse event to related departments, the

relevant correspondence records should be stored, such as fax-sending reports, handover documentation, telephone records, etc.

13.7 Trail and follow-up visit of adverse event

All adverse events should be followed up with detailed documentation of handling process and outcome, until they are appropriately resolved or stable. Any laboratory test results that are still abnormal at the end of the trial should be followed up by the investigator until they return to be normal or are clinically stable. According to the severity of adverse event, the distance of the subjects' residence from the hospital, and the medical specialty of the trial center, various forms of follow-up visit can be selected, such as inpatient, outpatient, home visit, telephone, and communication. The outcomes of all adverse events should be recorded in the CRF timely, and if the adverse event has not recovered by the end of the trial, the investigator should continue to follow the subject and record the relevant results in the medical record.

All serious adverse events should be followed up until the events are fully resolved; and at least one follow-up or summary report should be provided to detailed describe the treatment given to the subject, the outcome of subject’s condition, the final diagnosis, and the relationship between final diagnosis and investigational drug, since the onset of the serious adverse events. The summary report of the serious adverse events should be reported to the sponsor and the Ethics Committee in accordance with the initial report procedures, and should be also reported to the Department of Drug Administration by the sponsor.

13.8 Expected adverse reaction and handling

13.8.1 Risk assessment of the trial

Based on the clinical trial data from systemic administration of AAV drug, the risk of serious adverse events associated with AAV drugs includes hepatotoxicity (elevated liver enzymes, liver failure, etc.), thrombocytopenia, hemolysis, anemia, acute kidney injury, and neurotoxicity found on MRI. The local administration of AAV drug in the clinical trial such as ophthalmic trial gives rise to few systemic toxicity, mainly local inflammation at the site of administration and systemic reactions due to hormone administration at the same time.

Based on the data from drug administration by injected into inner ear of patients with profound

deafness in clinical trials, adverse reactions may be associated with AAV drugs, injection procedures, concomitant corticosteroids or a combination of these procedures and products. The most common adverse reactions (>5%) in the ear include mild hearing loss, dizziness, lightheadedness, nausea, and vestibular dysfunction, etc. Other adverse reactions with an incidence of less than 5% include otalgia, ear bleeding, unilateral deafness, tinnitus, taste change due to the injury of chorda tympani nerve, otitis media, perilymphatic fistula, reparative granuloma and tympanic membrane perforation, facial palsy, inner ear infection and meningitis, and sinusitis, etc. Systemic adverse events include hepatic impairment (elevation of ALT, AST, etc.), fatigue, food allergies, seasonal allergies, atopic dermatitis, urticaria, etc. These are systemic events that may be caused by systemic administration of corticosteroids and anesthetic reactions.

In summary, this clinical trial may be exposed to risks caused by the above-mentioned adverse reactions and risk control will be performed during the clinical trial to ensure the safety of the subjects.

13.8.2 Treatment Precautions

In accordance with the regulations of NMPA's new GCP, patients who are ready to participate in the trial will be screened strictly according to the inclusion and exclusion criteria, avoiding to include patients who do not meet the criteria in the trial. To guarantee the safety of subjects, risk control will be carried out during the trial and is planned as follows:

(1) Multiple follow-up visits will be performed before dosing, during the treatment and thereafter, including subject's general condition, clinical signs and symptoms, any adverse events during discharge, medical physical examination, observation of injection site reactions and other adverse events, and otologic examinations. Otologic examinations include ear inflammation, ear infection, ear bleeding, pure tone test, speech recognition, ABR, DPOAE, vestibular assessment, etc. Laboratory tests include blood, urine, function of liver and kidney, blood electrolytes, coagulation. Monitor the occurrence of adverse events specially. The above contents include the safety indicators for RRG-003, aiming to control possible adverse drug reactions and risk factors during the trial.

(2) Monitor and control possible adverse drug reactions and risk factors during the trial by signs and symptoms, laboratory tests, and otologic imaging; and give advice on handing.

(3) The subject can withdraw from the trial at any time during any phase of the trial.

(4) The safety and efficacy data from this trial are regularly analyzed by the principal investigators and/or statisticians, who decide whether the trial should be continued.

(5) If the subject experiences an adverse event related to the investigational drug during the trial, the following principles need to be followed for handing of adverse event.

If an accident occurs during inner ear surgery, terminate injection promptly. And medical treatment should be conducted by the investigator;

Adverse events that do not meet the DLT during the observation of DLT are not treated generally;

After completion of DLT observation, adverse events should be handled actively;

Once adverse event happens in the subject, the investigator is responsible for making relevant medical decisions based on clinical practice to ensure that the subject is treated appropriately and the handling of AE should be documented.

(6) Risk of infection after inner ear drug administration

Nurse the subject carefully during the perioperative period of inner ear surgery; prevent infection with antibiotics and glucocorticoids after surgery. Contact the investigator promptly if subject fails to comply with medical advice or an accident occurs.

(7) Other toxic reactions: Symptomatic treatment is available. If blood tests and urine tests are abnormal, collect and detect samples again within 24 hours; and the corresponding treatment or therapy should be performed according to the test results.

(8) Risks associated with dexamethasone via intravenous injection:

Dexamethasone can reduce postoperative inflammation and decrease the immune response to AAV drugs, and is often used before and after ear surgery.

Common side effects of dexamethasone include increased appetite, stomach upset, nervousness, or restlessness. Less common but serious side effects include decreased or blurred vision, fluid retention, weight gain, increased blood sugar level, frequent urination, thirst, mood swings, confusion, and rash or urticaria. Other rare side effects include darkening or brightening of the skin, dizziness, facial flushing, hiccups, and increased sweating.

Dexamethasone may reduce resistance to the infection, which may lead to a sore throat, fever, sneezing or coughing.

Once side effects occur, discontinue dexamethasone immediately.

(9) General anesthesia:

Serious but very rare adverse events (negative side effects) associated with any types of anesthesia/surgery include seizures, coma, and death.

Rare but serious complications associated with general anesthesia include cardiac arrhythmias, increased or decreased blood pressure, transient high fever, rare reactions to drugs used in anesthesia, and airway obstruction.

The risk associated with a small amount of local anesthesia is low. Anesthetic injection is accompanied with a small risk of penetration of the eye or optic nerve, and the possibility of hemorrhage, which need to be treated if necessary.

(10) Risks associated with surgery:

1. Injury to ear ossicles such as injury to stapes, resulting in the dislocation of stapes; or even injury to oval window, leading to perilymphatic fistula.

2. Injury to jugular bulb, causing hemorrhage. In the event of hemorrhage, the ear canal should be filled with pressure to stop bleeding.

3. Facial paralysis, is usually caused by injury to the facial nerve or excessive strain on the chorda tympani nerve. If facial paralysis occurs, analyze the cause and perform facial nerve decompression surgery timely if necessary.

4. Labyrinthitis: nurse surgical cavity carefully after surgery; and use antibiotics and glucocorticoids that can cross the blood-brain barrier to prevent the spread of inflammation; persist with follow-up visit.

5. Post-operative infection, short or long-term ear discharge, perichondritis. Strictly grasp the surgical indications and contraindications before surgery and pay attention to aseptic operation during surgery. Dressing should be changed after surgery, and granulation in the surgical cavity should be treated in time to protect the new epithelium. Pay attention to drainage and removal of secretions.

6. Tinnitus.

7. Cerebrospinal fluid leak. Promptly repair it with mastoid cortex and temporalis fascia.

8. Taste change, usually caused by injury of chorda tympani nerve.

(11) Thrombocytopenia:

1. Hemostasis: in the event of hemorrhage, stop bleeding via antihemorrhagic, prevent internal bleeding, and then identify the cause.

2. Hormone therapy: In the event of mucous membrane or more extensive skin bleeding, treat the subject with adrenocortical hormone to control the condition and reduce the damage to the body. Use estrogen for a short time in an acute condition.

3. Blood transfusion: When a platelet count is less than 30*10^9/L with continuous bleeding, platelet transfusion should be performed in time to stop bleeding as soon as possible.

In conclusion, according to the risk control plan, the possible risks of RRG-003 during the trial are controlled, and suggestions are med for the treatment of possible adverse reactions, ensuring participants take drugs reasonably and safely.

14. The establishment and maintenance of investigational drug code, the method of unblinding, and the rule of unblinding in the event of an emergency

Not applicable.

15. Statistical analysis plan, definition and selection of statistical analysis data set

15.1 Definition and selection of analysis data set

Enrolled Patients Set (EPS): defined as all enrolled cases, with or without investigational drug. The data set is used to analyze data such as demography and baseline information, subject allocation, and protocol violation.

Safety Analysis Set (SAS): defined as all enrolled cases that use the investigational drug at least once. The data set is used to analyze safety data.

Efficacy Evaluable Analysis Set (EEAS): defined as all enrolled cases who had at least one efficacy assessment after receiving investigational drug. The dataset is used for analysis of preliminary efficacy data.

Full Analysis Set (FAS): Defined as all enrolled cases who receive investigational drug at least once and have at least one post-baseline efficacy assessment according to the intention-to-treat (ITT) principle. The dataset is used for efficacy analysis.

Per-Protocol Set (PPS): defined as all cases, incorporated in the full analysis set, who comply with the protocol, have good medication adherence, do not use prohibited drugs during the trial, and have a defined efficacy assessment in the trial. The dataset is used for efficacy analysis.

Statistical analysis methods

15.2 Statistical analysis plan

Descriptive statistics are primarily used to analyze trial results. In general, measurement data will list the number of observational cases, mean, standard deviation, median, quartile, maximum and minimum. Enumeration data will list frequency and relative frequency. Unless otherwise specified, all of statistical tests will perform two-tailed test with a 95% confidence interval. Statistical analysis will be performed in a version of SAS 9.4 or higher version.

Use descriptive statistics to describe the number and proportion of subjects entering each analysis set, the number and proportion of subjects completing the trial and withdrawing consent, and the reasons for withdrawing consent (and proportion of subjects) for each dosage group; to describe the baseline characteristic of each dosage group.

Use the Safety Analysis Set to summarize treatment-emergent adverse event (TEAE) and adverse reaction for each dosage group; the AEs, occurs after signing informed consent and before treatment, will be only tabulated based on per subject and not summarized. TEAE is defined as the adverse event that is not present before dosing and occurs within 28 days (inclusive) after administration. The severity of adverse event and adverse reaction will be graded based on the NCI-CTCAE v5.0. Adverse event and adverse reaction will further be summarized based on System Organ Class and Preferred Term.

Using the safety analysis set baseline data, post-dosage data, and change from baseline were summarized for each follow-up visit and each dosage group, according to safety data such as laboratory, vital signs, electrocardiogram, physical examination, and otologic examination. Shift tables will be used to describe change from baseline after each follow-up visit to determine whether each examination result was normal and clinically significant as categorical data.

Use Per-Protocol Set to summarize efficacy data at each efficacy evaluation point and plot the efficacy-time curve of each subject, the mean efficacy-time curve of each dosage group, and the mean efficacy-curve of all subjects, according to efficacy data change from baseline in each dosage group.

16. Data management and information Confidentiality

The investigator is responsible for data management in this trial.

The data on the eCRF is derived from the original medical records and completed by investigator

or the individual specified by investigator, ensuring the completeness and accuracy of the information. If there are errors that need to be corrected, the amendment should be made according to the eCRF completion instructions.

17. Quality control and quality assurance of clinical trial

17.1 Quality control (QC)

17.1.1 Qualification of research institute and investigator

The research institute should have relevant qualifications for conducting the clinical trial of medicinal product; and the facilities and conditions of the department conducting the trial should meet the requirements for carrying out clinical trial safely and effectively. The investigators should have the specialty, qualification and skill to conduct the trial and should be trained for GCP and this protocol. Prior to the start of the trial, the project director of the center should organize the investigators to study the protocol, and only those who are trained for the protocol can participate in the trial. The investigators participating in the trial should be relatively fixed, and those who join the trial during the trial should be trained first, and the clinical trial participator authorization form should be updated timely.

17.1.2 Laboratory quality control measures

The laboratory of trial center establishes standard operating procedures and quality control procedures for laboratory test index and provides appropriate certificate of qualification.

17.1.3 Guarantee compliance

Subject compliance: The process of informed consent and the signing of informed consent form are important aspects of ensuring subject compliance. The investigator should patiently explain informed consent to the subject's guardian, so that the subject's guardian can fully understand the content and process of trial, as well as his or her rights and obligations, like complying with medical prescriptions to take medication on time, follow-up visit on time and completing required clinical observations and physical and chemical examinations.

Investigator compliance: Investigators should strictly comply with the trial protocol and relevant regulations to carry out clinical trial. Their compliance is mainly reflected in the three main aspects, including selecting qualified subjects, controlling imposed factors, and observing and evaluating effect indicators. The specific contents are as follows.

a) Strictly comply with the trial protocol and regulations, with no serious violations of the protocol;

b) Select qualified subjects to participate in this trial and sign the informed consent form;

c) Strictly comply with the protocol to enroll subjects and perform trial observations and records;

d) Impose reasonable treatment on the subject. All effects, derived from imposed factors (including investigational drugs, concomitant medications) and other measures, can be measured, assessed, and judged with objective, credible, scientific and feasible criteria;

e) Handle, record and report adverse events in time, especially serious adverse events;

f) The trial strictly complies with the standard operating procedure (SOP);

g) Data management and statistical processing strictly comply with SOP.

17.2 Quality assurance (QA)

17.2.1 Good Clinical Practice and the Declaration of Helsinki

The Good Clinical Practice (GCP) and the Declaration of Helsinki are the legal support for maintaining the scientific and ethical principles of this clinical trial, as well as are the legal and regulatory guidelines for this clinical trial. Any person involved in this trial, especially the supervisors and inspectors appointed by the sponsor, has the right to correct any behavior and clinical treatment that violates the GCP and the Declaration of Helsinki. Especially when the rights of the subjects are not guaranteed and protected, the subjects have the right to terminate the clinical trial.

17.2.2 Trial protocol

The protocol is the guiding document of this clinical trial, guiding all investigators involved in this trial how to start and conduct the clinical trial, and it is also an important basis for data collection, recording, reporting and statistical analysis after the trial is completed. Therefore, the formulating a scientific and detailed trial protocol is not only a fundamental and important condition to ensure the success of the trial, but also an important document for quality control and quality assurance performed in the trial.

Before the start of the trial, the first investigators' meeting shall be held to discuss the protocol, the specific steps of the trial operation, the EDC data entry, sample collection methods in detail, The project director of the center, investigator and sponsor attend the meeting. If it is necessary to amend this protocol, either the investigator or the sponsor should proceed with the consent of the other

party. All amendment of the protocol will be issued by the sponsor and submitted by the investigator to the Ethics Committee for review or filing.

17.2.3 Statistical analysis plan

The statistical analysis plan is a data guarantee for quality management in the three main aspects: selecting qualified subjects, controlling imposed factors, and observing and evaluating effect indicators.

17.2.4 Informed consent and Informed Consent Form

Prior to the start of the clinical trial, the investigator must provide the subject's guardians with detailed information about the clinical trial, including the nature of the trial, the purpose of the trial, possible benefits and risks, alternative treatments available, and the subject's rights and obligations, etc. The clinical trial can only be carried out after the subject's guardian fully understand and agree, and sign the "Informed Consent Form". Informed Consent is one of trial documents to ensure the safety, rights and health of the subjects are maintained and protected during the trial process, i.e., any clinical trial is conducted on the premise that the health and rights of the subjects are not compromised.

17.2.5 Ethics Committee and Trial Document Review

As Informed Consent Form, the Ethics Committee is an important safeguard to protect the rights of subjects. In addition to reviewing the trial documents, the Ethics Committee audits protocol compliance and the protection and safeguarding of subjects' rights during the trial, in particular the treatment, trail and follow-up visit of subjects in the event of serious adverse events, ensuring that the trial is conducted in accordance with scientific and ethical principles.

Before the trial begins, the protocol should be discussed and revised by the project director and submitted to the Ethics Committee of the trial center for written examination and approval. The investigator should use a copy of the ethical approval as the basis for initiating and conducting this trial. The trial documents submitted to the Ethics Committee for examination and approval include:

1) Submitted and reviewed documentation checklist;

2) Ethics Review Application Form;

3) Clinical Trial Protocol;

4) Subject Informed Consent Form;

5) Recruitment Advertisement;

6) Curriculum Vitae of Principal Investigator;

7) Case Report Form;

8) Quality Verification Report of Investigational Drug;

9) Other relevant materials required by the Ethics Committee.

17.2.6 Supervisor’s Oversight and Sponsor’s Audit

The oversight of the trial is performed by the supervisor appointed by the sponsor to monitor the trial center according to the Standard Operating Procedure (SOP). The supervisor's oversight is one of the important guarantees to ensure the quality of the multicenter trial. The supervisor should communicate with the investigator and sponsor regularly.

The supervisor will assess the capabilities of each trial site and report issues related to facilities, technical equipment, or medical staff to the sponsor. During the trial, the supervisor will be responsible for monitoring that written informed consent is properly obtained from all subjects and that data records are accurately and completely documented. The supervisor also has the authority to compare data entered into the eCRF with the original data and to notify the investigator of errors or omissions. The supervisor will also ensure that the trial site adheres to the protocol, arranges and supplies the investigational drug, and ensures that the drug is kept in appropriate conditions.

The supervisor conducts monitoring visits in accordance with all relevant laws and regulations, and will conduct regular monitoring visits to each center beginning with the enrollment of subjects. After each visit to the investigator, whether by telephone or trial center visit, the supervisor shall submit a written report to the sponsor.

During the monitoring visit, the supervisor will compare the data entered into the eCRF with the original data file (Source Data Verification). This includes, but is not limited to, following items conducted 100% verification:

- Subject number;
- Obtaining informed consent from subjects;
- Subject eligibility (inclusion and exclusion criteria);
- Drug handling procedures;
- efficacy index;
- AE medical records.

The sponsor will also dispatch quality assurance personnel to audit the clinical trial. The scope of

audit includes the protection of subjects' rights, the implementation of the protocol and the work of investigators and supervisors, etc.

17.2.7 Establish and perfect the SMP and SOP of the center

The related research institutions should establish and improve regulations and Standard Management Procedure (SMP), and strengthen the training of GCP and related regulations for the investigators, ensuring that the trial is going well. Standard Operating Procedure (SOP) of the trial should be established and improved, and implemented and perfected during the trial, making the trial procedural, institutionalized, standardized and formatted.

18. Research relevant ethics

18.1 Examination and approval of trial documents from Ethics Committee before starting the trial

Before starting the trial, the protocol should be discussed and revised by project director of the trial center, and submitted to the Ethics Committee of the trial center for written examination and approval. The center should take ethical approval as the basis of initiation and implementation of the trial.

1) Submitted and reviewed documentation checklist;

2) Ethics Review Application Form;

3) Clinical Trial Protocol;

4) Subject Informed Consent Formtrial;

5) Recruitment Advertisement;

6) Curriculum Vitae of Principal Investigator;

7) Case Report Form;

8) Quality Verification Report of Investigational Drug;

9) Other relevant materials required by the Ethics Committee.

18.2 Subjects undergo the process of informed consent and acquire informed consent before starting the trial

After the official start of the trial and before the screening of the subjects, the investigators should provide informed consent to the guardian of each subject and explain the specific circumstances of

the clinical trial to them in detail. The guardian of the subject should sign the informed consent voluntarily on the premise that the guardian of the subject fully understands the trial process and agrees to participate in the trial.

18.3 Any AE occurred during the trial can be effectively managed and followed up

If any adverse event occurs during the trial, the investigator can decide whether to terminate the trial based on the condition. Once an adverse event occurs, the investigator will immediately implement appropriate treatment or rescue treatment to protect the safety of the subject.

All adverse events will be followed up until they are properly resolved or stabilized. According to the severity of adverse event, the distance of the subjects' residence and the hospital, and the medical specialty of the trial center, hospitalization, outpatient visit, home visit, telephone, and communication can be selected.

RRG-003 meets the requirement of IIT clinical trial in terms of quality standard, animal pharmacodynamics and safety.

This protocol follows the ethical principles of medical research involving human subjects outlined in the Declaration of Helsinki, is consistent with the ethical principles of medical research involving human subjects, and benefits all subjects as far as possible.

Although there is no guarantee that the result of this trial will be as expected, it is possible that the condition of subjects participating in the trial will improve. At the same time, all investigational drugs and related examinations required by the protocol are free.

19. Subject recruitment method and the process of acquiring informed consent

The researchers and their team members will release the information through advertisements posted in the hospital, inquiries during the consultation, and multimedia platforms such as WeChat. When the subjects see the recruitment advertisement and contact the hospital team, they will be fully informed about the risks and benefits of procedure before guardians decide whether to voluntarily participate in the trial. After the patient and his/her guardian confirm to participate in the clinical trial, we will issue informed consent form verbally and in writing one by one, and the patient and his/her guardian will confirm the informed consent one by one.

20. Expected progress and completion date of clinical trial

During the observation period of the administration, the subject is hospitalized on Day 1-Day 7 and undergoes physical examination, laboratory test, and otologic examination according to the trial flow chart during the follow-up visit on W2, W4, W6, W13, W26, and W52. Safety, tolerability, and adverse events are evaluated on Days 1, Day 2, Day 3, W1, W2, W4, and W6; long-term safety and preliminary efficacy are evaluated on W13, W26 and W52.

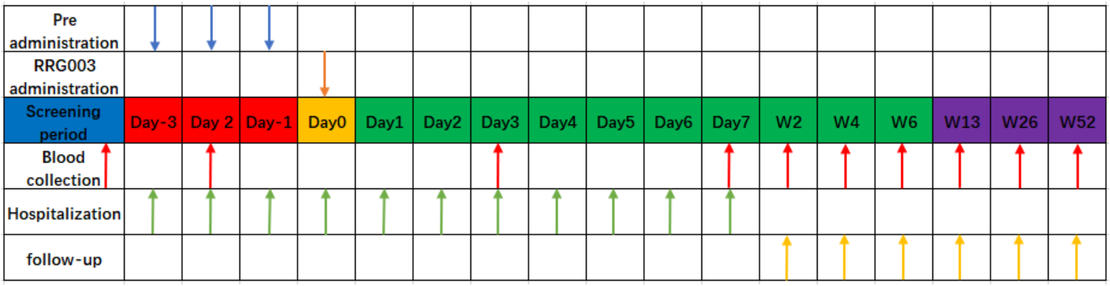

21. Follow-up and medical care after completing the trial

Follow-up visits will continue every six months after completion of the trial.

22. Responsibilities of the parties and other relevant regulation

Principal investigator: Seriously perform the duties of the investigator, agree to conduct the clinical trial in accordance with the design and specifications of this protocol, and explain the relevant data, regulations, and responsibilities of the trial in detail to all personnel in the trial. Sufficient time should be guaranteed to carry out and complete the clinical trial within the prescribed period, and all relevant information and data will be truthfully, accurately, completely, timely, and legally entered into the medical records and case report forms. Strictly abide by the Declaration of Helsinki, explain the details of the clinical trial approved by the Ethics Committee to the subject's guardian who acquires informed consent. Be responsible for making medical decisions related to this clinical trial and ensure that subjects receive appropriate treatment in the event of adverse events during the trial. If a serious adverse event occurs during the trial, appropriate treatment will be taken immediately to ensure the safety of the subject, which will be reported to the relevant authorities specified in the GCP.

Sponsor: Responsible for initiating, applying, organizing, supervising, and reviewing the clinical trial and providing trial funding in accordance with GCP standards. In particular, provide therapeutic

compensation for subjects who suffer trial-related damage or death during the trial and provide legal and economic guarantees for the investigators.

Reference

1. Deafness and hearing loss. 2021. at https://www.who.int/news-room/fact-sheets/detail/deafness-and-hearing-loss.)
2. Zhang QJ, Han B, Lan L, et al. High frequency of OTOF mutations in Chinese infants with congenital auditory neuropathy spectrum disorder. Clinical genetics 2016;90:238-46.
3. Akil O, Dyka F, Calvet C, et al. Dual AAV-mediated gene therapy restores hearing in a DFNB9 mouse model. Proceedings of the National Academy of Sciences 2019;116:4496-501.
4. Al-Moyed H, Cepeda AP, Jung S, Moser T, Kügler S, Reisinger E. A dual-AAV approach restores fast exocytosis and partially rescues auditory function in deaf otoferlin knock-out mice. EMBO Molecular Medicine 2019;11:e9396.

Protocol Amendments

Document	Date
Version 2.0	24 June 2022
Version 5.0	6 July 2023

Summary of the major changes to the trial protocol to make the trial protocol more reasonable and operationally feasible taking account of the clinical risk/benefit for participating subjects.

Description of changes and rationale for changes are as follows:

3. Study design

Considering the clinical risk/benefit and no DLT occurring in the dosage group of 30 μL and 50 μL, 30 μL or 50 μL dose group can be expanded.

Hearing recovery in bilateral ears can benefit sound localization of patients, so we expanded the trial to double injection (including bilateral administration), after confirming the safety and efficacy.

4. Subject inclusion criteria, exclusion criteria and suspension criteria

Expand the range of participant's age (from 3-10 years to 1-18 years) for potential benefits of younger and older children due to no DLT occurring in treated patients.

5. The number of cases required to achieve the intended purpose of the study based on statistical principles

To explore and expand the clinical benefits to more patients, we increased the number of participants (from 2-3 to 4-12) due to no DLT occurring and robust hearing recovery.

7.3.5 Speech recognition

To make speech evaluation more suitable for children at different ages and consider operative tools, do not use Sun Xibin's methods and add Auditory Performance Rating Scale (CAP) and Speech Intelligibility Rating Scale (SIR).

7.4 Additional indicators

To evaluate the therapeutic efficacy and effect on patients comprehensively, add near-infrared light functional imaging, electroencephalogram (EEG), music test, and growth and development scales.

20. Expected progress and completion date of clinical trial

To monitor long-term safety, add the time points (at 26 and 52 weeks) for blood collection.
